# Abstracts of the Fifth International Symposium on Molecular Insect Science: May 20–24, 2006, Tucson, Arizona USA, Organizing Committee

**DOI:** 10.1673/2006_06_45.1

**Published:** 2006-12-05

**Authors:** Michael Adams, Giovanni Bosco, David Denlinger, Tarlochan Dhadialla, Linda Field, John Hildebrand, Anthony James, Michael Kanost, Nancy Moran, Alexander Raikhel, David Sattelle, Nicholas Strausfeld, Judith Willis, Mariana Wolfner

**Affiliations:** ^a^ University of California, Riverside; ^b^ University of Arizona; ^c^ Ohio State University; ^d^ Dow AgroSciences LLC; ^e^ BCH Division, Rothamsted Research; ^f^ University of Arizona; ^g^ University of California, Irvine; ^h^ Kansas State University; ^i^ University of Arizona; ^j^ University of California, Riverside; ^k^ University of Oxford; ^l^ University of Arizona; ^m^ University of Georgia; ^n^ Cornell University

## Abstracts are listed in alphabetical order by the last name of the senior author

### Vitellogenesis in *Dipetalogaster maxima*, a vector of Chagas’ disease, *Dipetalogaster maxima*

S.A. Aguirre, L. Garcia, S. Frede, L. Sosa, L.E. Canavoso and E.R. Rubiolo.

Departamento de Bioquímica Clínica, CIBICI-Conicet. Facultad de Ciencias Químicas, Universidad Nacional de Córdoba. Córdoba, Argentina. CP 5000. Correspondence: lcanavo@mail.fcq.unc.edu.ar

Hematophagous insects, like vectors of Chagas’ disease, must surpass a threshold level in terms of the amount and quality of the blood meal in order to successfully produce eggs. During this process, the fat body synthesizes the main egg protein, vitellogenin (Vg), which will be taken up by the ovary to be stored as vitellin (Vt). Vg and Vt have been isolated from several species and their levels are indicative of the efficiency of the oogenesis process. However, information about their regulation in Chagas’ disease vectors is still scarce. In this work, therefore, we have analyzed in anautogenous and autogenous females of *Dipetalogaster maxima* (Hemiptera:Reduviidae): a] the kinetic of Vg synthesis in fat body, b] Vg levels in hemolymph, and c] the stores of Vt in ovary. Vg synthesis and its levels in hemolymph were measured by western-blot and enzyme-linked-immunoassay (ELISA), respectively. The stores of Vt in ovary were analyzed by immunohistochemistry. In anautogenous insects the study was performed between 2–8 days post-ecdysis and between 2– 20 days post-blood feeding. In addition, autogenous females were studied between 2–15 days post-ecdysis. During the post-ecdysis period, anautogenous *D. maxima* showed decreased synthesis of Vg and concomitantly, low levels of Vg in hemolymph (5.5x10^−3^ μg/μl at day 4). After a blood meal, Vg synthesis in fat body and its levels in hemolymph increased significantly, reaching at day 20 post-feeding approximately 19 μg/μl. Histological and immunohistochemical studies of the ovaries were in agreement with the biochemical findings, especially the development of the tissue from day 2 post-blood feeding as well as the accumulation of Vt in developing oocytes. Autogenous insects displayed a pattern for Vg and Vt quite different compared with anautogenous females, characterized by a decreased Vg synthesis and a poor development of the ovary. The levels of Vg in hemolymph during the period post-ecdysis fluctuated between 1.5– 4.0x10^−3^ μg/μl. This was sufficient however to produce at least one batch of eggs. Supported by Secyt-UNC (E.R.R) and CONICET-Argentina (L.E.C).

### The arginine vasopressin-like peptide and its receptor in *Tribolium castaneum*

Michael J Aikins^1^, Khurshida Begum^1^, David A. Schooley^2^, Yoonseong Park^1^

^1^ Entomology, Kansas State University, Manhattan, Kansas. Correspondence: ypark@ksu.edu

^2^ Biochemistry and Molecular Biology, University of Nevada, Reno, Nevada.

Arginine vasopressin and its related peptides are well known for their pivotal function in vertebrates for diuresis, reproductive, and neural functions. The arginine vasopressin-like peptide (AVPL) in insect was originally described in the locust *Locusta migratoria*, whereas a similar sequence is absent in the genome of higher dipteran insects including *Drosophila, Anopheles, Apis, and Bombyx*. Analysis of the genome sequence of *Tribolium castaneum* (Coleoptera:Tenebrionidae) in BeetleBase (http://www.bioinformatics.ksu.edu/BeetleBase/) identified a putative *avpl* gene and a gene encoding a novel G protein-coupled receptor as a putative receptor for the AVPL. A pair of cells on the ventral surface of subesophageal ganglion expresses the AVPL. We found that the monomeric form of AVPL is highly potent on the receptor expressed in a heterologous expression system, suggesting an authentic ligand-receptor interaction. Bioactivity of the AVPL was tested for its diuretic activity. Injection of the monomeric AVPL in the adult *Tribolium* induced immediate diuresis implying that the AVPL functions as a diuretic peptide in *Tribolium*.

### Proteomics to identify odorant binding proteins (OBPs) and chemosensory proteins (CSPs) from the antennae and tarsi of *Tribolium brevicornis*

Taofic Alabi ^1^ Frederic Francis^1^ Edwin De Paw^2^ and Eric Haubruge^2^

^1^Functional and Evolutionary Entomology, Gembloux Agricultural University, Passage des Deportes 2, 5030 Gembloux, Belgium

^2^ Mass Spectrometry Laboratory, University of Liège, BAT. B6 Chimie physique, allee de la Chimie 3, 4000 Liege 1, Belgium, Correspondence: haubruge.e@fsagx.ac.be

Chemoreception in insects is mediated by small soluble proteins that are abundantly present in the aqueous lymph of chemosensilla and that interact with odorant molecules and pheromones on their way to and from olfactory receptor. Two major classes of such proteins have been described: odorant binding proteins (OBPs) and chemosensory proteins (CSPs). A proteomic approach based on two-dimensional polyacrylamide gel electrophoresis (2-D PAGE), in which proteins are separated according to charge (pI) by isoelectric focusing (IEF) and according to size (Mr) by SDS-PAGE, was performed for the resolution of complex mixtures of proteins from antennae and Tarsi of *Tribolium brevicornis*. The proteins were then silver-stained and analysed by Matrix assisted laser desorption time of flight MS (MALDI-TOF) or by Electrospray (ESI) coupled with tandem Mass Spectrometry (MS-MS). Proteins from this *Tribolium* species was found to present sequence similarities to OBPs and CSPs recently discovery in several other insect orders. Development of proteomic studies was discussed in term of efficiency in functional and evolutional entomology.

### Application of insect genomics in the identification of resistance mechanisms and novel target sites

A. P. Alves^1^, M. D. Lorenzen ^2^, R. W. Beeman ^2^, and B. D. Siegfried^1^

^1^Department of Entomology, University of Nebraska – Lincoln, Lincoln, NE, USA, 68583-0816. Correspondence: anaalves@unlserve.unl.edu

^2^Grain Marketing and Production Research Center, ARS-USDA, Manhattan, KS, USA, 66502

*Bacillus thuringiensis* (Bt) is a valuable source of insecticidal proteins for use in insect pest control either in conventional spray formulations or in transgenic crops. However, the evolution of insect resistance in field populations is an important threat to this technology especially with transgenic plants that express the Bt toxins. The western corn rootworm (WCR), *Diabrotica virgifera virgifera* LeConte, one of the insect pests targeted with Bt transgenic plants, has displayed an amazing capacity to develop resistance to most management strategies, including soil insecticides, behavioral resistance to crop rotation, and foliar adulticides. Therefore, a critical need exists for new and effective management options. The objective of this project is to develop a system to identify genes and pathways important as possible target sites and in conferring insecticide resistance to WCR. To conduct this study, disruption of selected genes will be obtained through RNA interference (RNAi), based on the synthesis and injection of gene specific double stranded RNA. To validate the RNAi technique in WCR, silencing of WCR *laccase*, a gene involved in cuticle tanning, was successfully obtained in our lab. Gene silencing can be visualized by sustained lack of pigmentation of injected larvae after molting. This research will provide the basis for conducting large-scale identification of genes related to insect resistance and to vital pathways representing potential insecticide target sites.

### Regulation of genetically-based variation in juvenile hormone esterase activity in cricket, *Gryllus assimil*

A.N. Anand, E.J. Crone, A.J. Zera

School of Biological Sciences, University of Nebraska, Lincoln, NE 68588-0118, USA. Correspondence: aanand2@unl.edu

A key step in understanding the evolution of hormonal control is the characterization of genetically-based variation and co-variation of endocrine traits. Previously, natural genetic variation was identified in an enzyme juvenile hormone esterase (JHE). JHE degrades and partially regulates the titer of the key insect developmental hormone, juvenile hormone (JH). Artificial selection produced replicated lines differing 8 fold in hemolymph JHE activity in *Gryllus assimilis*. Current study was undertaken to identify the variable molecular and physiological regulators responsible for JHE activity differences between the lines. Analysis of fat body and mid gut tissues, known to express *Jhe* gene, revealed that higher hemolymph JHE activity in high line was specifically correlated to increased fat body JHE activity. Also, no difference in juvenile hormone epoxide hydrolase (another JH degrading enzyme) activities was observed between the lines in either tissue. Age profile for *Jhe* gene transcript levels was determined for the tissues during the last larval stadium. Higher *Jhe* gene transcript levels in high selected line can partially explain the higher hemolymph JHE activity. Work is in progress to determine whether difference in *Jhe* gene transcript levels is due to cis-linked (e.g. promoter) vs. trans-unlinked (e.g. neurohormonal) regulators, using cross and intercrossed inbred lines. This is the first study to look at the molecular and physiological causes of genetically-based variation in an endocrine regulator in natural populations.

### Specific interactions amongst classic and Plus-C odorant binding proteins of the African malaria vector *Anopheles gambiae*

Evi Andronopoulou^1^, Vassiliki Labropoulou^1^, Vassilis Douris^1^, Daniel F. Woods^2^, Harald Biessmann^3^ and Kostas Iatrou^1^

^1^Insect Molecular Genetics and Biotechnology Group, Institute of Biology, National Centre for Scientific Research “Demokritos”, 153 10 Aghia Paraskevi Attikis (Athens), Greece. Correspondence: iatrou@bio.demokritos.gr

^2^Inscent Inc., Irvine, CA 92614, USA; ^3^Developmental Biology Center, University of California, Irvine, CA 92697, USA.

We discuss results from a comprehensive study undertaken to deduce interactions between various antennal proteins of the African malaria vector *Anopheles gambiae* with a specific focus on the interactions among odorant binding proteins (OBPs). From an initial screen for proteins that interact with a member of the Plus-C group of odorant binding proteins, OBP48, which is primarily expressed in female antennae and down regulated after a blood meal, a number of interacting proteins were identified, which included five classic OBPs and OBP48 itself. The interacting OBPs as well a number of other classic and Plus-C group OBPs that were not identified in the initial screen, were expressed in lepidopteran cells and subsequently examined for *in vitro* interactions in the absence of exogenously added ligands. Co-immunoprecipitation and chemical cross-linking studies suggest that OBP48 is capable of homodimerizing, heterodimerizing and forming higher order complexes with those examined examples of classical OBPs identified in the initial screen but not with other classical or Plus-C group OBPs that failed to appear as interacting proteins in the screen. The latter OBPs are, however, also capable of forming homodimers *in vitro* and, at least in the case of two examined classic OBPs, heterodimers as well. These results suggest a previously unsuspected potential of non-random combinatorial complexity that may be crucial for odor discrimination by the mosquito.

### An RNAi-based study of the metabolic pathways for cuticle sclerotization and pigmentation in *Tribolium castaneum*

Y. Arakane ^1^, J. Lomakin ^2^, S. Muthukrishnan ^1^, R. W. Beeman ^3^, S. H. Gehrke ^2^, M. R. Kanost ^1^ and K. J. Kramer ^1, 3^

^1^ Department of Biochemistry, Kansas State University, Manhattan, KS 66506. Correspondence: arakane@gmprc.ksu.edu

^2^ Department of Chemical and Petroleum Engineering, University of Kansas, Lawrence, KS 660452

^3^ Grain Marketing and Production Research Center, ARS-USDA, Manhattan, KS, 66502 USA

Insect cuticle tanning (sclerotization and pigmentation) is a complex process that involves the production of quinones and quinone methides from catechols, followed by their oxidative conjugation with cuticular proteins, a process that leads to cuticle hardening and darkening. Using RNA interference (RNAi) methodology, we previously demonstrated that laccase 2 is the enzyme catalyzing cuticle tanning in the red flour beetle, *Tribolium castaneum*. By tblastn analysis of the *Tribolium* genome, conducted through Beetlebase, we identified several genes probably involved in the synthesis of catechols that are potential laccase 2 substrates. These genes include *dopa decarboxylase* (*DDC*), *dopamine N-acetyltransferase* (*NAT*) and *aspartate* □*-decarboxylase* (*black*). To further clarify the metabolic pathways responsible for cuticle tanning and to determine the influence of these genes and different catechols on sclerotization and pigmentation, double stranded RNAs (dsRNAs) for *DDC*, *NAT* and *black* were injected into *Tribolium* larvae and the resulting changes in morphology, pigmentation, and mRNA levels were determined. Finally, dynamic mechanical analysis was conducted to measure physical properties of elytral cuticle obtained from body color-mutant strains and dsRNA-treated insects. A metabolic pathway for *Tribolium* cuticle sclerotization and pigmentation will be presented. Supported in part by the National Science Foundation (MCB-0236039 and IBN-0316963).

### Identification of the gene encoding laccase of the silkworm, *Bombyx mori*: Purification, analyses of cDNA sequence, expression pattern and recombinant protein

T. Asano, H. Yamazaki, and S. Izumi

Department of Biological Sciences, Tokyo Metropollitan University, Minamiohsawa 1-1, Hachioji city, Tokyo, JAPAN. Correspondence: asano-tsunaki@comp.metro-u.ac.jp

The laccase-type phenoloxidase (laccase) that is present in the cuticle matrix has different enzymatic properties from tyrosinase type phenoloxidase for melanin synthesis. It is thought that the laccase plays an important role in cuticle formation, since it catalyzes the oxidation of phenolic compounds such as N-acetyl dopamine and *N-*□*-*alanyl dopamine to corresponding quinones, which is regarded as the key process of the quinone-tanning for cuticle sclerotization. Though insect laccases have been purified from several species, little is known about their structures. Recently, cDNA encoding a protein (laccase-like oxidase) that has the catalytic domain specific to laccases from other organisms such as bacteria or plants was cloned from the tobacco hornworm, *Manduca sexta*. Furthermore, the RNAi studies of the red flour beetle, *Tribolium castaneum*, revealed that *laccase 2* functions in hardening and darkening of the cuticle. However, the properties of their gene products have not been characterized yet at the protein level. To clarify the relationship between laccase protein and laccase genes, we purified laccase from the pupal cuticles of the silkworm, *Bombyx mori* and investigated its partial amino acid sequences by mass spectrometry. The tryptic fragments were assigned to those predicted from the laccase-like oxidase gene of *M. sexta*, and finally an EST clone of *B. mori* laccase was found in a database. This clone encodes 91 kDa protein that shows high similarity (75 ~ 90 %) with proteins encoded in *laccase 2* genes from other insects. The expression pattern was also similar to those of other insect laccase genes. The high level of expression was detected just before the ecdysis. Considering that the laccase activity is detected after ecdysis, this observation indicates that laccase accumulates in the new cuticle as an inactive precursor form and is activated after ecdysis. In order to test this hypothesis, we are currently undertaking the analysis of recombinant laccase protein.

### Small heat shock proteins of the silkworm, *Bombyx mori*

Y. Aso ^1^, D. Sakano ^2^, B. Li ^1, 2^, Q.-Y. Xia ^1, 2^, and H. Fujii ^1^

^1^ Faculty of Agriculture, Kyushu University, Fukuoka 812-8581, Japan. Correspondence: yaso@agr.kyushu-u.ac.jp

^2^ College of Sericulture and Biotechnology, Southwest University, Chongqing 400716, China.

Alpha-crystallin is one of lenticular proteins in mammals and has a C-terminal beta-strands-rich domain. Small heat shock protein (sHSP) is a ubiquitous family of 15–42 kDa polypeptides having a similar C-terminal domain to that of the alpha-crystallin. sHSP has been recognized to play important roles in a variety of physiological events, although details have not yet been clarified. The most documented sHSP of insects is that from the *Drosophila melanogaster*, but little information on lepidopteran sHSP’s are so far available. Results presented here were from studies on sHSP’s of the silkworm, *Bombyx mori. shsp*19.9, 20.1, 20.4, 20.8, 21.4, and 23.7 cDNA’s encoded sHSP’s having molecular sizes of 19.9, 20.1, 20.4, 20.8, 21.4, and 23.7 kDa, respectively. sHSP21.4 was notably different from other sHSP’s, based on all results from examinations so far done. Deduced amino acid sequence of sHSP21.4 was similar to that of the *D. melanogaster* CG14207-PA (DmCG), whereas the sequences of other five were quite similar to each other. sHSP20.8 was highly similar to sHSP from the Indianmeal moth, *Plodia interpunctella* (PI). The occurrence and alignment of Cys residue was characteristic. Each of sHSP20.8 and PI had a N-terminal Cys, and these overlapped. Each of sHSP19.9 and 20.1 also had a C-terminal Cys, and these also overlapped. sHSP23.7 had three Cys residues; two in a Cys-Pro-Cys might play a role in oxido-reduction reaction. Neither sHSP20.4, sHSP21.4, nor DmCG had any Cys residues. The transcriptions of all the *B. mori shsp*’s were constitutive, and transcripts were widely distributed in a variety of tissues, although their amounts were low. A heat shock triggered an increase in transcription of a *shsp* except *shsp*21.4. Results from phylogenetic analysis also suggested that the *B. mori* sHSP’s are grouped into at least two classes: sHSP21.4 and other five sHSP’s.

### Molecular characterization of genes associated with oogenesis and milk production in the tsetse fly (*Glossina morsitans morsitans*)

Geoffrey M. Attardo^1^, Nurper Guz^1^, Patricia Strickler-Dinglasan^2^, Serap Aksoy^1^

^1^ Yale School of Public Health and Epidemiology, 606 LEPH, 60 College Street, New Haven, CT 06520. Correspondence: geoffrey.attardo@yale.edu

^2^ Center for Marine Biotechnology, University of Maryland Biotechnology Institute

Tsetse flies (Glossina sp.) vector African trypanosomiasis, a disease for which over 60 million people are at risk in sub-Saharan Africa. Tsetse flies are viviparous and are unique in that the adult flies carry their young in utero for the duration of their embryonic and larval development. Pregnant flies supply their larvae with nutrients in the form of a “milk” substance secreted from a modified accessory gland. Flies give birth to a fully developed third instar larvae which pupate shortly after birth. This line of research focuses on the dynamics of two gene products associated with reproduction during the first and second gonotrophic cycles of the tsetse fly. The gene products studied are a previously identified putative yolk protein (*gmYp*) and a gene bearing homology to a protein found in tsetse “milk” secretions (*gmMGP*). Stage and tissue specificity of *gmYp* expression shows that the transcripts for this gene are exclusively in the reproductive tract of the fly at the same time as oogenesis is occurring suggesting that this gene is acting as a vitellogenic protein. Expression analysis of *gmMGP* shows that transcripts for this gene become detectable in parallel with larvigenesis. Transcripts for *gmMGP* are specific to the fat body and milk gland tissues. GMMGP protein appears in the mother during larvigenesis and is transferred to the larvae over the duration of pregnancy. Immunohistochemical analysis of GMMGP shows it to be exclusively found in the milk gland of the mother. Staining is throughout the gland and leads directly to its entrance to the uterus where the mouthparts of the larvae are positioned. These results indicate that these gene products are involved in tsetse oogenesis (gmYp) and larvigenesis (gmMGP). These genes can be used as markers for further studies in tsetse reproduction in regard to the effects of symbiosis and trypanosome infection.

### Sex-specific expression mediated by the *cis*-acting control elements of the *AeAct-4* gene of the yellow fever mosquito, *Aedes aegypti*

Diane H. Aw^1^, Nijole Jasinskiene^1^, Rosemary S Burton^2^, Matthew J Epton^2^, Hoang Kim Phuc^2^, Guoliang Fu^3^, Thomas U. Berendonk2*, Aurora Ashykian^1^, Osvaldo Marinotti^1^, Luke Alphey^2, 3^ and Anthony A. James^1,4^

^1^Department of Molecular Biology and Biochemistry, University of California, Irvine, California 92697-3900, USA. Correspondence: njasinsk@uci.edu

^2^Department of Zoology, University of Oxford, South Parks Road, Oxford OX1 3PS, UK

^3^Oxitec Ltd., 71 Milton Park, Abingdon, OX14 4RX, UK

^4^Department of Microbiology and Molecular Genetics, University of California, Irvine, California 92697-3900, USA

*Present address: Universität Leipzig, Institut für Zoologie Molekulare Evolution und Systematik der Tiere, Liebigstr.18, D-04103 Leipzig, Germany

Various means of reducing populations of vector insects have led to decreases in transmission of the pathogens that cause diseases such as dengue fever and malaria. Recent approaches include the development of transgenic mosquitoes to express dominant conditional lethal genes whose expression is driven by stage- and sex- specific DNA control elements. The *Aedes Actin-4* gene (*AeAct-4*) was demonstrated previously to be expressed only in female pupae of the yellow fever mosquito, *Aedes aegypti*. The *AeAct-4 cis*-acting control DNA was cloned into the *Mos I mariner* transposable element and used to drive expression of the *DsRed* reporter gene. While both male and female pupae transcribe some mRNA, only adult females were found to accumulate DsRed protein. This transcriptional and translational regulation may be exploited to use the *AeAct-4* control sequences to drive expression of a lethal gene in mosquitoes.

### The frontal ganglion in insect ecdysis: A novel early role for Crustacean cardioactive peptide

Amir Ayali

Department of Zoology, Tel Aviv University, Tel Aviv 69978, Israel. Correspondence: ayali@post.tau.ac.il

Chemical modulation is well established as an important factor in the generation and control of motor patterns and behavior. Insect ecdysis offers an attractive model for the intricate interactions between endocrine and neural control. The molt-related behavior is composed of a series of motor patterns whose timing must be precisely coordinated to ensure the proper execution of its vital outcome. A considerable amount of knowledge has accumulated on the role of peptidergic modulators in inducing and controlling the different motor patterns. Yet, the picture in different insect groups is far from complete. We focus on the locust frontal ganglion (FG) and the neuronal circuit(s) within it, as a previously unexplored target for ecdysis peptides and molt-related modulation. Our recently reported results suggest that the FG is a key player in insect molting. In our current study we show that Crustacean cardioactive peptide (CCAP), previously reported to trigger ecdysis motor patterns, induce a dose-dependent increase in the frequency of the FG rhythmic activity. The modulatory effects of CCAP on the FG motor circuit are dependent on behavioral state and physiological context; The FG shows maximal competence to CCAP modulation during the molt. We suggest that this context dependency is the result of ETH acting on the FG prior to CCAP. Pre-treatment of an isolated ganglion (dissected from a non-molting animal) with ETH cause the CCAP-induced effects to be similar to those induced by CCAP alone during pre-ecdysis. The CCAP induced excitation can be also demonstrated in the intact animal, where it is accompanied by the appearance of air bubbles in the locust gut. Early low hemolymph concentrations of ETH are thought to precede CCAP release *in vivo*. Our *in-vitr*o and *in-vivo* results indicate on a novel role for CCAP in generating air-swallowing behavior during the early stages of ecdysis. This is also supported by distinct changes in the profile of CCAP immunoreactivity in tritocerebral CCAP cells and their axonal arborisation in the FG neuropile. The CCAP increase in these cells precedes its peak in abdominal ganglia. Finally, it was previously observed in the abdominal ganglia of *Manduca* and elsewhere that molt-related CCAP release is cGMP dependent. No elevation of cGMP immunoreactivity is observed in the tritocerebrum CCAP cells innervating the FG at the different stages of the locust molt.

### Characterization of insect EcR and USP in a mammalian cell culture system

Josh Beatty^1^, Jenna Callender^1^, Torsten Fauth ^1,2^, Cary Weinberger^1^, Margarethe Spindler-Barth^2^, and Vincent C. Henrich^1^

^1^Biotechnology and Genomic Research Center. University of North Carolina-Greensboro, Greensboro, NC 27402-6170. Correspondence: jmbeatt2@hotmail.com

^2^Dept. of General Zoology and Endocrinology, University of Ulm, Germany

The functional ecdysteroid receptor in *Drosophila melanogaster* and other insects is a heterodimer composed of the ecdysone receptor (EcR) and Ultraspiracle (USP). Combinations of three natural EcR isoforms and USP fusion proteins have been tested in a mammalian cell culture system (Chinese hamster ovary) for transcriptional activity, ligand-binding, and binding to the canonical inverted repeat ecdysone response element (EcRE), *hsp27*. In this system, the transcriptional activity of a luciferase reporter gene whose promoter contains tandem repeats of the *hsp27* EcRE is elevated by 10-fold or more in the presence of micromolar amounts of muristerone A or 20-hydroxyecdysone. Both natural EcR isoforms and USP constructs dictate the level of transcriptional activity in both the absence and presence of ecdysteroids, indicating that both hterodimeric partners play a role in determining the level of transcriptional activity. For instance, an USP construct lacking its DNA-binding domain maintains a normal and high level of activity with EcRB1, but activity is drastically reduced with EcRA and EcRB2, suggesting strongly that the nature of the heterodimeric interaction varies among the isoforms. Juvenile hormone and other analogues fail to elevate transcriptional activity on the cell culture assay, but in conjunction with ecdysteroids, these compounds reduce the maximal dosage of ecdysteroids by about tenfold. The capacity for JH potentiation varies among the EcR isoforms and different USP construct. Site-directed mutagenesis further indicates that some amino acid substitutions in shared regions of both EcR and USP differentially affect heterodimeric transcriptional activity, presumably because isoform-specific transcriptional complexes involve different interactions with EcR and USP. The diversity of EcR/USP capabilities is further suggested by differences in transcriptional activity among receptors from different species. Collectively, the mutations provide an experimental basis for assessing specific receptor functions.

### Annotation of the *Tribolium* genome: A progress report

R.W. Beeman^1^, Marcé D. Lorenzen^1^, S. Richards^2^, G. Weinstock^2^, R. Gibbs^2^ and S.J. Brown^3^

^1^USDA-ARS-GMPRC, 1515 College Ave., Manhattan, KS, 66502, USA. Correspondence: richard.beeman@gmprc.ksu.edu

^2^Human Genome Sequencing Center, Baylor College of Medicine, 1 Baylor Plaza, Houston, TX, 77030, USA

^3^Division of Biology, Kansas State University, Manhattan, KS, 66506, USA.

The 165 Mb euchromatic genome sequence of *Tribolium castaneum* consists of 420 scaffolds, containing >95% of a sample of known genes. We have anchored 80% of the sequenced genome to the linkage groups at ~1 cM resolution using SSCP-based recombinational mapping. Alternative sets of gene predictions derived from Genscan, Augustus, FgenesH, Geneid, NCBI, and Ensembl have been compiled at the Human Genome Sequencing Center, and a Glean statistical analysis and aggregation of these sets has been conducted (Aaron J. Mackey, U. Penn.). Currently the gene count is approximately 16,000 genes, of which ~ 9,500 have supporting evidence from both the Ensembl (run at the HGSC) and NCBI Gnomen annotation pipelines. The genome sequence and associated annotation tracks can be viewed in the Genboree genome browser (www.genboree.org) developed at the Baylor College of Medicine, and any desired sequences can be retrieved either directly or via links to NCBI. These computer predictions form a preliminary annotation that will be further amended and refined manually. Annotation teams have been organized for several gene families. Genome tiling and gene transcript arrays are being developed at several locations to facilitate the genome annotation and to support gene expression studies. RNA interference and transposon-mediated mutagenesis will help elucidate functions of predicted genes. BeetleBase (http://www.bioinformatics.ksu.edu/BeetleBase/) will be the final repository

### Cytochrome P450 genes in insects–an embarrassment of riches

May Berenbaum

University of Illinois at Urbana-Champaign. Correspondence: maybe@uiuc.edu

The genes encoding cytochrome P450 monooxygenases, heme-thiolate enzymes that catalyze the NADPH- associated reductive cleavage of oxygen to produce a functionalized product and water, constitute one of the largest superfamilies known, with over 5000 members identified and named (http://drnelson.utmem.edu/CytochromeP450.html). Although some P450 families are found in a wide variety of taxa, others are more restricted in distribution and accordingly are thought to have more specialized functions. The CYP6 family is unique to the Class Insecta and together with the CYP4 family represents half of the 90 P450s characterized in the genome of *Drosophila melanogaster* (Tijet et al. 2000). To date, *CYP6* genes in Lepidoptera have been implicated exclusively in metabolism of xenobiotics and are thought to be particularly important in metabolism of plant chemicals in this almost exclusively phytophagous group. How they have evolved and diversified, however, remains an open question. Possible mechanisms underlying this diversification are described for the subfamily CYP6B within the genus Papilio, the swallowtail butterflies, which radiated in the context of the toxic furanocoumarin-containing host families Rutaceae and Apiaceae.

### Gene expression in sensory tissues of the malaria mosquito, *Anopheles gambiae*

H. Biessmann^1^, Q. K. Nguyen^2^ D. Le^1^, R. Justice^3^ and M. F. Walter^1^

^1^Developmental Biology Center, University of California, Irvine, California 92697, USA. Correspondence: hbiessma@uci.edu

^2^Department of Biological Chemistry, University of California, Irvine, California 92697, USA

^3^Inscent, Inc, 17905 Sky Park Circle, Irvine, California 92614, USA

Female *Anopheles gambiae* mosquitoes respond to odors emitted from humans to find a blood meal, while males are nectar feeders. This complex behavior is controlled at several levels, but is probably initiated by the interaction of various molecules in the antennal sensilla. Important molecules in the early odor recognition events include odorant binding proteins (OBPs), which may be involved in odor molecule transport, odorant receptors (ORs) that are expressed in the chemosensory neurons and odor degrading enzymes (ODEs). To obtain a better understanding of the expression patterns of genes that may be involved in host odor perception in females, we have generated a custom microarray to study their steady state mRNA levels in chemosensory tissues, antennae and palps. Microarray results were validated by quantitative RT PCR. Our study identified several OBPs that are expressed at significantly higher levels in antennae and palps of females vs. males, while others showed the opposite expression pattern. Most OBPs are slightly down-regulated 24 hrs after blood feeding, but some, especially those with higher expression levels in males, are up-regulated in blood fed females, suggesting a shift in blood-fed females from human host seeking to nectar feeding. qRT PCR determinations of OBP mRNA levels in early and late pupae as well as in heads of 1 day-old and 4 day-old females and males also detected significant shifts in developmental expression profiles. Polyclonal antibodies against some OBPs have been generated that recognize the native proteins in mosquito heads. Supported by the U.S. Public Health Service grant AI051485 and by Inscent, Inc, Irvine, CA.

### A cell culture system and infectious clone for the study of *Rhopalosiphum padi* virus

S. Boyapalle^1^, R. Beckett^2^, N. Pal^1^, W.A.. Miller^2^, and B.C. Bonning^1^

^1^Department of Entomology Iowa State University, Ames, IA, USA. Correspondence: bonning@iastate.edu

^2^Department of Plant Pathology, Iowa State University, Ames, IA, USA

*Rhopalosiphum padi* virus (Dicistroviridae) (RhPV) is an icosahedral aphid virus with a 10 kb positive-sense RNA genome. We screened lepidopteran, dipteran and homopteran cell lines for susceptibility to RhPV following RNA transfection and observed CPE in homopteran cell lines derived from the glassy winged sharp shooter, *Homalodisca coagulata,* and the corn leaf hopper, *Dalbulus maidis*. Infection, viral replication and production of virions was confirmed by northern blot hybridization, RT-PCR, western blot analysis and immuno-electron microscopy. Full-length cDNA clones of RhPV were synthesized. RNA transcripts produced from one of the clones were infectious following transfection of the susceptible cell lines. Infection was confirmed by CPE and immuno-electron microscopy. Virions were purified from infected cells and fed to bird cherry-oat aphids, *Rhopalosiphum padi*. Aphids tested positive for infection by the RhPV clone by RT-PCR, western blot analysis and immuno-localization by light microscopy, two weeks after acquisition in three replicate experiments. The cDNA clone of the RhPV genome was inserted into the genome of *Autographa californica* multiple nucleopolyhedrovirus to create the recombinant baculovirus AcRhPV6. Expression of the RhPV genome in Sf21 cells resulted in formation of RhPV virus-like particles (VLPs) whose capsids are structurally and immunologically indistinguishable from the native virions. The presence of genomic RhPV RNA in recombinant baculovirus infected cells and in VLPs was confirmed by RT-PCR. Assembly of RhPV VLPs in the nucleus of baculovirus infected cells suggests that in Sf21 cells (1) both the 5′ and IGR IRES of RhPV are active, (2) the virus encoded protease is functional for processing of RhPV polyproteins, and (3) replication of RhPV is not required for encapsidation of RNA. For analysis of the infectivity of baculovirus expressed RhPV6, virions purified from baculovirus-infected Sf21 cells were fed to *R. padi*. Aphids were tested for infection by the baculovirus-produced RhPV clone by RT-PCR and western blot analysis, four weeks after acquisition. Baculovirus expression of RhPV in lepidopteran cell lines that do not support replication of RhPV provides a potential alternative approach for *in vitro* production of clones of this virus.

### Important interactions of *Bacillus thuringiensis* toxins with membrane receptors and their role in insect resistance

A.Bravo, I.Gomez, L.Pardo, C.Muñoz, C.Pérez, L.Fernandez and M.Soberón

Departamento de Microbiología Molecular, Instituto de Biotecnología Universidad Nacional Autónoma de México, Cuernavaca Morelos, Mexico. Correspondence: bravo@ibt.unam.mx

The insecticidal proteins produced by *Bacillus thuringiensis* (Bt), Cry toxins, are used to control insect pests. The primary action of Cry toxins is to lyse midgut epithelial cells by forming lytic pores in the apical membrane. Cry toxins are modular proteins comprised of three domains, domain I is involved in pore formation and domains II and III in receptor interactions. Here we summarize recent findings on the Cry-receptor interactions and their role in toxin action. Cry toxins interact sequentially with multiple receptors. In lepidopteran insects, Cry1A toxins interact initially with a cadherin-receptor. This interaction involves three different epitopes and promotes a final proteolytic processing of the toxin that induces the formation of a 250 kDa pre-pore structure, which has been suggested to be the responsible for the ionic pore-formation. The pre-pore showed 200 fold higher affinity to the second receptor, a glucosyl-phosphatidil-ionositol (GPI)-anchored Aminopeptidase N, this interaction leads to the insertion of oligomeric toxin into membrane microdomains (or lipid rafts) inducing cell swelling and insect death. Some authors suggested that a GPI-anchored Alkaline phosphatase could also participate in driving the pre-pore to lipid rafts. In addition, recent data shows that not only protein receptors are involved in Cry toxin interaction with membrane of susceptible organisms since certain glycolipids have a role in toxin action. These glycolipids are specific for insects or nematodes. The role of all these interactions in promoting insect resistance is of interest. In the case of mosquitocidal Cry toxins, we found that GPI-anchored proteins are also involved in binding Cry11Aa toxin. The case of mosquitocidal Bt toxins is very interesting since Bt subsp. *israelensis* (Bti) produces two different type of toxins, Cry and Cyt proteins, which together show a synergistic effect in their toxicity. In addition, no-resistance to Cry toxins has been selected in the presence of Cyt1A and Cyt1A overcomes insect resistance to different Cry toxins. We found that the molecular mechanism of synergism involves interaction of these two toxins and we identified the specific epitopes involved in this interaction. We will present data that show that Cyt1A synergizes or suppresses resistance to Cry toxins by functioning as membrane bound receptor. Bti is a highly effective pathogenic bacterium as it produces a toxin and also its functional receptor in the same crystal inclusion, promoting toxin binding to the membrane and avoiding the generation of insect resistance.

### Function and evolution of a mosquito salivary protein family

E. Calvo, B.J. Mans, J.F. Andersen and J.M. Ribeiro

Section of Vector Biology, Laboratory of Malaria and Vector Research, National Institute of Allergy and Infectious Diseases/NIH, 12735 Twinbrook Parkway, Rockville, MD 20852, USA. Correspondence: ecalvo@niaid.nih.gov

Saliva of blood-sucking arthropods contains a complex and diverse mixture of antihemostatic, antiinflammatory, and immunomodulatory compounds. The D7 salivary family of proteins is abundantly expressed in blood-feeding Diptera and is distantly related to the odorant-binding protein super family. In mosquitoes, two subfamilies exist, the long and short D7 proteins. Ticks and kissing bugs evolved salivary lipocalins that act as efficient scavengers of biogenic amines, and a similar function was postulated for the D7 proteins. Accordingly, we expressed the five members of the small D7 family of the African malaria vector Anopheles gambiae and a D7 long form from Aedes aegypti and showed by isothermal microcalorimetry, a modified and very sensitive non-equilibrium chromatography/spectrum distortion method, and by smooth muscle bioassay that four of these five short D7 proteins and the D7 long form bind serotonin with high affinity, as well as histamine and norepinephrine. The nonbinding D7 protein is poorly expressed in the salivary glands and appears to be on the path to becoming a pseudogene. Scavenging of host amines would antagonize their vasoconstrictor, platelet-aggregating, and pain-inducing properties. It appears that counteracting biogenic amines is of strong adaptive value in the convergent evolution of arthropods to hematophagy. This adaptation has been solved independently in ticks, bugs, and mosquitoes by co-option of either member of the lipocalin or, as shown here, by the odorant-binding protein families. This work was supported by the Division of Intramural Research, National Institute of Allergy and Infectious Diseases, National Institutes of Health.

### The short neuropeptide F-like receptor from the red imported fire ant, *Solenopsis invicta*

Mei-Er Chen and Patricia V. Pietrantonio

Department of Entomology, Texas Agricultural Experiment Station, Texas A&M University, College Station, TX 77843-2475, USA. Correspondence: p-pietrantonio@tamu.edu

In invertebrates, neuropeptide F (NPF) peptides share structural similarity with vertebrate neuropeptide Y, which regulates food consumption, circadian rhythms, anxiety and other physiological processes. The insect neuropeptide F receptors belong to the G protein-coupled receptor (GPCR) rhodopsin family. We have cloned the fire ant, *Solenopsis invicta* Buren (Hymenoptera: Formicidae), putative short NPF receptor using PCR and RACE methods. The complete 2185 bp cDNA encodes a 387-residue protein with a predicted GPCR seven transmembrane region structure. We propose that the sequence of the honey bee short NPF receptor, that has not yet been annotated, encodes a protein of 393 residues. In fire ant mated queens, receptor transcripts were detected in the brain, midgut, hindgut, Malpighian tubules, fat body and ovaries. The highest transcriptional expression was found in the brain. The downregulation of the fire ant short NPF receptor transcriptional expression in the brain with starvation suggests that the short NPF signal transduction cascade may play a role in feeding regulation in fire ant mated queens. *Arch. Insect Biochem. Physiol. 61: 195-208, 2006.*

### Eph-ephrin interactions establish a midline boundary for migrating neurons during the formation of the enteric nervous system in *Manduca sexta*

T.M. Coate^1^, T.L. Swanson^1^, A. Nighorn^2^, and P.F. Copenhaver^1^

^1^Dept. Cell & Developmental Biology L-215, Oregon Health & Science University, Portland, OR 97239. Correspondence: coatet@ohsu.edu

^2^Program in Neuroscience and Arizona Research Laboratories, Div. of Neurobiology, University of Arizona, Tucson, AZ 85721

Eph receptor tyrosine kinases and their ephrin ligands modulate the guidance of many developing neurons in the vertebrate nervous system, but overlapping expression patterns and promiscuous interactions among multiple ephrins and Ephs have hindered a functional analysis of specific ligand-receptor pairs *in vivo*. As an alternative strategy, we have investigated the role of ephrin-Eph interactions in the control of neuronal migration within the developing enteric nervous system (ENS) of the moth, *Manduca sexta*. During the formation of the ENS, a population of ~300 neurons (EP cells) migrates along a set of identified muscle band pathways on the midgut while avoiding adjacent interband regions. We have shown that the EP cells express a single GPI-linked ephrin (MsEphrin), which can be detected in their filopodial processes as they explore the midgut surface. Concurrently, the midline interband regions of the midgut (which are inhibitory to migration) express MsEph, the sole Eph receptor homologue in *Manduca*. Blocking endogenous MsEph receptors in cultured embryos with soluble MsEphrin-Fc fusion proteins induced abnormal midline crossing by the neurons and their axons. In contrast, treating the EP cells with soluble MsEph-Fc proteins inhibited their migration and outgrowth without inducing midline crossing. These results indicate that the expression of MsEph by the midline cells of the midgut normally prevents ectopic growth by the migratory EP cells across this interband boundary. They also suggest a novel role for reverse signaling via a GPI-linked ephrin ligand in the control of neuronal guidance. Previous *in vitro* studies have suggested that several different non-receptor tyrosine kinases (NRTKs) may be activated during reverse signaling by GPI-linked ephrins, but validation of these observations *in vivo* has been lacking. We are currently investigating the extent to which Src-family kinases and other NRTKs are coupled to MsEphrin-mediated reverse signaling in the EP cells as a mechanism for controlling the motile behavior and guidance of these neurons within the developing ENS.

### Paratransgenesis: constructing the enemy within

R. E. Collier^1^, C.Husseneder^1^, L. Foil^1^, R. Cooper^2^ and F. Enright^2^

^1^Department of Entomology, Louisiana State University Agricultural Center, Baton Rouge, LA 70803. Correspondence: rcollier@agcenter.lsu.edu

^2^Veterinary Science, Louisiana State University Agricultural Center, Baton Rouge, LA 70803

Paratransgenesis is the genetic manipulation of a host’s symbiotic microorganisms to achieve an array of objectives, ranging from disease eradication to control of the host organism. The application of paratransgenesis is promising in social insects because social interactions promote the exchange of microbes between colony members. Within the social insects, termites are known not only for their ecological and economical importance but for their close relationship with microbial symbionts. The hind gut of the Formosan subterranean termite (FST) provides a refuge for an array of protozoa and bacteria that fulfill important functions in the survival of their hosts, such as cellulose digestion. These symbionts are excellent tools and targets for paratransgenesis, which this study aims to employ in the control of FST. The goal of this study is to genetically engineer termite specific indigenous gut bacteria to secrete peptide toxins to target and kill the termite’s protozoa, which are responsible for the digestion of cellulose into metabolites subsequently utilized by the termite host. Without their protozoa and a supply of nutrients the termite dies. This study has shown that defaunation of termite guts using Metronidazole is followed by starvation and death of the termite host within six weeks. Peptide toxins were screened *in vitro* and *in vivo* for protozoicidal activity. *In vitro* tests on anaerobic cultures of protozoa confirmed activity of the peptides against the termite protozoa targets. *In vivo* tests using microinjection resulted in defaunation of the termite gut within 72hr of treatment with the selected toxins. The genes of the detrimental toxins have now been synthesized and a gene-shuttle system is presently being constructed to deliver, express and secrete the selected genes into the termite hind gut. The gene-shuttle is currently being optimized for inducible expression within the recently characterized termite specific anaerobic, gram positive bacterium *Pilibacter termitis*. The optimization of the gene-shuttle will ensure that environmental impacts are minimized and efficient colony level control of FST is achieved.

### A normal role for a nasty protein: the insect homologue of the Amyloid Precursor Protein (APP) regulates neuronal migration during embryonic development

P. F. Copenhaver, T. L. Swanson, L. M. Knittel, T. M. Coate, S. M. Farley, and M. A. Snyder

Department of Cell & Developmental Biology L-215, Oregon Health & Science University, Portland, OR 97239. Correspondence: copenhav@ohsu.edu

APP-like protein (APPL) is the insect homologue of vertebrate APP, a transmembrane protein that is aberrantly cleaved to produce the amyloid peptides associated with Alzheimer’s Disease. While both APP and APPL are abundantly expressed during neural development, their normal functions remain controversial. We have investigated the potential role of APPL as a neuronal guidance receptor in the enteric nervous system (ENS) of *Manduca sexta*, a preparation that permits manipulations of developing neurons *in vivo*. During the formation of the ENS, an identified set of neurons (EP cells) migrates along pre-formed muscle band pathways before extending axons and terminal synapses onto the gut musculature. We found that *Manduca* APPL (MsAPPL) is strongly expressed by the EP cells during their migration, while post-translational processing and trafficking of the holoprotein coincides with specific phases of neuronal differentiation. Provocative work has shown that human APP can regulate the heterotrimeric G protein Go□ in cell culture, suggesting that it may act as an unconventional G protein-coupled receptor. We found that *Manduca* Go□ co-immunoprecipitates with MsAPPL; the two proteins also co-localize within the leading processes of the EP cells, suggesting that they may directly interact. Activating Go□ within the EP cells inhibited their motility in a calcium-dependent manner, supporting the model that Go□-coupled receptors may mediate the response of migrating EP cells to inhibitory guidance cues. To test whether MsAPPL might act as such a receptor, we inhibited its expression in the developing ENS with antisense morpholinos; this treatment resulted in aberrant, ectopic migration of the EP cells onto the interband regions of the gut (normally inhibitory to migration). Treating the ENS with synthetic ectodomain fragments of MsAPPL (designed to interfere with its endogenous ligands) caused similar effects. Together, these results suggest that MsAPPL may act as a Go□-coupled receptor for one or more guidance cues that normally prevent the EP cells from migrating into inappropriate regions. We are currently testing whether MsAPPL regulates Go□ activation in the EP cells, and we are using an expression cloning strategy to screen for potential ligands of MsAPPL in the ENS. Supported by NIH R56 AG025525 and a grant from the Oregon Partnership for Alzheimer’s Research.

### Microevolution of endocrine regulation: *Jhe* transcript abundance underlies genetic variation in JHE activity

Erica J. Crone, Anurag Anand and Anthony, J. Zera

School of Biological Sciences, University of Nebraska at Lincoln, 1104 T St, Lincoln, NE 68588. Correspondence: ecrone2@unl.edu

Juvenile hormone acting in concert with the steroid hormone 20-hydroxy ecdysone is responsible for many essential developmental processes in insects. Many studies have shown that juvenile hormone levels in the hemolymph are under tight control, both by biosynthesis of the hormone and degradation of free hormone by the specific esterase juvenile hormone esterase (JHE). Hemolymph JHE activity from a species of field cricket, *Gryllus assimilis,* has been used as a model to study the microevolution of an endocrine trait. Selection for elevated or decreased hemolymph JHE activity showed that hemolymph JHE activity differences are heritable in this species. However, this and other work on the evolution of endocrine traits has primarily focused on the study of biochemical and physiological aspects and not the underlying molecular mechanism(s) controlling the microevolution of these traits. This study is attempting to address this deficit using hemolymph JHE activity in *G. assimilis* as a model. We have begun by looking for evidence that the heritable differences in hemolymph JHE activity are due to differential transcription of the *Jhe* gene. Evidence documents that transcript levels in the fat body and mid gut, tissue and hemolymph enzyme activity and an internal *Jhe* gene marker are correlated on one developmental day in two separately selected blocks of lines. This study shows that differences in *Jhe* transcript abundance are responsible for the heritable differences in hemolymph JHE activity in the selected lines. It is the first study to document the molecular basis for naturally occurring genetic variation in a hormonal regulator. Heritable differences in hemolymph JHE activity are also seen in a wing polymorphic species, *Gryllus firmus*, the molecular basis for these differences are also under investigation.

### The cloning of one putative octopamine receptor and two putative serotonin receptors from the tobacco hawkmoth, *Manduca sexta*

Andrew M. Dacks^1^. Joel B. Dacks^2^, Thomas A. Christensen^1^, Alan J. Nighorn^1^ 1

^1^Arizona Research Laboratories, Division of Neurobiology, The University of Arizona, Tucson, Arizona 85721, U.S.A. Correspondence: adacks@email.arizona.edu

^2^Department of Biological Sciences, The University of Calgary, Calgary, Alberta, T2N 1N4 Canada.

Serotonin (5HT) and octopamine (OA) are biogenic amines that are active throughout the nervous systems of insects, affecting sensory processing, information coding and behavior. As an initial step towards understanding the modulatory roles of these amines in olfactory processing we cloned one putative OA (MsOAR) and two 5HT receptors (Ms5HT1,R and Ms5HT1ijR) from the moth *Manduca sexta.* Phylogenetic analysis confirmed that MsOAR shares significant sequence homology with OA receptors previously characterized in the cockroach and the bee, but shows much less similarity to putative OA/tyramine receptors from the moths *Bombyx mori* and *Heliothis virescens.* Using the MsOAR sequence, fragments encoding putative OA receptors were obtained from 6. mori and *H. virescens,* and this analysis grouped these receptors in a separate clade with other identified tyramine receptors. Collectively, our results indicate that MsOAR is likely the first OA receptor cloned from a lepidopteran species. Ms5HT1jR and Ms5HT1jiR were both similar to 5HT1-type receptors from other invertebrate and vertebrate species but differed from each other in their N-terminus and 3^rd^ cytoplasmic loop. Ms5HT1,R was nearly identical to a 5HT receptor from *H. virescens* and MsSHTInR was almost identical to a 5HT receptor from *B. mori.* The sequences for homologs of Ms5HT1,R from *B. mori and* Ms5HT1,iR from *H. virescens* were also obtained and phylogenetic analysis of these data confirmed that the Lepidoptera likely have at least two 5HT1-type receptors.

### Molecular Identification of bursicon neurons in central nervous system of the tobacco hawkmoth, *Manduca sexta*

Li Dai^1^, Lisa Pitts^2^, Hans-Willi Honegger^2^, and Michael E. Adams^1^

^1^Departments of Entomology and Cell Biology/Neuroscience, University of California, Riverside, CA 92521, USA. Correspondence: lidai@ucr.edu

^2^Department of Biological Sciences, Vanderbilt University, Nashville, TN 37235-1634, USA.

During post-eclosion, adult insects undergo sequential processes of wing expansion, sclerotization and melanization under hormonal control. Bursicon, a key neurohormonal regulator of these behaviors, is highly conserved in the Insecta. Recent reports characterize bursicon as a pburs/burs heterodimeric cysteine knot protein in *Drosophila melanogaster*. We show the presence of two predicted proteins encoded by genes *Mas-burs* and *Mas-pburs* in *Manduca sexta. in situ* hybridization with *Mas-burs* and *Mas-pburs* DNA probes and immunohistochemistry with bursicon antibodies were used to label neurons, which express bursicon in the CNS of pharate larvae, pupae and adults. During development, the morphology and number of bursicon-expressed neurons in ventral ganglia changes during transitions through larva to pupa to adult stages. A cluster of intrinsic cells was identified in corpora cardiaca labeled only by pburs-specific DNA and antibody probes, and an additional pair of lateral cells in several abdominal ganglia were labeled only by a burs antibody probe. Using a recombinant bursicon protein, we observed that the pure hormone has dual functional roles in both wing expansion and tanning in *Manduca sexta*.

### Strategic expression of conserved ion transport peptide gene products in central and peripheral neurons of insects

Li Dai^1^, Dusan Zitnan^2^, and Mike E. Adams^1^

^1^Depts. of Entomology and Cell Biology/Neuroscience, University of California, Riverside, CA 92521, USA. Correspondence: lidai@ucr.edu

^2^Institute of Zoology, Slovak Academy of Sciences, Dúbravská cesta 9, 84506 Bratislava, Slovakia.

Structurally related ion transport peptides (ITP) and crustacean hyperglycemic hormones are increasingly implicated in diverse metabolic and developmental functions in arthropods. We have identified a conserved ITP gene encoding two peptides in *Manduca sexta, Bombyx mori* and *Aedes aegypti*: A C-terminally amidated ion transport peptide (ITP) and C-terminally unblocked ITP-like peptide (ITPL). *In silico* genomic DNA analysis indicates the ITP gene is conserved in other insect species. These peptides are expressed in two, regionally distinct neuronal populations. Mas-ITP expression is confined to the brain in five pairs of lateral neurosecretory cells (Type Ia2) projecting ipsilateral axons into the retrocerebral complex and 3-4 pairs of adjacent small lateral cells with extensive arborizations within the brain. Expression of Mas-ITPL is comparatively weak in the brain, but strong in the ventral ganglia and peripheral nervous system, where MasITP is absent. Mas-ITPL occurs in multiple bilaterally paired cells in the thoracic ganglia and one bilateral pair in each abdominal ganglion (AG1-9). In the peripheral nervous system, we find strong Mas-ITPL expression co-localized with crustacean cardioactive peptide (CCAP) in peripheral abdominal neurons (L1) of abdominal segments 2–7, which project axons into the transverse neurohemal nerves. Expanded cellular expression of MasITPL during metamorphosis consists of two additional pairs of small lateral neurons in the brain, and one pair of medial cells in each abdominal ganglion AG3-6. A similar pattern of ITP and ITPL expression was observed in the central and peripheral nervous systems of *Bombyx mori* and *Schistocerca americana*. These distinctive cellular expression patterns suggest that ITP and ITPL have evolved specialized physiological functions in arthropods.

### Isolation and characterization of a bombyxin-Like cDNA from *Manduca sexta*

L.J. D’Amico^1, 2^, H.F. Nijhout^1^

^1^Dept. of Biology, Duke University, Box 90338, Durham, NC 27708, USA

^2^Dept. of Biology, Northeastern University, 360 Huntington Ave. Boston, MA 02115, USA. Correspondence: l.damico@neu.edu

To fully understand the regulation of growth and size in an organism requires knowledge of the cellular mechanisms regulating growth processes and how those mechanisms are integrated at the organismal level with nutritional, metabolic, and endocrine factors. The insulin signaling pathway has been implicated as an important regulator of the growth of developing tissues as well as the overall size of an organism. Understanding the interaction between insulin signaling and the systemic regulation of growth requires a model organism amenable to the molecular study of insulin signaling, as well as characterization of the systemic regulation of body size. Here we describe the isolation of a cDNA from the tobacco hornworm, *Manduca sexta*, encoding a protein with similarity to the insect insulin-like neurohormone, bombyxin. A combination of degenerate PCR and 3′ and 5′ RACE yielded a 300bp amplicon sharing 58% identity at the protein level with a previously identified *Agrius* bombyxin-related peptide. Subsequent real-time PCR showed that transcript abundance increased in response to carbohydrate injection. Comparative homology modeling is used to generate a predictive 3D structure of our *Manduca* bombyxin. The work presented here is an important first step in developing tools for the molecular analysis of insulin signaling in an ideal lepidopteran research model. Research supported by a NSF Graduate Research Fellowship to LJD, and NSF award IBN0315897 to HFN.

### Temperature mediated modulation of vector competence of *Aedes aegypti* by influencing gut flora and intrinsic factors

Dileep N. Deobagkar^1,2^ Anjali D. Apte-Deshpande^2^, Mandar S. Paingankar^1^

^1^Department of Zoology, University of Pune, Pune -411007, INDIA. Correspondence: dndeo@unipune.ernet.in

^2^Institute of Bioinformatics and Biotechnology, University of Pune, Pune -411007, INDIA

*Serratia* sp. was found to be the common gut bacterium of *Aedes aegypti* larvae isolated from different natural environments and laboratory reared colonies. It was also retained in the guts of emerging adults by transtadial transmission. When *Serratia* was incorporated in blood meal along with dengue virus, there was statistically significant increase in number of susceptible *Ae. aegypti* females. We observed that *Serratia* specific 40 KDa polypeptide (P40) interacted with gut brush border membrane fraction containing dengue viral receptor component of *Ae. aegypti*. These interactions were further confirmed using dissected gut from *Ae. aegypti* females. P40 identified to be a periplasmic membrane component of *Serratia* thus seems to enhance the dengue viral interaction in the midgut of mosquito. We are attempting to use this microbe as a vehicle for paratransgenesis. Interestingly, the unique feature of this bacterium was its survival in the midgut of *Aedes* at higher ambient temperature. Expression of P40 was also enhanced at higher temperature. Higher temperature thus could assist *Serratia* mediated enhancement of viral susceptibility and therefore, vector competence of *Ae. aegypti*. Temperature is also known to modulate EIP as well as vector gene expressions. Using *Ae. aegypti* and *Ae. albopictus* derived cell lines we found that dengue-2 virus binding was enhanced on exposure to higher temperature. Temperature as an extrinsic factor thus can enhance the vector competence by modulating intrinsic factors including receptor gene regulation and also gut bacterium like *Serratia* sp.

### Anopheles anti-Plasmodium defense responses

George Dimopoulos

Dept. Molecular Microbiology and Immunology, Johns Hopkins School of Public Health, Baltimore, MD 21231, USA. Correspondence: gdimopou@jhsph.edu

Transmission of malaria requires successful completion of complex interactions between the *Anopheles* vector and the *Plasmodium* parasite. These interactions involve mosquito immune and other physiological responses to the invading ookinetes and other components of infected blood, and accurate execution of *Plasmodium’s* gene expression program that directs its developmental transitions and interactions with the vector. Major obstacles are encountered in the midgut tissue, where most parasites are killed by the mosquito’s immune system. Understanding the molecular interactions taking place between the malaria parasite and mosquito vector is essential for the development of malaria control strategies based o *Plasmodium* blocking in the mosquito. So far, most studies have focused on the rodent parasite model, *Plasmodium berghei*, which is more amenable to experimental procedures. A comparative analysis of *A. gambiae* transcript responses to midgut invasion of *P. berghei* and *P. falciparum* ookinetes showed broad variations and have identified factors that can modulate infection levels of both or only one of the two parasite species. Invasion by *P. berghei* had a more profound impact on the mosquito transcriptome, including a variety of functional gene classes, while *P. falciparum* elicited a broader immune response at the gene transcript level. Ingestion of human malaria-infected blood lacking invasive ookinetes also induced a variety of immune genes, including several anti-*Plasmodium* factors. Seven of 12 tested genes were found to influence mosquito resistance to both parasite species. An MD2-like receptor, AgMDL1, and an immunolectin, FBN39, showed specificity in regulating only resistance to *P. falciparum,* while the antimicrobial peptide Gambicin and a novel putative short secreted peptide, IRSP5, were more specific for defense against the rodent parasite *P. berghei*. While all the genes that affected *Plasmodium* development also influenced mosquito resistance to bacterial infection, four of the antimicrobial genes had no effect on *Plasmodium* development. The defense against the two *Plasmodium* species is mediated by antimicrobial factors with both universal and *Plasmodium*-species specific activities. The mosquito is capable of sensing infected blood constituents in the absence of invading ookinetes, thereby inducing anti-*Plasmodium* immune responses.

### angaGEDUCI: *Anopheles gambiae* gene expression database with integrated comparative algorithms for identifying conserved DNA motifs in promoter sequences

Sumudu Dissanayake^1^, Osvaldo Marinotti^1^, Jose Marcos C. Ribeiro^2^ and Anthony A. James^1,3^

^1^Department of Molecular Biology and Biochemistry, University of California, Irvine, CA 92697. Correspondence: omarinot@uci.edu

^2^Laboratory of Malaria and Vector Research (NIH/NIAID), Rockville, MD 20852

^3^Department of Microbiology and Molecular Genetics, University of California, Irvine, CA 2697

The completed sequence of the *Anopheles gambiae* genome has enabled genome-wide analyses of gene expression and regulation in this principal vector of human malaria. These investigations have created a demand for efficient methods of cataloguing and analyzing the large quantities of data that have been subsequently produced. The organization of genome-wide data into one unified database makes possible the efficient identification of spatial and temporal patterns of gene expression, and by pairing these findings with comparative algorithms, may offer a tool to gain insight into the molecular mechanisms that facilitate such expression patterns. We developed a publicly-accessible database and integrated data-mining tool, angaGEDUCI, that unifies 1) stage- and tissue-specific microarray analyses of gene expression in *An. gambiae* at different developmental stages, and temporal separations following a bloodmeal, 2) functional gene annotation, and 3) promoter sequence comparison algorithms. The database can be used to study genes expressed in particular stages, tissues, and patterns of interest, and to identify conserved promoter sequence motifs that may play a role in the regulation of such expression. The database is accessible from the address http://www.angaged.bio.uci.edu. The combination of gene expression, function, and sequence data in the angaGEDUCI database streamlines spatial and temporal pattern-finding and produces a straightforward means of developing predictions and designing experiments to assess how gene expression may be controlled at the molecular level.

### Caste-based differences in gene expression in the polyembryonic wasp *Copidosoma floridanum*

David M. Donnell and Michael R. Strand

Department of Entomology, University of Georgia, Athens, GA 30602 USA. Correspondence: donnell@bugs.ent.uga.edu

The polyembryonic parasitoid *Copidosoma floridanum* produces two morphologically and behaviorally distinct larval castes, soldiers and reproductives, during development within its host. Soldier larvae defend the brood against competitors while reproductive larvae develop into adult wasps. In this study we used a bi-directional suppression subtractive hybridization (SSH) approach to isolate differentially expressed genes of the two larval castes of *C. floridanum*. We isolated 230 novel expressed sequence tags (ESTs) from the two subtractions (114 soldier / 116 reproductive ESTs). Among these ESTs were sequences with significant similarity to serine proteinases, proteinase inhibitors, odorant and chemosensory binding proteins, and cuticular proteins. RT-PCR analysis of ESTs from each of these categories indicated that 85% were differentially expressed in one caste or the other. We conclude that our SSH strategy was effective in identifying a number of genes differentially expressed in each of the larval castes and suggest several of these differentially expressed genes will be useful in characterizing caste-specific gene networks in *C. floridanum*.

### The cuticular protein genes of *Anopheles gambiae* and *Apis mellifera*: Annotation and insights

W.A. Dunn, T. Togawa, J.H. Willis

Dept. of Cellular Biology, University of Georgia, Athens, GA 30602, USA. Correspondence: wadunn83@mac.com

Sequence and electrophoretic analyses revealed that the cuticle has many distinct cuticular proteins (CP), with the majority sharing a conserved sequence, the Rebers and Riddiford (RR) Consensus that has chitin-binding capability. We have annotated the CP genes in the RR family in *Anopheles gambiae*, using the conserved consensus as our guide and the genomic sequence data from the PEST strain. Over 130 RR genes have been identified, indicating that *Anopheles* devotes close to 1% of its genes to coding for this one class of cuticular proteins. Almost three quarters of the RR genes were found in just 5 clusters ranging in size from 9–35 members, with both plus and minus orientations. Within each cluster there may be several genes with virtually identical coding sequences, but with different 5′ and 3′ UTRs. Nearest neighbor analyses of all the RR-2 proteins revealed that sequences within a cluster formed distinct groupings; this was not found with the RR-1 proteins that are more divergent outside the strict consensus. In contrast with *A. gambiae*, only 28 members of the RR family were identified in *Apis mellifera*. The biological questions posed by these findings are provocative. At the sequence level alone, important questions abound about the origin of multigene families and the roles of gene conversion and purifying selection. Speculation is tempered by concerns about the accuracy of the *Anopheles* genome assembly. In addition to the RR family, *Anopheles* also has four members of each of two far smaller families, CPF and CPTC as well as another moderate size family that codes for CP with low sequence complexity caused by a high proportion of glycine or alanine residues.

### The ligand-bond X-ray structure of *Aedes aegpti* sterol carrier protein-2 like-2 at 1.7-angstrom resolution

David H. Dyer^1^, Katrina Forest^1^, Que Lan^2^

^1^Department of Bacteriology, University of Wisconsin-Madison, Madison, WI 53706 USA. Correspondence: dyer_dave@hotmail.com

^2^Department of Entomology, University of Wisconsin-Madison, Madison, WI 53706 USA

Sterol carrier protein-2-like-2 (SCP-2-L2) gene from the yellow fever mosquito, *Aedes aegypti*, is a member of the SCP2 gene family. The protein fold of SCP2-L2 is very similar to other members of the SCP2 structural family. However in the SCP-2-L2 structure, along with a similar position for the previously seen fatty acid in SCP-2, the formation of a dimer occurs revealing a large internal cavity spanning the two monomers. Within the cavity is a bound fatty acid which is in an orientation similar to the Triton X-100 seen in the human SCP2 domain from the peroxisomal multifunctional enzyme.

### Analysis of *Aedes aegypti* mosquito vitelline envelope gene promoters

Marten J. Edwards

Muhlenberg College, Biology Department, 2400 Chew Street, Allentown, PA 18104, USA. Correspondence: mailto: edwards@muhlenberg.edu

Three ecdysone-responsive *Ae. aegypti* vitelline envelope genes (15a-1, 15a-2 and 15a-3) are strongly expressed in the follicular epithelium of the ovaries following a blood meal. To assess whether vitelline envelope gene promoters can drive ectopic gene expression in transgenic *Ae. aegypti* mosquitoes, ~2.0 kb sequences upstream of the three genes were linked to a red fluorescent protein (RFP) reporter gene and inserted into a piggyBac transformation vector (3XP3-EGFP). These construct are being microinjected into *Ae. aegypti* embryos. *D. melanogaster* were transformed with the 15a-2.RFP.pBac construct. In four independent transgenic lines, ovarian RFP expression was not observed. Transformation of D. *melanogaster* with 15a-1.RFP and 15a-3.RFP piggyBac constructs is in progress. The three upstream sequences have also been linked to a human antiviral gene (MxA) and inserted into the piggyBac transformation vector. Driving MxA expression in the follicular epithelium of mosquitoes following a blood meal may allow us to assess whether infection of the follicular epithelium is a pre-requisite for the transovarial transmission of LaCrosse virus in *Oc. triseriatus* mosquitoes.

### RNAi suppression of recognition protein mediated immune responses in the tobacco hornworm *Manduca sexta* causes increased susceptibility to the insect pathogen *Photorhabdus*

I. Eleftherianos, P.J. Millichap, R.H. ffrench-Constant and S.E. Reynolds

Department of Biology and Biochemistry, University of Bath, Bath BA2 7AY, UK. Correspondence: bssie@bath.ac.uk

Bacterial pathogens either hide from or overcome the immune response of their hosts. Here we show that two different species of insect pathogenic bacteria, *Photorhabdus luminescens* TT01 and *Photorhabdus asymbiotica* ATCC43949 were both recognised by the immune system of its host *Manduca sexta*, as indicated by a rapid increase in the levels of mRNAs encoding three different inducible microbial recognition proteins, Hemolin, Immulectin-2 and Peptidoglycan Recognition Protein. RNAi mediated inhibition of expression (“knock-down”) of each of these genes at the level of both mRNA and protein was achieved through injection of double-stranded RNA (dsRNA). Knock-down of any one of these genes markedly decreased the ability of the insects to withstand infection when exposed to either species of *Photorhabdus*, as measured by the rate at which infected insects died. RNAi against immulectin-2 caused the greatest reduction in host resistance to infection. The decreased resistance to infection was associated with reduced hemolymph phenoloxidase activity. These results show not only that *Photorhabdus* is recognised by the *M. sexta* immune system but also that the insect’s immune system plays an active, but ultimately ineffective, role in countering infection.

### A gene operon that enables the insect-pathogenic bacterium *Photorhabdus asymbiotica* to survive within phagocytic hemocytes of the insect *Manduca sexta*

I. Eleftherianos, N.R. Waterfield, R.H. ffrench-Constant, and S.E. Reynolds

Department of Biology and Biochemistry, University of Bath, Bath BA2 7AY, UK. Correspondence: bssie@bath.ac.uk

*Photorhabdus* bacteria are lethal pathogens of insects. Like all pathogens, the bacteria must evade or overcome host immune defences in order to survive and proliferate. *Photorhabdus* produces several lethal toxins (eg Tc toxins and Mcf-1), but other virulence genes are likely to contribute to pathogenesis by enabling *Photorhabdus* to persist and multiply within the insect until bacteria are present in sufficient numbers to be able to kill the host. Here we describe a screen of a fully sequenced *Photorhabdus* genome that aimed to discover such persistence genes. We screened cosmids to find those that allowed a cloning strain of *E. coli* to persist within and ultimately kill *Manduca sexta* caterpillars. In this way we identified a *Photorhabdus* operon *kdp* that encodes the protein subunits of a bacterial K^+^ ion transporter and the two-component regulator that governs their expression. Expression of these *Photorhabdus* genes allows *E. coli* to persist within phagocytic hemocytes; disruption of any one of the operon’s genes prevents persistence. Since expression of the two-component regulator genes alone is sufficient for persistence, we conjectured that the distinctive feature of *Photorhabdus* kdp is the sensor-regulator pair, which can also regulate expression of the *E. coli* kdp genes to the detriment of the host insect. We confirmed this hypothesis by showing that whereas *E. coli* kdp genes are not expressed after phagocytosis, the kdp genes of *Photorhabdus* are strongly expressed within *Manduca* phagocytes.

### Genes for honey bee heat shock proteins: Description and comparison

Michelle M. Elekonich

Department of Biological Sciences, University of Nevada Las Vegas, 4505 Maryland Parkway, Las Vegas, Nevada 89154, USA. Correspondence: michelle.elekonich@unlv.edu

Hsps and their encoding genes (*hsps*) are nearly universal in organisms, highly-conserved, and assigned to families on the basis of sequence homology and typical molecular weight. Members of several Hsp families differ in inducibility by stressors, intracellular localization, and function. Hsps interact with other proteins that are in non-native conformations (whether due to protein-denaturing stress or because the peptides they comprise are not fully mature) to promote refolding, minimize their aggregation or target them for degradation and removal from the cell. As part of a larger annotation effort following the sequencing of the honey bee genome, we identified heat shock protein genes from the hsp70, hsp90, and hsp 40 families. Despite being endothermic insects and exhibiting extreme heat tolerance (up to 50°C for 1 hour) there has not been a large increase in the number honey bee *hsp70*s compared to those in other insect genomes. Comparisons between the honey bee, fly, and mosquito sequences suggest that the fly model may not represent the ancestral situation.

### A stage-specific ovarian factor with stable stimulation of juvenile hormone synthesis by corpora allata of the cockroach *Diploptera punctata*

K.L. Elliott, A.P. Woodhead, B. Stay

Department of Biological Sciences, University of Iowa, Iowa City, IA 52242, USA. Correspondence: mailto: barbara-stay@uiowa.edu

*In vivo* studies of the cockroach *Diploptera punctata* have shown stage-specific ovarian stimulation of juvenile hormone (JH) synthesis by corpora allata (CA) (Rankin and Stay, 1984. Gen. and Comp. Endocrinology. 54: 382–388). Using ovary-conditioned medium (OCM) to treat CA *in vitro*, a non-stage-specific stimulatory factor was found to be released by all stages of ovaries in the 8 days of the first ovarian cycle and this factor was recovered in the flow-through after solid-phase extraction of the OCM (Unnithan et al., 1998. J. Insect Physiol. 44: 1027–1037). The present study provides evidence for a different ovarian factor that stimulates JH synthesis and is stage-specific. The stage-specific factor was found by conditioning medium with ovaries from different stages in the reproductive cycle (pre-vitellogenic, day 1; rapid vitellogenesis, days 2–4; and post-oviposition, day 8). One member of a pair of CA from day 3 mated females was treated with OCM and the other with the flow-through of that OCM after solid-phase extraction on a C_18_ reverse phase Sep-Pak cartridge. CA were then transferred to new medium and rates of JH synthesis were measured by an *in vitro* radiochemical assay. Only CA conditioned with day 2 and 3 OCM had significantly higher rates of JH synthesis than CA conditioned with flow-through. The CA responded to this factor in a dose-dependent manner and the factor was shown to be sensitive to trypsin but not to freezing. These results indicate that the increasing rates of JH synthesis that accompany rapid growth of the basal ooctyes of the ovary results from the release of a stage-specific peptidergic ovarian factor that acts directly on the CA to induce a stable stimulation of JH synthesis.

### Distribution of ionotropic (RDL) and metabotropic (GABA_B_) receptors for GABA in the brain of *Drosophila*

L. E. Enell, Y. Hamasaka, D.R. Nässel

Department of Zoology, Stockholm University, SE10691 Stockholm, Sweden. Correspondence: lina.enell@zoologi.su.se

Gamma-aminobutyric acid (GABA) is the major inhibitory neurotransmitter in insects, including *Drosophila*. GABA is produced in a large number of interneurons throughout the CNS of *Drosophila*; no GABAergic sensory or motorneurons have been detected. Two major types of GABA receptors are known: (1) ligand-gated ion channel-type GABA_A_ receptors formed as multimers of different subunits and (2) metabotropic GABA_B_ receptors (GABA_B_Rs) which are G-protein-coupled receptors (GPCRs). The Drosophila GABA_B_R1 and R2 subunits form functional heterodimers. We raised specific antisera to the GABA_B_R2 and to the GABA_A_R subunit RDL (Resistance to Dieldrin) of Drosophila to map receptor distribution in the CNS. The receptor distribution was compared to that of GABAergic neurons identified by immunocytochemistry or by driving green fluorescent protein in GAL4 lines specific for GABA signaling or specific interneurons. We find an abundant, but selective, distribution of both RDL and GABA_B_R2 immunoreactivity in the brain. The two types of receptors display similar general distribution in some, but not all, neuropil areas. Most prominently labeled with antisera to both receptors were neuropils of the antennal lobes, optic lobes and the calyces of the mushroom bodies. Here, we especially investigated the antennal lobes. There is a close match between distribution of GABA and the two receptor types. The difference in detailed distribution of GABA_A_ and GABA_B_ receptors may reflect their postulated functional properties: GABA_A_ units form a postsynaptic receptor mediating fast inhibition and GABA_B_ units form pre- and/or postsynaptic receptors mediating slow inhibition. Supported by the Swedish Research Council and the Swedish Animal Welfare Agency.

### The metamorphosis-regulators, E75 and Broad play conserved and divergent roles in the direct-developing milkweed bug, *Oncopeltus fasciatus*

Erezyilmaz, D.F., Kelstrup, H., Truman, J.W. and Riddiford, L.M.

Department of Biology, University of Washington, Box 351800, Seattle, WA. USA. Correspondence: denizere@u.washington.edu

The transcription factors Broad (Br*)* and E75A play well-established roles in regulating molting and metamorphosis in holometabolous insects. *br* is required for the larval-pupal transition, while E75A acts to couple the molting cycle with metamorphosis. To shed light upon the genetic basis for the evolution of complete metamorphosis, we have examined the function of these two transcription factors in the milkweed bug, *Oncopeltus fasciatus*. We find that the postembryonic roles of the two genes are comparable with their functions during metamorphosis of holometabolous insects. During the nymphal stages, E75A is required for molting, as E75A dsRNA-injected nymphs fail to molt to the next stage. Unlike its postembryonic expression during holometabolous development, where *br* is restricted to the larval-pupal transition, we find that *br* is expressed at each nymphal molt, but not at the molt to the adult. Injection of *br* RNAi into nymphs led to a repeat of the stage at the next molt. This stasis involved both the pigmentation pattern and the wing pad proportions that normally characterize a given stage. In contrast to their postembryonic roles, the effects of *E75* and *br* kock-down were entirely unexpected. We find that *br* is expressed during segmentation, which occurs in *Oncopeltus* in the context of germ band invagination. Loss of *br* through maternal RNAi results in posterior truncations. Interestingly, E75 also plays a role in segmentation. In this case, however, loss of E75 results in loss of the labial, T2 and T3 segments through fusion with more posterior segments. In addition, loss of abdominal segments may occur through fusion. We are currently trying to determine the relationship between these transcription factors and canonical patterning genes in early embryonic development. This work was supported by NIH RO1GM-10166.

### Pathway and transcriptional insights into honey bee immunity from the Honey Bee Genome Project

J.D. Evans, and the Honey Bee Genome Sequencing Consortium

Bee Research Laboratory, USDA-ARS Beltsville, MD 20705. Correspondence: evansj@ba.ars.usda.gov

Honey bees combat disease through both social, or group-level, mechanisms and individual defenses. Bees face significant parasites and pathogens across many taxonomic groups, including bacteria, viruses, protists, and fungi, and a wealth of pathology data for honey bees can be used to explore host-parasite interactions. Like other insects, honey bees use components of the innate immune response to defend against pathogens, and recent work has explored the mechanisms^1^ and efficacy^2^ of this response. With the sequencing, assembly, and annotation of the honey bee genome it is possible to propose and test hypothetical immune-pathway models for bees. Honey bees show likely orthologs for nearly all members of the canonical insect innate immune pathways (Toll, Imd, JNK, and Jak/STAT). Surprisingly, honey bees have sharply lower immune-pathway redundancy when compared to other insects, implying decreased flexibility in the immune responses of bees toward pathogens. Of 17 immune gene families implicated in recognition, signaling, and effecting an immune response, bees have approximately 1/3 the gene diversity found in the *Drosophila melanogaster* and *Anopheles gambiae* genomes. This reduction could reflect the protective strength of behavioral and environmental barriers to honey bee disease, a tendency of bees to be attacked by a limited set of coevolved pathogens, or novel, parallel mechanisms with which bees achieve immune response flexibility. Transcript abundance levels for pathway members and effectors are presented in order to describe the covariance and heritability of immune responses in bees. ^1^K. Aronstein, E. Saldivar, *Apidologie* 36, 3–14 (2005), ^2^J. D. Evans, J. S. Pettis, *Evolution* 59, 2270–2274 (2005)

### Bt toxin binding domains in pink bollworm (*Pectinophora gossypiella*) cadherin

Jeff A. Fabrick^1^ and Bruce E. Tabashnik^2^

^1^USDA-ARS, U.S. Arid Land Agricultural Research Center, 21881 N. Cardon Lane, Maricopa, AZ 85239, USA. Correspondence: jfabrick@wurl.ars.usda.gov

^2^Department of Entomology, University of Arizona, Tucson, AZ 85721, USA.

Transgenic crops producing toxins from *Bacillus thuringiensis* (Bt) are widely used for pest control, including cotton that produces Bt toxin Cry1Ac and kills key lepidopteran pests. Both cadherin and aminopeptidase have been implicated as Bt toxin receptors. Although field-evolved resistance to Bt crops has not yet occurred, laboratory selection results show that many pests can evolve resistance to Bt toxins. The most common mechanism of resistance is reduced binding of toxin to midgut receptors. Resistance to Cry1Ac in several lab-selected strains of the global cotton pest, pink bollworm (*Pectinophora gossypiella*), is tightly linked to a cadherin gene (Morin *et al.*, 2003. PNAS 100, 5004–5009 and Tabashnik *et al*., 2005. J. Econ. Entomol. 98, 635–644). We report that Cry1Ac binds to recombinant peptides corresponding to extracellular regions of the pink bollworm cadherin (BtR). Similar to other lepidopteran cadherin receptors, pink bollworm BtR has at least two binding domains, each adjacent to the membrane proximal region. However, unlike cadherins from *Manduca sexta* and *Bombyx mori*, toxin binding was not observed in regions more distally located from the membrane proximal region. We also report that both the protoxin and activated toxin forms of Cry1Ac bound to recombinant pink bollworm BtR fragments, suggesting that Cry1Ac activation may occur either before or after receptor binding. The results support the hypothesis that cadherin is a receptor for Cry1Ac in pink bollworm.

### Effects of *Wolbachia* infection on metabolic processes in cultured mosquito cells

Ann M Fallon

Department of Entomology, University of Minnesota, 1980 Folwell Ave, St. Paul, MN 55108. Correspondence fallo002@umn.edu

In mosquitoes, the intracellular bacterium known as *Wolbachia pipientis* causes a reproductive distortion known as cytoplasmic incompatibility, which favors production of progeny by infected females. Because the infection is transmitted to mosquito offspring, *Wolbachia* facilitates its own spread through uninfected populations. Mathematical models indicate that *Wolbachia* provides a potentially effective agent for introducing, into vector populations, transgenes designed to reduce disease transmission. In the laboratory, *Wolbachia* can be transferred between insect species, and can be introduced into cultured cells. Alternatively, infected cell lines, such as the *Aedes albopictus* Aa23 line (O’Neill et al., 1997; Insect Molecular Biology 6, 33–39), can be derived from infected insect embryos. We are standardizing the in vitro production of *Wolbachia* in *Aedes albopictus* mosquito cell lines by comparing infected Aa23 cells to the uninfected C7-10 *Aedes albopictus* cell line, and to C7-10 cells infected with *Wolbachia* from Aa23 cells. *Wolbachia*-infected cells grow more slowly than uninfected cultures, have reduced ability to incorporate tritiated thymidine into DNA, and undergo apoptosis as the abundance of *Wolbachia* increases. In Aa23 cells, the secretion of immune-induced proteins into the cell culture medium is substantially reduced, relative to that in C7-10 cells. We hypothesize that in infected cell cultures, modulation of the immune response contributes to *Wolbachia* survival and replication.

### Proteomic approach to investigate aphid - plant interactions

Frédéric Francis^1^, Nicolas Harmel^1^, Edwin De Pauw^2^ and Eric Haubruge^1^

^1^ Functional and Evolutionary Entomology, Gembloux Agricultural University, Passage des Deportes 2, 5030 Gembloux, Belgium. Correspondence: francis.f@fsagx.ac.be

^2^Mass Spectrometry Laboratory, University of Liège, BAT. B6 Chimie physique, allee de la Chimie 3, 4000 Liege 1, Belgium

Plant-insect relations are mainly regulated by the evolution of the defense mechanisms developed by plants and the ways herbivore insects adapt themselves to these defensive systems. Plant defense can be direct or indirect, localised or systemic. A common property of these mechanisms is the broad range of phytophagous agents, including insect pests, which are efficiently controlled by the defensive produced molecules. To cope with the induction of several direct defense molecules, herbivores developed several detoxification enzymatic systems such as the gluthathione S-transferases and monooxygenases. Here we studied the chemical ecology of aphid (such as *M. persicae*)–plant relations using a proteomic approach. The aphid switch from one host plant to others within the Solanaceae and Brassicaceae family was first investigated to assess the metabolic changes and potential adaptations in aphids according to particular host plant species. Specific associations between aphids and their host plants were previously shown to be related to the presence of particular bacterial symbionts. The respective role of the aphid and their related symbionts in the adaptation to the host plant was also investigated considering the proteome variations of aphids in presence or absence of endosymbionts. Finally, the particular role of aphids in plant defensive responses due to its sucking feeding behavior was investigated focusing on the protein composition of aphid saliva. The complex protein mixtures from different aphid materials were separated by two dimension electrophoresis methods and the related spots of proteins significantly varying were selected and identified by mass spectrometry (ESI-MS-MS and Maldi-Tof-MS-MS) coupled with data bank investigations. The impact of the down regulated or over expressed aphid proteins involved in different metabolic pathways was discussed. This broad proteomic approach is a very reliable tool to study the biologically involved proteins from aphids in response to several environmental changes, and particularly the insect-host plant interactions.

### Mapping of hemoglobin proteolysis in the hard tick *Ixodes ricinus*

Z. Franta^1^, M. Horn^2^, D.Sojka^1^, M. Mares^2^, and P. Kopácek^1^

^1^Institute of Parasitology, Biological Centre Academy of Sciences of the Czech Republic and Faculty of Biological Sciences University of South Bohemia, Ceské Budejovice. Correspondence: zdeny@paru.cas.cz

^2^Institute of Organic Chemistry ad Biochemistry, Academy of Sciences of the Czech Republic, Prague.

Ticks differ from other hemaptophagous parasites in the intracellular localization of hemoglobin proteolysis. Hemoglobin digestion in ticks is a critical process for two reasons: It provides primary energy resources, and the generated hemoglobin fragments function as antimicrobial peptides. Hemoglobin digestion in ticks is still poorly understood at the molecular level. We have analyzed the peptidase spectrum in the gut of the hard tick *Ixodes ricinus*, a vector of Lyme disease and tick-borne encephalitis. Substrate/inhibitor-based profiling demonstrated endo- and exopeptidases of cysteine and aspartic class in the tick gut homogenate. The screening of gut-specific cDNA by PCR amplification was performed with primers derived from the conserved regions of the detected peptidases. It resulted in identification of genes coding for (i) cysteine peptidases: asparaginyl endopeptidase (legumain), cathepsin B1, B2 and L, and dipeptidyl peptidase I (cathepsin C), and (ii) aspartic peptidase cathepsin D. Tissue expression analysis by RT PCR revealed that all peptidases (with exception of cathepsin L) are expressed specifically in the tick gut. Taken together, the proteolytic machinery in the tick gut closely resembles the digestive system of *Schistosoma* blood flukes but differs substantially from hematophagous insects relying mainly on serine peptidases. This work was supported by Grant Agency of the Czech Republic No. 206/06/0865, and research projects Nos. Z60220518, Z40550506 and MSMT 6007665801.

### Lipid metabolism in the hematophagous *Panstrongylus megistus*: Interaction lipophorin-midgut membrane

L.L. Fruttero^1^, R. Stariolo^2^, E.R. Rubiolo^1^ and L.E. Canavoso^1^

^1^Departamento de Bioquímica Clínica, CIBICI-Conicet. Facultad de Ciencias Químicas, Universidad Nacional de Córdoba. Córdoba, Argentina. Correspondence: lcanavo@mail.fcq.unc.edu.ar

^2^Coordinacion Nacional de Control de Vectores. Córdoba, Argentina, CP 5000

In insects, the transfer of midgut lipids to the hemolymph is a remarkable event mediated by lipophorin (Lp), the main insect lipoprotein. In order to understand the regulation of this process in hematophagous insects we have analyzed the transfer of diacylglycerol into circulation, and the interaction Lp with the midgut membrane using a solid-phase binding assay. In addition, the sites of interaction of Lp with the midgut cells were localized by immunofluorescence assays. This study was performed employing *Panstrongylus megistus* (Hemiptera:Reduviidae), an important vector of Chagas’ disease. This insect takes large blood meals, containing a substantial amount of lipid. Lp was isolated from hemolymph of fifth instar nymphs by a KBr gradient and the membranes were obtained by ultracentrifugation of midgut homogenates. Thereafter, the membranes were suspended by mild sonification, adsorbed in plates, and the amount of bound Lp was quantified by ELISA. The factors analyzed included the effect of ionic strength on binding, the effect of pH, the requirement of divalent cations, and the effect of suramin. The saturation kinetics most likely fit a ligand-binding model for a single binding site. The interaction between Lp and the membrane showed a strong dependence with the pH and, in contrast with LDL receptor family, did not required Ca^+2^ or Mg^+2^. Like other lipoprotein receptors, suramin significantly inhibited the interaction between Lp and the membrane. The effect of ionic strength suggested that Lp binding is optimal at low NaCl concentration. In addition, the partial effect on binding after membranes were treated with proteases or changes in temperature strongly indicated that such interaction needs proteins in addition to other components of the membrane. Finally, immunofluorescence assays showed that the interaction of Lp with midgut cells mainly occurred at the basolateral region of the membrane cells. *Supported by Secyt-UNC (E.R.R) and CONICET-Argentina (L.E.C). L.L. Fruttero is a research fellow from CONICET-Argentina.*

### Role of the transcriptional repressor *mdGfi-1* in *CYP6D1v1*-mediated insecticide resistance in the house fly, *Musca domestica*

Jianwei Gao, Jeffrey G. Scott

Department of Entomology, Comstock Hall, Cornell University, Ithaca, NY 14853-0901, USA. Correspondence: jgs5@cornell.edu

Gfi-1 is a C_2_H_2_-type zinc finger protein that is a transcriptional repressor in vertebrates and has been implicated in control of *CYP6D1* expression in house flies (*Musca domestica*). A 15 bp insert which disrupts a putative mdGfi-1 binding site in the *CYP6D1v1* promoter has been implicated as a cause of increased expression of CYP6D1, and thus insecticide resistance. Using electrophoretic mobility shift assays we demonstrate that the CYP6D1 promoter from susceptible strains binds mdGfi-1. The 15 bp insert that interrupts the mdGfi-1 binding site in insecticide resistant strains reduces the amount of mdGfi-1 binding by 9- to 20-fold, consistent with the role of *mdGfi-1* in resistance. Partial sequences of *mdGfi-1* (spanning the first intron) from individual house flies from 11 different strains revealed the presence of 23 alleles. There was no consistent difference in the *mdGfi-1* alleles between susceptible and CYP6D1-mediated insecticide resistant strains, indicating that *mdGfi-1* alleles were not likely involved in resistance. Polymorphisms were used to map *mdGfi-1* to autosome 1. Quantitative real time PCR (qRT-PCR) revealed Gfi-1 expression was higher in the thorax compared to the head and abdomen, and varied between life stages and between strains. However, similar levels of mdGfi-1 were detected in susceptible and resistant adults suggesting that altered levels of mdGfi-1 were not likely a cause of insecticide resistance. The significance of these results to understanding insecticide resistance is discussed.

### Molecular mechanisms of resistance to *Bacillus thuringiensis* and variation in the magnitude of fitness costs

Aaron J. Gassmann, Yves Carrière, and Bruce E. Tabashnik

Department of Entomology, The University of Arizona, Tucson, AZ, 85721. Correspondence: gassman@ag.arizona.edu

During the past decade, 35 studies have tested for fitness costs associated with resistance to the insecticidal toxins produced by the bacterium *Bacillus thuringiensis*. These studies demonstrate a high level of variation in the extent to which resistance to Bt is associated with fitness trade-offs. More recently, the molecular basis of resistance has come to light for several insect species. Although resistance is often associated with mutations at a caderhin locus, alternative resistance mechanisms exist. We review the literature on fitness costs of resistance to Bt and on the molecular basis of resistance. We consider whether differences in the magnitude or manifestation of fitness costs arise between insect species with contrasting molecular mechanisms of resistance, and whether the fitness costs of resistance differ among insect species that have cadherin-based resistance to Bt.

### The regulation by iron of a putative divalent metal transporter and ferritin in *Anopheles gambiae* 4a3b cells

D. L. Geiser^1^, J. S. D. Bridgers^2^ and J. J. Winzerling^1^

^1^Nutritional Sciences, The University of Arizona, 1177 E. 4^th^ St., Shantz Bldg. Rm. 405, Tucson, AZ, USA. Correspondence: dlgeiser@email.arizona.edu

^2^Biochemistry and Molecular Biophysics, The University of Arizona, Tucson, AZ, USA

Forty-one percent of the world’s population lives in areas where malaria is transmitted and an estimated 700,000-2.7 million persons die of malaria each year. Female mosquitoes such as *Anopheles gambiae* (African malaria mosquito, Diptera) require a blood meal for oogenesis (egg laying). It is during blood-feeding that these mosquitoes transmit diseases and receive a potentially toxic level of iron in both heme and non-heme forms. Heme is incorporated into the peritrophic matrix in the midgut of *Aedes aegypti* (yellow fever mosquito) during digestion and is potentially the primary mechanism of heme detoxification. It is not clear how non-heme iron is transported. In mammals, the divalent metal transporter, DMT1 (NRAMP2/ SLC11A2), is the primary importer of dietary non-heme iron into intestinal epithelial cells. DMT1 also transports iron from endosomes into the cytoplasm in somatic cells. We previously demonstrated that cultured *A. aegypti* larval cells will take up iron from culture medium by as yet unknown mechanisms. Iron inductively coupled plasma-mass spectrometry (ICP-MS) shows a dose dependent increase in iron concentration in the cytoplasm and membrane extracts of iron treated cells. Mosquito ferritin is the chief iron storage protein for these animals which is composed of heavy chain (HCH) and light chain (LCH) subunits that are homologues of the vertebrate ferritin subunits. Electromobility shift assays for iron regulatory protein 1 (IRP1) binding activity and immunoblot analysis for ferritin also supports the entry of iron into these mosquito cells. As part of a pilot study, we identified a putative DMT1 (pDMT1) from the *A. gambiae* protein database (NCBI). This pDMT1 was amplified, cloned and sequenced from *A. gambiae* larval cells, MOS55. Our preliminary experiments demonstrated that pDMT1 message in MOS55 cells has a biphasic response to increasing concentrations of ferric ammonium citrate (FAC). Recently, we successfully obtained the cDNA sequences for *A. gambiae* HCH and LCH. We have evaluated the mRNA expression of pDMT1, HCH, LCH as well as IRP1 under varying concentrations of iron excess, iron deprivation and a ferric rescue in *A. gambiae* hemocyte-like cells, 4a3b. Ultimately, studies will be conducted to determine the importance of these proteins in iron transport and storage by co-immunofluorescence, EM and ICP-MS.

### Activation of the EGF receptor influences extension and fasciculation of receptor axons in the developing olfactory system of the moth *Manduca sexta*

N.J.Gibson and L.P.Tolbert

Arizona Research Laboratories, Division of Neurobiology, University of Arizona, Tucson, AZ, USA. Correspondence: njgibson@neurobio.arizona.edu

Developing olfactory systems provide models for exploring mechanisms that influence axons as they seek their targets. During development of the adult antennal (olfactory) system of *Manduca sexta*, axons of olfactory receptor neurons (ORNs) interact with each other and with glial cells in a specialized "sorting zone" (SZ) as they sort by odor specificity. Once sorted, they seek the correct target sites in the antennal lobe (AL) of the brain and terminate in protoglomeruli. We reported previously that as they extend through the SZ and form protoglomeruli, ORN axons can be labeled with antibodies against human EGF receptors (EGFRs), and that treatment with the EGFR kinase blocker PD168393 results in failure of the axons to extend and sort properly in the SZ and in the nerve layer. We also reported that neuroglian, an IgCAM that can activate EGFRs through homophilic interactions, is present transiently on ORN axons and glia in the SZ and AL during the same period. Now we know that 9 of 10 amino acids through which PD168393 binds to human EGFR are conserved in insects. Blast analysis of *Manduca* ESTs indicates 67% homology to the peptide sequence used to produce the anti-human EGFR antibodies, and blocking with the corresponding moth peptide eliminates labeling. We have used Triton extraction tests to determine whether neuroglian binding may be occurring in the SZ, as a prerequisite to potentially activating EGFRs. We find that neuroglian is resistant to Triton extraction, indicating attachment to the cytoskeleton in response to homo- or heterophilic binding, on ORN axons only in the SZ and in the nerve layer of the AL. This supports the hypothesis that homophilic interactions between neuroglian molecules in these regions causes activation of EGFRs, which is necessary for further axonal extension and for sorting. In addition to blocking activation of EGFRs, PD168393 treatment results in a reduction of immunolabeling for neuroglian on ORN axons, suggesting that EGFR function and neuroglian expression are coupled. Support Contributed By: NIH DC004598

### Structural and functional characterization of a *Campoletis sonorensis* ichnovirus (CsIV) Cys-motif protein (WHv1.6)

Torrence A. Gill, and Bruce A Webb

Department of Entomology, University of Kentucky. Correspondence: gilltorrence@hotmail.com

Campoletis sonorensis is an endoparasitoid that of lepidopteran larvae. C. sonorensis has a symbiotic mutualism with a polydnavirus (CsIV) which is integrated in the genome of the wasp. During oviposition of endoparasitoid egg into the lepidopteran host, the wasp also injects venoms, ovarian proteins and CsIV. The ovarian and venom proteins transiently inhibit lepidopteran immune response over the initial 24 hours. Viral protein titers become high enough over this period to induce pathologic effects on the host, notably the viral proteins suppress cellular and humoral immunity, arrest host development, and suppress synthesis of some host proteins. CsIV has a double stranded, segmented DNA genome, that contains multiple segments present in different molar ratios. The CsIV genome also encodes multiple gene families. The CsIV cys-motif gene family has 10 family members, all of which appear to be expressed. Although some of these genes have previously been investigated the Whv1.6 gene is little studied. Here we report studies of this gene family member and show that is has the highest expression level. Whv1.6 is encoded on a hypermolar segment in the CsIV genome, Segment W, which is also a nested segment. To study the function of the encoded protein, the Whv1.6 gene was cloned into a baculovirus expression vector and the expressed protein purified. The purified protein was shown to inhibit insect growth when fed to larvae. The protein was then used to produce an antibody with the protein detected in parasitized plasma 6 hours post-parasitization, and throughout infection the normal period of parasitization. WHv1.6 was detected by Western blots in several host tissues, notably fat body and hemocytes, at two and seven days post infection, and this finding was corraborated by immunofluorescence detection assays. These functional studies suggest that this protein is involved in suppression of host immune and developmental systems in parasitized larvae.

### Endocrine regulation of pheromone production in the pinyon Ips, *Ips confusus*

Matthew D. Ginzel^1^. Christopher I. Keeling^1’2^, Claus Tittiger^1^ and Gary J. Blomquist^1^

^1^Department of Biochemistry and Molecular Biology, University of Nevada, Reno, NV 89523, USA. Correspondence: ginzel@unr.edu

^2^Michael Smith Laboratories, University of British Columbia, Vancouver BC V6T 1Z4, CANADA

Bark beetles are among the most economically important forest pests in the northern hemisphere, and rely on monoterpenoid aggregation pheromones to coordinate host colonization and mating. In this study, we investigate the interplay between feeding on host phloem and the induction of de novo pheromone biosynthesis in the pinyon Ips, *Ips confuses* (Coleoptera: Scolytidae). *I. confusus* has become a major pest in the southwestern United States, destroying hundreds of thousands of acres of pinyon pines. Juvenile hormone (JH) III regulates pheromone production in a number of bark beetles. Interestingly, it appears that JH III alone does not stimulate pheromone biosynthesis in male/. *confusus,* but rather some other regulatory factor is required for pheromone production. We have found that feeding on host phloem, but not JH III treatment, strongly induces pheromone production in male/. *confusus.* In males, feeding also stimulates the activity of a number of mevalonate pathway enzymes including 3-hydroxy-3-methyl-glutaryl-CoA reductase (HMG-R), which is thought to be the most highly regulated enzyme in the pathway. Nevertheless, feeding and JH III both significantly up-regulate mRNA levels of *HMG-R and* other mevalonate pathway genes.

### Mechanisms of broad spectrum and R gene-mediated plant defenses against aphids

Fiona L. Goggin^1^, Stephanie L. Hebert^1^, and D.A. Navarre^2^

^1^Dept. of Entomology, University of Arkansas, Fayetteville, Arkansas. Correspondence: fgoggin@uark.edu

^2^USDA-ARS, Washington State University, Prosser, WA

The majority of studies on insect resistance have focused on plant defenses against chewing insects, such as caterpillars. Far less is known about resistance to piercing-sucking insects, such as aphids. We have compared transcript profiles induced in tomato (*Lycopersicon esculentum*) by the potato aphid (*Macrosiphum euphorbiae*) versus the beet armyworm (*Spodoptera exigua*), in order to understand plants’ differential responses to these two feeding guilds. Micorarray analysis identified 94 unigenes that were differentially regulated (induced or repressed) by aphid feeding, and 212 unigenes responsive to caterpillar feeding. The profile of genes regulated by the potato aphid showed only 25% overlap with transcript profiles induced by caterpillar feeding; thus, our data supports the hypothesis that plant responses to phloem-feeding insects are qualitatively different from plant defenses against chewing insects. We are also using tomato as a model system to investigate mechanisms of acquired and innate resistance against the potato aphid. Acquired resistance depends upon broad-spectrum systemic defenses that are induced by an initial pest infestation, and that render plants less susceptible to subsequent attack. We have utilized mutant tomato lines deficient in key defensive signaling pathways to explore the role of these pathways in acquired plant defenses against aphids. We have also explored innate resistance mechanisms that target specific pests, and limit their initial establishment on the plant. In tomato, innate resistance to the potato aphid depends upon a resistance (*R* ) gene called *Mi-1.2*. We are currently using microarrays to characterize gene expression profiles induced by aphid feeding in tomato lines with and without *Mi-1.2*, in order to investigate the mode of action of this gene.

### Neurogenomics of honey bee behavior

Christina M. Grozinger

Departments of Entomology and Genetics, W.M. Keck Center for Behavioral Biology North Carolina State University, Raleigh, NC 27695, USA. Correspondence: christina_grozinger@ncsu.edu

Honey bees have long been excellent models for behavioral studies, since they have complex yet highly malleable behavior. With the recent completion of the genome sequence and advent of microarray techniques, we can now hope to unravel the molecular mechanisms underlying these behaviors. We are using microarrays to characterize the gene expression patterns in the honey bee brain associated with two fundamental aspects of honey bee social life: responses to pheromones and changes in reproductive state. Here, I will summarize a recent microarray study comparing sterile and reproductive workers with virgins queens, focusing on the general expression patterns and types of genes associated with reproductive state and caste. Secondly, I will describe a series of studies on a gene whose brain expression levels were previously identified as being responsive to queen mandibular pheromone (QMP). Our new findings show that the regulation of the expression of this gene in the bee brain in response to QMP depends on the physiological and behavioral state of the bee. By both studying the function of these genes and using them as brain markers of behavioral changes, we can gain insight into the mechanisms underlying behavioral plasticity.

### Inhibitors of cysteine peptidases in the soft tick *Ornithodoros moubata*

L. Grunclová^1^, M. Horn^2^, M. Vancová^1^, Z. Franta^1^, M. Mares^2^, and P. Kopácek^1^

^1^Institute of Parasitology, Biological Centre Academy of Sciences of the Czech Republic and Faculty of Biological Sciences University of South Bohemia, Ceské Budejovice. Correspondence: kopajz@paru.cas.cz

^2^Institute of Organic Chemistry ad Biochemistry, Academy of Sciences of the Czech Republic, Prague.

Three genes coding for cysteine peptidase inhibitors were isolated from the gut-specific cDNA library from the soft tick *Ornithodoros moubata*; Om-cystatin 1 and 2, and Om-thyropin (containing one thyroglobulin domain). Om-cystatin 1 is mainly expressed in the tick gut, Om-cystatin 2 and thyropin messages were also found in other tick tissues. All three inhibitors were clearly down-regulated after a blood meal. Western blot analysis revealed that the native Om-cystatin 2 was significantly more abundant than Om-cystatin 1 and thyropin in the gut contents of fasting ticks. Om-cystatins were associated with the hemosome-derived residual bodies accumulated in the gut. Om-cystatin 2 was also found to be expressed by the type 2 secretory cells in the salivary glands of un-fed ticks. The inhibitory specificity of recombinant cystatins was screened against mammalian cysteine peptidases as well as endogenous cysteine peptidases present in the tick gut. Om-cystatins inhibited efficiently papain-like endopeptidases, differed significantly in their affinity towards dipeptidyl aminopeptidase I, and failed to block asparaginyl endopeptidase. The results suggest that the secreted cysteine peptidase inhibitors are involved in regulation of multiple proteolytic targets in the tick digestive system and tick-host interaction. This work was supported by Grant Agency of the Czech Republic No. 206/06/0865, and research projects Nos. Z60220518, Z40550506 and MSMT 6007665801.

### Distribution of elongation factor-1□ in larval tissues of the fall armyworm, *Spodoptera frugiperda*

J. Habibi^1^, C.L. Goodman^2^, and M.K. Stuart^3^

^1^Department of Internal Medicine, University of Missouri, Columbia, MO, USA

^2^U.S. Department of Agriculture, Agricultural Research Service, Biological Control of Insects Research Laboratory, Columbia, MO, USA

^3^Department of Microbiology/Immunology, Kirksville College of Osteopathic Medicine, A.T. Still University, Kirksville, MO 63501, USA. Correspondence: mstuart@atsu.edu

Elongation factor-1□ (EF-1□) promotes the delivery of aminoacyl-tRNA to the acceptor site of the ribosome during protein synthesis. It also plays a pivotal role in regulating apoptosis: cultured insect cells that accumulate EF-1□ become apoptotic, possibly by enhancing translation of “killer factors” such as caspase enzymes. Mab 7D6, a monoclonal antibody generated to EF-1□ from the fall armyworm, inhibits *in vitro* translation when added to lysates of Sf21 cells. Our long-term goal is to clone a single-chain antibody (scFv) gene encoding Mab 7D6 into the baculovirus to enhance the virus’s potential as a biological control agent. We anticipate that fall armyworm larvae infected by the recombinant viruses will die more quickly than those infected by wild-type viruses, as antibodies produced within insect cells bind to EF-1□ and disrupt metabolism, either by preventing the synthesis of proteins vital to the host cell, or by enhancing production of infective virions by delaying apoptosis. Because immunologically distinct, tissue-specific forms of EF-1□ commonly occur in eukaryotes, tissues of fall armyworm larvae were probed with Mab 7D6 to determine whether the tissues most important for establishing viral infection (midgut, trachea, and fat body) were recognized. Using western blotting, ELISA, and immunofluorescence microscopy techniques, we found that all tissues examined contained measurable amounts of EF-1□ reactive with Mab 7D6, although concentrations varied among different cell types within a given tissue. In the midgut, the intensity of the signal was much stronger on the apical part of the columnar epithelial cells, especially on the brush border microvilli, than on the basal parts of these cells. No signal was observed on goblet cells or basement membrane.

### RNAi in function studies of tick genes

O. Hajdusek, D. Sojka, V. Buresova, Z. Franta, M. Vancova, P. Kopacek, L, Grubhoffer

Institute of Parasitology, Biological Centre Academy of Sciences of the Czech Republic, Ceske Budejovice, Czech Republic and Faculty of Biological Sciences, University of South Bohemia, Ceske Budejovice, Czech Republic. Correspondence: hajdus@paru.cas.cz

The hard tick *Ixodes ricinus* is an important vector of Lyme disease and tick-borne encephalitis. RNAi is an excellent method for silencing of genes in vivo and allows us to study function of molecules involved in tick innate immunity. So far, we have studied five genes linked with tick immunity and iron storage: Chitinase1 (Chix1), alpha-2-macroglobulin (TAM), IxoderinA (IxoA), Ferritin (FER) and Cytoplasmic aconitase (Iron responsive protein - IRP). Chix1 is an enzyme secreted by tick salivary glands. The hemolymph of ticks treated with Chix1 dsRNA is much darker, viscose and its volume is markedly decreased. We speculate, whether Chix1 is a molecule important for tick hemolymph homeostasis and/or a component of tick saliva, which interferes with the host immune response against the feeding tick. FER is an intracellular iron storage protein of ticks. Its knock-down leads to the lower weight of fed ticks and influences ticks vitality and fertility. IRP is a potential translation inhibitor of FER and other proteins involved in iron metabolism. Here, we study relation between FER and IRP. IxoA is a fibrinogen-like molecule (a homolog of previously characterized DorinM, a lectin from the plasma of soft tick *O. moubata*) found mostly in the tick hemolymph. TAM is a protease inhibitor playing an important role in the innate immunity by guarding against the undesired action of proteases of a different origin, including those from invading pathogens. RNAi studies of these two molecules promise interesting insight into the function of molecules involved in pathogen recognition and inhibition.

### Molecular structure of two circadian clock genes, *period* and *timeless*, and their possible roles in photoperiodic determination of diapause in the flesh fly, *Sarcophaga bullata*

B. Han and D. L. Denlinger

Department of Entomology, The Ohio State University, 318 W 12^th^ Ave, Columbus, OH 43210, USA. Correspondence: han.163@osu.edu

The flesh fly, *Sarcophaga bullata*, normally exhibits a circadian pattern of adult eclosion, with flies emerging at dawn. This species also uses photoperiodic cues of short daylength to program its pupal diapause. A mutant strain of *S. bullata* shows both an arrhythmic pattern of eclosion time and a lack of diapause. We targeted candidate genes that are possibly involved in the loss of rhythmicity: *period* and *timeless*. The products of these two genes, PERIOD and TIMELESS, can form a dimmer, whose formation and dissociation are the primary determinants of the oscillatory timekeeping system. The full length cDNA sequences of the *period* and the *timeless* genes in *S. bullata* were obtained using RT-PCR followed by RACE (Rapid Amplification of the cDNA Ends). The mRNA’s of the two genes are 4,066 and 5,993 base pairs long, respectively. They encode a PERIOD protein of 1040 amino acids and a TIMELESS protein consisting of 1591 amino acids, with a 91% similarity to those genes in *Drosophila melanogaster*. mRNA splicing patterns under different photoperiodic conditions are being examined for both wild type and mutant flies to help explain the potential relationship between circadian and seasonal clocks.

### Cationic amino acid transporters mediate amino acid / target of rapamycin signaling during reproduction in the yellow fever mosquito, *Aedes Aegypti*

I.A. Hansen^1^, G.M. Attardo^2^, S.H. Shiao^1^, D. Voronov^3^; D. Boudko^3^, and A.S. Raikhel^1^

^1^Center for Disease-Vector Research, and Department of Entomology, University of California, Riverside, CA, USA. Correspondence: immoh@ucr.edu

^2^Present Address: Yale School of Public Health, Yale University, New Haven, CT, USA

^3^ The Whitney Laboratory, St. Augustine, FL, USA.

Anautogenous mosquitoes require vertebrate blood to initiate reproduction. The need for blood drives the association of vector and host, and is the primary reason why anautogenous mosquitoes are effective disease vectors. During mosquito vitellogenesis, a key process in reproduction, *yolk protein precursor* gene expression is activated specifically in the fat body. Blood meal-derived amino acids activate *yolk protein precursor* genes via the target of rapamycin (TOR) signal transduction pathway. Here, we show by stimulating fat bodies with balanced amino acid solutions lacking individual amino acids that specific cationic and branched amino acids are essential for activation of the *vitellogenin* gene, the major *yolk protein precursor* gene. Treatment of fat bodies with amino acid uptake inhibitors results in a strong inhibition of amino acid-induced *vitellogenin* gene expression, proving that a facilitated transport mechanism is necessary to transduce the amino acid signal. We cloned two cationic amino acid transporters from the fat body of *Aedes aegypti* females – *Aa slimfast* and *iCAT2*. RNAi knockdown of *slimfast and iCAT2* results in a strong decrease in *vitellogenin* gene induction in response to amino acids, which is similar to TOR inhibition. Functional heterologous expression in *Xenopus* oocytes and electrochemical analysis revealed that *slimfast* is a sodium- and potassium-independent cationic amino acid transporter with strong preference to histidine. Our data demonstrates that mediated uptake of specific amino acids plays a key role in nutritional signaling during the onset of vitellogenic gene expression in mosquitoes and stress the importance of cationic amino acid transporters in this process.

### Hemolin gene and its protein expression in the silk gland and spun-out silk of the wax moth *Galleria mellonella*

Haq A.S. and Sehnal F.

Institute of Entomology, Academy of Sciences, Ceské Budajovice, Czech Republic. Correspondence: sehnal@entu.cas.cz

Hemolin, a protein of the immunoglobulin superfamily, is produced in insect fat body and gut and circulates in the hemolymph. It participates in the immune defense system and may also play a role in morphogenesis as a cell adhesion molecule. We have identified the hemolin gene in the wax moth, *Galleria mellonella* and detected its expression in the silk glands and the central nervous system. No *hemolin* mRNA was detected in the feeding larvae in the middle of the last larval instar unless they were challenged by bacteria or injury. Removal of the head and thorax by ligation also induced gene expression that persisted for many days and could be enhanced with small injuries and injections of bacteria, peptidoglycans and lipopolysacharides. In the intact insect, the gene was expressed spontaneously at the onset of metamorphosis at the end of the last larval instar. Hemolin protein was detected by Western blotting in the spun-out cocoon silk. The work was in part supported by grant A5007402 from the Grant Agency of the Academy of Sciences.

### Novel resistances against BT toxin were found and could be mapped on the molecular genetic map in the silkworm

W. Hara^1^, K. Miyamoto^2^, O. Ninaki^□^and K. Kanda^3^

^1^National Institute of Agrobiological Sciences, Ohwashi 1-2, Tsukuba, Ibaraki, Japan. Correspondence: whara@affrc.go.jp

^2^Tokyo University of Agriculture and Technology, Futyu, Tokyo, Japan

^3^Saga University, Saga, Saga, Japan

We have developed methods for making molecular maps by improving classical methods, using linkage analysis by complete linkage on BC1 and mapping by three-point analysis. The cDNA-markers showing RFLP could be easily determined from their linkage group by scanning linkage analysis (Hara et al., 2002, Kadono-Okuda et al., 2002). Their location on the chromosome could be decided by repeated three-point analysis (Nguu et al., 2005). The new methods could map a novel resistant gene against BT toxin (Cry1AB) on the chromosome (Hara et al., in preparation). The cDNA clones’ RFLP seem to be convenient because they show co-dominant character and generally detected in a inter-specific and intra-specific manner and these markers and methods could be introduced to other Lepidopteran insects. Less than 20 silkworm strains were screened to determine their resistance against BT toxin, Cry1Ab, and new two strains showed recessive resistance and twelve strains showed dominant resistances. These new resistances are now examined genetically, whether they are same gene or not and where they are on the molecular genetic map. These kinds of mutants could present the model systems to know the mechanisms how the toxins work and the resistances against BT-toxin in insects.

### Proteomic analysis of *Anopheles gambiae* cast cuticles by tandem mass spectrometry

N. He^1^, J. Batelho^2^, V. Belozerov^3^, W.A. Dunn^1^, R. Orlando^2^, J.H. Willis^1^

^1^Department of Cellular Biology, University of Georgia, Athens, GA, USA. Correspondence: hejia@cb.uga.edu

^2^Complex Carbohydrate Center, University of Georgia, Athens, GA, USA

^3^Department of Neurosurgery, Emory University School of Medicine, Atlanta, GA, USA.

Over 130 sequences for putative cuticular proteins have been manually annotated in the *Anopheles* whole genome sequence. In order to learn which of these corresponds to proteins actually found in the cuticle, we have carried out a proteomic analysis of cuticles cleaned by *Anopheles* itself and left behind as cast pupal cuticles or larval head capsules. Proteins were extracted, fractionated by 1D SDS gel electrophoresis and large gel slices were reduced, carbamidomethylated and digested with trypsin. The resulting peptides were separated on a C18 column and detected by ion-trap mass spectrometry. The resulting MS/MS data were analyzed with Mascot and Sequest using a combination of three databases from the translated genome. Seventy-seven distinct peptides were detected, corresponding to a maximum of 28 different cuticular proteins and comprising the first authentic *Anopheles* cuticular proteins. Shared among both cuticle sources were 15 proteins, while 5 were unique to the pupal cuticle, and 8 to the head capsule. Sequence coverage ranged from 4% to 60% of the total mature protein. These structural proteins come from several distinct families, some previously unknown. In addition, we found peptides corresponding to 15 proteins that appeared to come from molting fluid trapped in the cast pupal cuticles and 27 proteins from muscles still associated with the cast head capsules.

### Insect immunity: Role of mosquito pericardial cells during the response against pathogens and malaria parasites

Salvador Hernández-Martínez^1, 2^, Fernando Noriega^2^, Arti Navarre^3^, Facundo Fernandez^3^, Mario H. Rodríguez^1^ and Humberto Lanz^1^

^1^Centro de Investigaciones Sobre Enfermedades Infecciosas INSP. Mexico

^2^Department of Biological Sciences, Florida International University. USA, Correspondence: hernands@fiu.edu

^3^School of Chemistry and Biochemistry. Georgia Institute of Technology. USA

Innate immunity is a widespread and essential defense mechanism against microbial attacks. The fat body and hemocytes are major players in the insect innate immune response; however, other tissues such as midgut, epidermis and malpighian tubules participate as well. We decided to explore the role of mosquito pericardial cells during the immune response against pathogens, including malaria parasites. Pericardial cells are located around the dorsal blood vessel, a flexible tube that runs longitudinally through the thorax and abdomen, helping the flow of hemolymph. Putative roles for pericardial cells (nephrocytes) are the filtration, clearance and regulation of hemolymph composition Important markers of the immune response (for example: STAT; *Sp22D*, TEP-I and SRPN10) have been reported to be present in mosquito pericardial cells, suggesting that they could be an important component in the mosquito immune system. We are using biochemical, cellular, ultrastructural, and MS spectrometry approaches to analyze the response of pericardial cells of *Anopheles albimanus*, a vector of *Plasmodium vivax* in Mexico, during a challenge with different microorganism, including malaria infection. We propose that pericardial cells produce and release immune molecules during infection, thus playing an important role in the clearance of invading pathogens.

### Regulation of juvenile hormone synthesis in mosquito: physiological, biochemical and molecular studies

S. Hernandez-Martinez^1, 2^, J.G. Mayoral^1^, M.H. Perez^1^ and F.G. Noriega^1^

^1^Department of Biology, Florida International University, Miami, Fl., USA. Correspondence: e.mcgraw@uq.edu.au

^2^ Centro de Investigaciones Sobre Enfermedades Infecciosas INSP. México.

Juvenile hormone (JH) is a major hormonal regulator in insects. In the female mosquito, JH signals the completion of the ecdysis to the adult stage, and initiates reproductive processes. The aims of our studies are: 1) to understand the regulation of JH levels in mosquitoes, and 2) To understand how nutritional signals affect the activity of the neuroendocrine system. JH titer is essentially determined by the rate at which the corpora allata (CA) synthesizes JH. The rate of CA activity is, in turn, regulated by allato-regulatory peptides that exert either allatostatic (inhibitory) or allatotropic (stimulatory) activities. We have described that *Aedes aegypti* allatotropin (AT) stimulates and *Aedes aegypti* allatostatin-C (AS-C) inhibits JH synthesis; in addition we have showed that nutrients accumulated during the larval stages regulate the CA activity in newly emerged adults. Based on this work we propose that AT and AS-C released by the brain are important for the activation and modulation of JH synthesis in adult female mosquitoes. The synthesis and release of these peptides might be connected to nutritional signals. JH is therefore an important part of a transduction mechanism that connects changes in the nutritional status with activation of specific physiological events during reproduction. In order to test this model we performed the first genomic analysis of an insect endocrine gland; libraries were made from corpora allata-corpora cardiaca complexes from *Aedes aegypti* and *Anopheles albimanus.* More than 1800 clones have been sequenced and enzymes involved in JH synthesis and other important regulatory molecules have been identified among these clones. We are using these molecular tools to investigate the mechanisms of control of JH synthesis by AT and AS-C and to study the nutritional regulation of synthesis and release of AT and AS-C in the brain.

### Bursicon, the insect cuticle sclerotizing neurohormone: sequence, receptor and beyond

H.-W. Honegger

Department of Biological Sciences, Vanderbilt University, Nashville, TN 37235-1634. Correspondence: h.willi.honegger@vanderbilt.edu

Bursicon was discovered in 1962 by Fraenkel and Hsiao as a new hormone that initiates the darkening and hardening (tanning) of the cuticle of freshly emerged blowflies using the “ligated fly bioassay”. We and Mendive et al. (2005) have found that Bursicon is a heterodimer composed of two highly conserved cystine knot proteins encoded by the *Drosophila melanogaster* genes CG13419 and CG15284. The large vertebrate cystine knot protein family contains signaling proteins such as TGF-β and glycoprotein hormones. Bursicon is the first known member of this family in insects. Recombinant heterodimeric protein (but not the homodimers) expressed in 239T cells causes complete darkening of flies in the ligated fly bioassay. It also initiates cAMP production by 239T cells transfected with plasmids expressing the G-protein coupled receptor (GPCR) DLRG2, thus showing that this GPCR is the bursicon receptor. Its specificity was supported by experiments showing that Bursicon action could not be out-competed by similar vertebrate heterodimeric cystine knot proteins nor by bursicon homodimers. Drosophila mutants at CG13419 gene locus showed that Bursicon is also responsible for wing inflation; recent elegant studies by White et al. (2006) have supported this finding. Bursicon is released from the nervous system by large lateral neurosecretory cells present in all ganglia of the ventral CNS in different insects. These cells also express the neuropeptide crustacean cardioactive peptide (CCAP). Immunocytochemistry shows that the Bursicon heterodimer is expressed in these cells, but exceptions occur in some insects during development. EM studies indicate that in Bursicon producing cells, Bursicon and CCAP are packaged in the same vesicles. These findings support the hypothesis that CCAP and Bursicon are co-released, suggesting that CCAP facilitates distribution of Bursicon to its target sites. Immunocytochemistry also indicates that in the moth, *Manduca sexta*, the cellular pattern of Bursicon expression in the CNS is similar to that in other insects. However in *Manduca*, large intrinsic cells of the corpora cardiaca express only one homodimer, and these cells co-produce AKH (see also the abstract by Dai, Honegger and Adams). It will be outlined that use of *Drosophila* genetics may reveal other as yet unresolved functions of Bursicon.

### Stage- and tissue-specific alternative splicing of *Manduca sexta* allatotropin mRNA and evidence for the presence of predicted allatotropin-like peptides in cells of the larval terminal abdominal ganglion

F.M. Horodyski^1^, K.-Y. Lee^1,2^, S. Neupert^3^, R. Predel^3^, and N.A. Davis^1^

^1^Department of Biomedical Sciences, Ohio University, Athens, OH USA. Correspondence: horodysk@ohio.edu

^2^Department of Agricultural Biology, Kyungpook National University, Daegu, Korea

^3^Saxon Academy of Sciences, Jena, Germany

*Manduca sexta* allatotropin (Manse-AT) is a multifunctional neuropeptide that is expressed as at least three alternatively spliced mRNAs in a stage- and tissue-specific manner. Two of these mRNAs are predicted to encode three additional allatotropin-like (ATL) peptides which possess biological activities that overlap with those of Manse-AT. However, evidence for the production of the ATL peptides has thus far been lacking. We generated polyclonal antisera to Manse-ATL-II and report the staining of specific cells with this antiserum in larval *M. sexta*. The most intense staining was observed in two cells in the terminal abdominal ganglion (TAG) whose axons project posteriorly and exit the CNS. Two cells in the brain and one cell in the subesophageal ganglion showed weak Manse-ATL-II-like immunoreactivity. Staining was completely blocked by preabsorption of the antiserum with synthetic Manse-ATL-II, but was unaffected by preabsorption with Manse-AT, Manse-ATL-I, or -III. Our previous demonstration of Manse-AT RNA-3 (which encodes Manse-ATL-II) in the larval TAG is consistent with these immunohistochemical staining results. Analysis of the peptide content of the Manse-ATL-II immunoreactive cells in the larval TAG revealed the presence of peptides whose masses are consistent with those of Manse-AT, Manse-ATL-I, and Manse-ATL-II. These peptides are the predicted products of Manse-AT RNA-3. These data demonstrate that Manse-ATL-II-like immunoreactivity is present in a subset of cells that contain Manse-AT immunoreactivity suggesting that alternative splicing of Manse-AT mRNAs occurs in a cell-specific manner. This work was supported by the NSF.

### Molecular dissection of the *Bombyx mori* pheromone biosynthesis activating neuropeptide receptor

J. J. Hull, A. Ohnishi, and S. Matsumoto

Molecular Entomology Laboratory, RIKEN, 2-1, Hirosawa, Wako, Saitama 351-0198, Japan. Correspondence: joeh@postman.riken.go.jp

As with most lepidopteran species, the sex pheromone biosynthetic pathway in the silkmoth, *Bombyx mori,* is triggered by a molecular interaction between pheromone biosynthesis activating neuropeptide (PBAN) and its cognate receptor, PBANR. In *B. mori*, PBANR is a 413 amino acid G protein-coupled receptor that is distinguishable from other putative PBANRs by an extended carboxyl terminus that is necessary for agonist induced internalization. To provide a more extensive determination of structure-function relationships for the *B. mori* PBANR, we constructed a series of amino terminal and carboxyl terminal truncation mutants, in addition to a number of point mutations and analyzed their effects on receptor expression and internalization when transiently expressed in Sf9 cells. To date, our results suggest that the first 27 residues of the *B. mori* amino terminus are not necessary for receptor expression or ligand binding; the receptor internalization motif resides between residues 357–366 and may involve the endosomal targeting motif, YxxL; and that Ser333, Ser366, Arg263, and Arg264 likely play important roles in receptor function and/or regulation. Furthermore, we have shown that the regulatory mechanism in Sf9 cells is calcium dependent, relies on kinase activity, and proceeds through clathrin-coated pits.

### Purification and characterization of a gelatinolytic protease from the degenerating larval intestine of *Bombyx mori*

K. Kaji, T. Asano, and S, Izumi

Department of Biological Sciences, Biochemical Laboratory, Tokyo Metropolitan University. Correspondence: kaji-kentaro@c.metro-u.ac.jp

The larval intestine undergoes stage-dependent degeneration after the larval-pupal metamorphosis in *Bombyx mori*. Zymographic analysis of the intestinal extract demonstrated that a gelatinolytic protease with molecular weight around 37k (37k protease) appears in intestine after the larval-pupal molt, and its activity progressively rises thereafter. We purified 37k protease from the pupal intestine of *B. mori* and determined partial amino acid sequences. The 37k protease preferentially degraded the peritrophic membrane’s protein, and its activity was inhibited by inhibitors for trypsin-like proteases. Immunoblot analysis with anti-37k protease antibody showed that the protease or pro-protease appears around pupation and exists until the 6^th^ day of pupal stage in intestine. Based on the amino acid sequences, we got an EST clone encoding the 37k protease. cDNA analysis revealed that this protease consists of 329 amino acid residues and has significant structural similarity with serine proteases. mRNA for 37k protease was detected in RNA isolated from larval intestine 2 days after silk-spinning by Northern blotting. Recombinant pro-37k protease synthesized by the baculovirus expression system was activated by a tissue extract prepared from the intestine from the silk-spinning stage. These results suggest that 37k protease is activated by the proteolytic degeneration that occurs in the intestine after the larval -pupal ecdysis.

### Epicuticular wax of large and small white butterflies, *Pieris brassicae* and *P. rapae crucivora*: qualitative, quantitative and scanning electron microscopic studies of diapause and non-diapause pupae

Jun-ichi Kaneko^1^, Hirokuni Yamada ^2^, and Chihiro Katagiri^3^

^1^National Agricultural Research Center for Hokkaido Region, Sapporo, 062-8555, Japan. Correspondence: jkpure47@affrc.go.jp

^2^School of Medicine, Sapporo Medical University, Sapporo, 060-8556, Japan

^3^Institute of Low temperature Science, University of Hokkaido, Sapporo, 060-0819, Japan

We compared the quantity and quality of the epicuticular wax of diapause and non-diapause pupae in two closely related *Pieris* species, *P. brassicae* and *P. rapae crucivora*. Main components of their epicuticular wax were identified as hydrocarbons. In *P. brassicae*, more than 95% of hydrocarbons were saturated regardless of whether the pupae were in diapause or not. In *P. rapae crucivora,* 93% of hydrocarbons were saturated in non-diapause pupae whereas in diapause pupae 41% were saturated and 59% unsaturated. From measurements of body surface area by nuclear magnetic resonance microimaging, we calculated the average thickness of the wax layer. The thickness in diapause and non-diapause pupae of *P. brassicae* was 800 and 160 nm, respectively. In *P. rapae crucivora*, the thickness was 195 nm in diapause and 11 nm in non-diapause. We also compared the surface textures of their diapause and non-diapause pupae by scanning electron microscopy.

### Characterization of white colored waxy strands of woolly ash aphid, *Prociphilus oriens*

C. Katagiri^1^**,** H. Yamada ^2^**,** Y. Miyashita^3^, and S. Akimoto^4^

^1^Biochemistry Laboratory, Institute of Low Temperature Science, University of Hokkaido, Sapporo, 060-0819, Japan. Correspondence: ck@lowtem.hokudai.ac.jp

^2^Department of Physics, School of Medicine, Sapporo Medical University, Sapporo, 060-8556, Japan

^3^Department of Biology, School of Medicine, Sapporo Medical University, Sapporo, 060-8556, Japan

^4^Department of Ecology and Systematics, Graduate School of Agriculture, Hokkaido University, Sapporo, 060-8589, Japan.

Aphids are very unique insects. They may have multiple host plants and widely varying shapes through a year. A woolly ash aphid, *Prociphilus oriens* (Mordvilko) alternates host plants seasonally between the primary host ash tree *Fraxinus mandshurica* and the secondary host fir tree *Abies sachalinensis*. In Sapporo, the female winged generation appears on the secondary host fir tree before the snow falls and migrates to the primary host ash tree. Since its appearance with white colored waxy strands tells us the snow season is coming soon, we call it a snow bug. The winged females parthenogenetically produce males and females on the trunk of ash trees. After copulation, a single egg is laid in bark crevices. The eggs overwinter there and hatch before the ash trees bud. From the viewpoint of genetic variance, selfing and outbreeding, woolly ash aphids have been mainly studied. In the present study, we focus the chemistry and morphology of white colored waxy strands of winged female generation that appears in late autumn. The waxy substances are saturated hydrocarbons. Using a scanning electron microscopic study, we discuss how the waxy strands are formed.

### Exocytosis and endocytosis at the *Drosophila* neuromuscular junction

Yoshi Kidokoro

Gunma University, Maebashi, Japan and David Geffen School of Medicine at UCLA, California, USA. Correspondence: kidokoro@med.gunma-u.ac.jp

At synapses neurotransmitters are released by exocytosis of synaptic vesicles, and the vesicle membrane is retrieved by endocytosis and recycled. Both of these two processes require external Ca^2+^. For exocytosis Ca^2+^ influx occurs synchronously upon arrival of an action potential at the presynaptic terminal. It is not clear, however, how Ca^2+^ influx initates endocytosis. At the *Drosophila* neuromuscular junction we examined the distribution of Ca^2+^ channels encoded by the *cacophony* (*cac*) gene. *cac*-Ca^2+^ channels form clusters at the presynaptic active zone. These clusters are heterogenous in size and distributed unevenly in the presynaptic boutons. The distribution of *cac* Ca^2+^ channel clusters correlates well with that of synaptic vesicles. In *cac*-null embryos fast synaptic transmission is completely blocked, indicating that *cac* Ca^2+^ channels are the sole Ca^2+^ channel that controls fast synaptic transmission. Even in *cac*-null embryos delayed release of synaptic vesicles occurs in elevated external Ca^2+^, suggesting another type of Ca^2+^ channel is located close to the release site. These non-*cac* Ca^2+^ channels are probably regulating endocytosis that occurs in the active zone. We further identified the third type of Ca^2+^ channel that is blocked by a low concentration of La^3+^. Since 50 μM La^3+^ blocks endocytosis without affecting exocytosis, it is likely that this type of Ca^2+^ channel is regulating endocytosis. Thus multiple types of Ca^2+^ channels are coordinately regulating exo- and endocytosis in the presynaptic terminal.

### Gene structure and enzyme activity of phospholipase A_2_ of *Spodoptera exigua*, which is a pathogenic target of an entomopathogenic bacterium, *Xenorhabdus nematophila*

Y. Kim^1^**,** S. Cho^2^, and S. Shresta^1^

^1^Insect Molecular Physiology Laboratory, Andong National University, Andong 760-749, Korea. Correspondence: hosanna@andong.ac.kr

^2^Korea-Bio Institute, Inc., Suwon 445-964, Korea

*Xenorhabdus nematophila* is an entomopathogenic bacterium symbiotically associated with an entomopathogenic nematode, *Steinernema carpocapsae*. When the nematode infects a target insect, *X. nematophila* is released from the symbiotic nematode gut to the insect hemocoel. The bacteria inhibit both cellular and humoral immune capacity of the infected insect. Eicosanoids play an important role in mediating cellular immunity in response to bacterial infection. *X. nematophila* can shut down the eicosanoid biosynthesis by inhibiting phospholipase A_2_ (PLA_2_). This study shows the significant PLA_2_ activities in hemocyte, fat body, and gut tissues of *Spodoptera exigua*. The PLA_2_s from different tissues and subcellular fractions are varied in catalytic properties. An inducible PLA_2_ has been found in hemocytes by an antibody raised against secretory type PLA_2_. Based on the conserved amino acid sequences of Group III sPLA_2_, degenerate primers were constructed and used to clone PLA_2_ from *S. exigua* hemocytes. The cloned cDNA of the hemocyte PLA_2_ is 1,050 bp long. Its deduced amino acid sequence (282 residues) shares some homology with Group III sPLA_2_, but differs in the lack of calcium binding site as well as the amino acid sequence. The PLA_2_ gene was specifically expressed in hemocytes and induced in response to various pathogens including laminarin, lipopolysaccaride, Gram positive and negative bacteria. The induced hemocyte PLA_2_ exhibited a 1.2 kb RNA transcript by a Northern hybridization analysis. The gene was expressed in a bacterial expression system and purified as ≈ 30 kDa protein. The purified PLA_2_ exhibited significant enzyme activity.

### Identification of aphid-repellent indole glucosinolate breakdown products

J. H. Kim and G. Jander

Boyce Thompson Institute for Plant Research, Ithaca, NY 14850. Correspondence: gj32@cornell.edu

Plants have evolved a variety of physical and chemical barriers to protect themselves against herbivory. In a characteristic defense of cruciferous plants, tissue damage brings a class of compounds called glucosinolates into contact with the enzyme myrosinase to produce isothiocyanates, nitriles, and other sharp-tasting volatiles that can deter herbivory. Unlike most chewing herbivores, the phloem-feeding *Myzus persicae* (green peach aphid) is able to avoid myrosinase-catalyzed glucosinolate cleavage. However, the tryptophan-derived indole glucosinolates nevertheless break down during passage through the aphid gut. Experiments with Arabidopsis mutants and purified indole glucosinolates in artificial diets demonstrated that indole glucosinolate breakdown in the aphid gut has a deterrent effect. Moreover, bioassays performed with compounds detected in the honeydew of aphids feeding on Arabidopsis identified specific indole glucosinolate breakdown products that are aphid-repellent. Therefore, this alternate glucosinolate breakdown pathway may represent a plant defense against phloem-feeding herbivores such as *M. persicae* that manage to avoid the myrosinase-catalyzed activation of glucosinolates.

### Cloning and characterization of vermilion and white eye-color genes from the honey bee, *Apis mellifera*

K. Kimura

Laboratory of Apiculture, Department of Animal Breeding and Reproduction, National Institute of Livestock and Grassland Science, Tsukuba, Ibaralki 305-0901 Japan. Correspondence: kimura@affrc.go.jp

In order to exploit the advantage offered by the eye-color markers in transformed selection, eye-color genes from the honey bee, *Apis mellifera,* have been cloned. Candidate DNA sequences for orthologs of the *Drosophila melanogaster* eye-color genes *vermilion* (tryptophan oxygenase) and *white* (ATP-binding cassette transporter protein) were isolated from the honey bee genome data base. RNAi experiments using dsRNA designed from these sequences confirmed that these sequences were the orthologs of eye-color genes of *Drosophila*. The deduced amino acid sequences for the honey bee *vermilion* and *white* showed 68.5% and 64.8 % similarity to those proteins from *Drosophila* respectively, and they shared conserved motifs with those genes from other insects. Genomic sequences for the coding region of these genes from other *Apis* species were also recovered by PCR. The results of further characterization of these genes will be reported.

### Surfing the web: Spider toxins and their potential for insect control

Glenn F. King

Department of Molecular, Microbial & Structural Biology, University of Connecticut School of Medicine, Farmington CT 06032-3305, USA. Correspondence: glenn@psel.uchc.edu

Excluding insects, which are their primary prey, spiders are the most successful terrestrial invertebrates. Whereas most invertebrates produce an array of neuropeptides for internal regulation of various physiological and behavioral processes, spiders synthesize in their venom glands a combinatorial library of neuropeptides that are designed to kill or paralyze envenomated prey. These neurotoxins are initially produced as prepropeptide precursors that are posttranslationally processed to yield the mature toxins. The complete venom is often remarkably complex—recent peptidomic analyses reveal that a single spider venom can comprise more than 1000 different peptide toxins. We have been exploring the potential of spider venoms to contribute to the development of insecticides with novel modes of action. I will demonstrate that it is possible to isolate spider toxins that target specific insect ion channels but which have no effect on the vertebrate counterparts of these channels. Moreover, I will show that Australian funnel-web spiders have evolved a neurotoxin that acts simultaneously on two different ion channels in a self-synergizing manner. In essence, this dual-target toxin corresponds to a toxin cabal encoded within a single polypeptide chain. The implications of using such a dual-target approach for the development of novel insecticides will be discussed.

### Pacheco’s classification of the variegated mud-loving beetles: Natural or not?

Jonas G. King, Paul K. Lago and Julian R. Starr

Department of Biology, University of Mississippi, University, MS 38677. Correspondence: jgking1@olemiss.edu

The Heteroceridae is a taxonomically difficult family of small (2–7 mm) beetles that live in self-constructed feeding tunnels along the shores of fresh and brackish waters worldwide. Traditional classifications of Heteroceridae accept only five genera (*Heterocerus, Micilus, Augyles, Phyrites* and *Elythomerus*) (Coleoptera: Byrrhoidea: Heteroceridae), but the most recent revision of the family recognized nineteen (Pacheco, 1964), with twelve segregated from the type genus alone. These new genera, which are based on characters of the male genitalia, have not been widely accepted. In order to gain a better understanding of generic limits and relationships, a phylogenetic study using nuclear 28S rDNA and EF-1α gene sequences was undertaken. Phylogenetic analyses support (100% bootstrap) the Heteroceridae as monophyletic, and indicate (83% bootstrap) that it is sister to the Limnichidae within the superfamily Byrrhoidea, a relationship not resolved by a previous morphological study (Lawrence et al., 1995). Within Heteroceridae, analyses firmly position (100–99% bootstrap) *Tropicus pusillus* and *Centuriatus auromicans* successively as sisters to a strongly supported (94% bootstrap) terminal clade containing all of the genera segregated from *Heterocerus* s.l. by Pacheco. Relationships within this clade indicate that the genera *Neoheterocerus* and *Lanternarius* sensu Pacheco are unnatural as circumscribed, suggesting that the traditional classification of the family may better reflect relationships. Attempts to increase taxonomic sampling and to add further markers are currently under way.

### Larval RNA interference in the neuropteran *Chrysopa* and the silverfish *Thermobia* for studies on ecdysone signaling genes

Barbora Konopova^1^ and Marek Jindra^2^

^1^Department of Molecular Biology, University of South Bohemia

^2^Biology Center CAS, Branisovska 31, Ceske Budejovice 37005, Czech Republic. Correspondence: jindra@entu.cas.cz

RNA interference (RNAi) is a vital method for demonstrating gene function in non-model species. By utilizing RNAi, we want to compare developmental roles of steroid hormone (ecdysone) response genes across insect orders. To develop systems evolutionarily relevant for such comparative studies, we chose two insect species: the lacewing *Chrysopa perla* (Neuroptera) and the silverfish *Thermobia domestica* (Zygentoma). While neuropterans represent one of the most primitive orders displaying holometabolous development, *Thermobia* is an apterygote that lacks metamorphosis altogether. Nonetheless, the key components of the ecdysone signaling pathway are well conserved in these insects. We isolated partial cDNA clones (600–800 bp in length) for the *ecdysone receptor* (*EcR*), *ultraspiracle* (*usp*), *E75* and *broad-complex* (*BR-C*) genes from *Chrysopa*, and *EcR*, *usp*, *BR-C*, *E75*, *ftz-f1* and the hormone receptor genes *HR4* and *HR38* from *Thermobia*. We then tested both species for susceptibility to RNAi targeting of some of these genes. Injection of early *Chrysopa* larvae with double-stranded RNA (dsRNA) against either of the components of the ecdysone receptor complex (EcR or Usp) caused developmental arrest and death still in the larval stages. By contrast, RNAi targeting of *BR-C*, which is necessary for pupal development in both *Drosophila melanogaster* (Diptera) and the silkworm *Bombyx mori* (Lepidoptera), caused no anomalies until the onset of metamorphosis, when the animals were unable to molt into the pupal stage and in more severe cases also failed to spin the cocoon. Preliminary results showed that larval molting could be disrupted by injection of *EcR*, *usp* or *E75* dsRNA into second to fourth instar *Thermobia* larvae, while *BR-C* RNAi allowed development of adults. These data suggest that BR-C is causally linked with metamorphosis as early as in the most primitive holometabolans but that it may play another role in ametamorphic insects. Supported by grant A5007305 from the Czech Academy of Sciences.

### Pheromone signaling in moths: Identification and characterization of receptors and binding proteins

J. Krieger, T. Gohl, E. Große-Wilde and H. Breer

University of Hohenheim, Institute of Physiology, Garbenstrasse 30, 70599 Stuttgart, Germany. Correspondence: krieger@uni-hohenheim.de

Pheromones initiate and control mating behavior in many insects. To recognize and discriminate female released pheromones the antennae of male moths have evolved to high-performance pheromone-detectors with extreme sensitivity and selectivity. Their remarkable capacity is based on spezialized chemosensory neurons housed in sensilla hairs on the antenna. These cells detect the species-specific pheromone signal and convert the chemical information into electrical neuronal responses. To reach their specific receptors on the surface of the olfactory neurons pheromone molecules enter the antennal sensilla through pores in the cuticle and have to traverse an aqueous barrier, the sensillum lymph. This process is supposed to be mediated by soluble pheromone binding proteins (PBPs), which ferry the hydrophobic pheromonal compounds towards the dendritic membrane of the sensory cells where they interact with seven transmembrane domain receptor proteins. This initiates intracellular transduction cascades generating electrical responses of the cells. We have identified a small family of genes encoding candidate pheromone receptors in the tobacco budworm *Heliothis virescen*s and the silkmoth *Bombyx mori*. Several of these genes were found to be selectively expressed in the antennae of male moths. *In situ* hybridization studies revealed that expression of these receptor types was confined to antennal cells, which were surrounded by cells expressing PBP and were located beneath sensillar hair structures (sensilla trichodea) containing pheromone sensitive neurons. Using receptor-specific antibodies the receptor protein was visualized in sensory dendrites projecting into these sensilla. To approach the ligand specificity of candidate pheromone receptors, cell lines expressing receptors were assessed for their response to pheromonal compounds. The results of calcium-imaging experiments indicated that expression of candidate pheromone receptors rendered HEK cells responsive to low concentrations of pheromone components. In addition, le ligand specificity. These data support the view that both distinct pheromone receptors and binding proteins play an important role in insect pheromone recognition.

### Enhancement of the baculovirus expression vector system by *Campoletis sonorensis* ichnovirus proteins

Jeremy Kroemer^2^, Angelika Fath-Goodin^1^, Stacy Martin^2^, Krista Reeves^1^ and Bruce Webb^1^

^1^University of Kentucky, Department of Entomology, Lexington, KY 40546. Correspondence: bawebb@uky.edu

^2^ParaTechs Corp., Lexington, KY 40546

The Baculovirus Expression Vector System (BEVS) is a powerful and versatile tool for recombinant protein expression. Advantages of the system include high protein expression levels, larger limits to protein size, efficient protein processing, post-translational modifications, and simultaneous expression of multiple gene casettes. However, a major limitation of the lytic BEVS is that death and lyses of infected insect cells ends protein production. This results in delay and higher production costs due to the need to set-up new infections, maintain uninfected cells, and reproduce pure viral stocks. We have identified proteins from the insect virus *Campoletis sonorensis* ichnovirus (*Cs*IV) that delay lysis of baculovirus-infected cells, resulting in significant enhancement of recombinant protein production in the BEVS system. Recombinant protein production in the CsIV protein-enhanced BEVS is increased by a factor of 4–15 fold. Co-expression of yellow fluorescent protein (YFP) and the *Cs*IV protein from a dual BEVS resulted in an up to 15-fold increase in YFP production and delayed lysis of infected insect cells when compared to the control BEVS expressing only YFP. When stable insect cell lines expressing the *Cs*IV protein were used to provide the protein activity in trans, a 5-fold increase in YFP production and viable cells was observed when compared to the control cell line following infection with YFP-BEVS. These data demonstrate the utility of the enhanced BEVS (VE-BEVS) technology for superior over-expression of recombinant proteins in insect cells. In sum, VE-BEVS is an enhancement of the existing BEVS technology that markedly improves protein expression levels while reducing the cost of labor and materials.

### Stable transformation of *Ixodes scapularis* cells for analysis of tick-microbe interactions

T.J. Kurtti, M.J. Herron, R.F. Felsheim, J.T. Mattila, G.D. Baldridge, N.Y. Burkhardt, and U.G. Munderloh.

Department of Entomology, University of Minnesota, St. Paul, MN 55108, USA. Correspondence: kurtt001@umn.edu

Transgenesis and paratransgenesis offer powerful approaches to the analysis of cellular and molecular interactions between ticks and microorganisms. We have developed methods for transfomation and genetic manipulation of an *Ixodes scapularis* cell line (ISE6) that is highly permissive for the cultivation of endosymbiotic and pathogenic bacteria associated with ticks. Line ISE6 was stably transformed using the *Sleeping Beauty* transformation system (Ivics et al. 1997 Cell 91:501) consisting of a reconstructed *Tc1*/*mariner* related transposase and a transposable element. *Sleeping Beauty* transposons in the presence of plasmids expressing *Sleeping Beauty* transposase were used to obtain cells expressing new phenotypes. Marker genes encoded either red fluorescent protein (DsRed2) or neomycin phosphotransferase. After 4 to 6 weeks most cells lost transient expression of the marker genes and stably transformed cells were selected using a neomycin analog, G418. Inverse PCR and sequencing of the integration sites demonstrated that insertions of DsRed2 genes in the cellular genome occurred via the action of the *Sleeping Beauty* transposase. RNAi was used to suppress with expression of the DsRed2 message in transformed cells. We are using live cell fluorescent time-lapse microscopy to examine interactions between transformed *I. scapularis* cells expressing DsRed2 and bacterial pathogens (*Borrelia burgdorferi*) or endosymbionts (*Rickettsia monacensis*) expressing green fluorescent protein. Images of these interactions will be presented. This system has potential for functional genetic analysis of interactions between *I. scapularis* cells and microorganisms. (This research supported by NIH grant AI49424 to UGM)

### Olfactory coding and expression patterns of odorant receptors in the labellum of the malaria vector mosquito *Anopheles gambiae*

H. Kwon, T. Lu, and L.J. Zwiebel

Department of Biological Sciences, Institutes for Chemical Biology and Global Health, Centers for Chemical Biology and Molecular Neuroscience, Vanderbilt University/Vanderbilt Univeristy Medical Center, Nashville, TN 37232, USA. Correspondence: hyung-wook.kwon@Vanderbilt.edu

The ability to sense and discriminate a large collection of chemical and visual cues is central for several behaviors of insects that act as vectors for the pathogens that are responsible for many important human diseases. In particular, olfaction plays a major role in host seeking and selection behaviors of blood feeding female anopheline mosquitoes. This group of mosquitoes includes non-vector species as well as the principal Afrotropical malaria vector species *Anopheles gambiae* whose strong preference for human hosts (anthropophily) is largely responsible for its high vectorial capacity. A long-term objective of our research is centered on an examination of the molecular genetics of the chemosensory system in anopheline and other mosquitoes and its role in determining anthropophilic host preference in malaria vector mosquitoes. Olfactory signal transduction in this mosquito is initiated by odorant receptors (AgORs) that comprise a subfamily of G-protein coupled receptors, which have been and continue to be, the subject of considerable attention as potential targets for novel approaches for the control of malaria. In this study, we present data that provides expression as well as functional information for a set of AgORs that act in the labellum of the proboscis or *An. gambiae* that until now has largely been associated with gustatory signal transduction. In this light, we have examined both non-conventional and conventional members of the AgOR subfamily using molecular, physiological as well as neuroanatomical methods. These studies reveal a novel set of complex olfactory responses and a set of cryptic olfactory receptor neurons in the labellum of *An. gambiae* that is consistent with the hypothesis that the proboscis acts as an accessory olfactory organ that it is linked to a discrete set of antennal lobe glomeruli in this mosquito. Implications of these data regarding the coding of olfactory information will be discussed. It is tempting to speculate that this appendage may detect critical olfactory information derived from potential human hosts at extremely close range that provides a critical component in the penultimate stages of mosquito blood feeding behaviors.

### Herbivore produced elicitors of plant volatiles: Enzymatic biosynthesis and substrate specificity

C.G. Lait and J.H. Tumlinson

Department of Entomology, The Pennsylvania State University, University Park, PA, USA. Correspondence: cgl10@psu.edu

*N*-linolenoyl-L-glutamine is one of several structurally similar fatty acid amide (FAA) elicitors and is synthesized by integral membrane enzyme(s) found in tissues of lepidopterous caterpillar larvae. When *N*-linolenoyl-L-glutamine and other FAA elicitors come into contact with leaves at the location of caterpillar feeding, volatile chemicals are released that in turn attract natural enemies of the caterpillar. We have purified the catalytically active enzyme(s) responsible for *N*-linolenoyl-L-glutamine biosynthesis (FAA synthase) in *Manduca sexta* tissue microsomes and are currently determining its amino acid sequence. FAA elicitors usually consist of hydroxylated or non-hydroxylated 18-carbon polyunsaturated fatty acids coupled with L-glutamine. We demonstrate that microsomal enzyme preparations derived from tissues of *M. sexta*, *Heliothis virescens* and *Helicoverpa zea* can catalyze the biosynthesis of *N*-linolenoyl-L-glutamine and we compared the kinetic parameters for the biosynthesis of *N*-linolenoyl-L-glutamine by midgut tissue microsomes from each species in the presence of the substrates L-glutamine and sodium linolenate. The apparent Km values for coupling of the substrate, sodium linolenate, were 20.7 ± 3.4, 14.3 ± 3.7 and 8.75 ± 0.79 mM and Vmax values were 4.95 ± 0.55, 6.81 ± 1.2 and 2.92 ± 0.14 nmol/min/mg of protein for *M. sexta*, *H. virescens* and *H. zea*, respectively. The Km values for coupling of the substrate, L-glutamine, were 18.9 ± 2.4, 22.3 ± 2.1 and 10.5 ± 2.6 mM and Vmax values were 2.49 ± .41, 3.71 ± 0.50 and 1.78 ± 0.21 nmol/min/mg of protein for *M. sexta*, *H. virescens* and *H. zea*, respectively. The amino acid substrate specificity of FAA synthase from THW was measured by incubating midgut microsomes with a variety of L-amino acids and sodium linolenate in vitro at pH 9.0 and 21°C. We determined that *M. sexta* microsomes could catalyze formation of FAAs with just eight L-amino acids (Asn, Gln, Ser, Thr, Ala, Gly, Met and Val) when sodium linolenate was the other substrate used *in vitro*. The biosynthesis of FAA containing L-glutamine appears to be kinetically favourable when compared to those derived from the other L-amino acids.

### Photoperiodism regulates signaling and structural protein genes in the pea aphid

G. Le Trionnaire^1^, B. Sabater-Muñoz^1^, A. Benedetto^1^, J. Bonhomme^1^, S. Jaubert^1^, N. Leterme-Prunier^1^, T. Cortes^2^, D. Martinez-Torres^2^, J-C Simon^1^ and D. Tagu^1^

^1^INRA Rennes UMR BiO3P, INRA / Agrocampus, BP 35327, 35653 Le Rheu Cedex, France. Correspondence: gael.letrionnaire@rennes.inra.fr

^2^Institut Cavanilles de Biodiversitat i Biologia Evolutiva, Universitat de Valencia, Apartado de Correos 22085, 46071 Valencia, Spain

Aphids are able to change their reproductive mode in response to photoperiodism through the expression of phenotypic plasticity called reproductive polyphenism. Under long-day conditions (spring and summer), aphids reproduce by parthenogenesis. However, the shortening of photoperiod induces a switch to sexual reproduction that occurs by the end of summer. Males and females are thus produced and after mating, over-wintering eggs are laid. The mechanisms of perception of the shortening of photoperiod and its consequences on egg development and morph orientation are misunderstood, but they probably involve early modifications in the neuroendocrine response. Our goal is to identify genes regulated by the shortening of day-length responsible for the reproductive switch. A microarray containing 3000 cDNAs (corresponding to 1700 unigenes) was constructed and used for competitive hybridizations between RNAs extracted from heads of aphids (third instars) reared under long or short photoperiod. After image analysis, normalization and statistical analyses, 98 cDNAs corresponding to 61 unigenes were significantly up- or down-regulated by the shortening of photoperiod. A third of these genes encoded cuticular proteins while 15 % encoded proteins involved in cellular differentiation or signaling. Quantitative PCR experiments were performed on two cuticular protein genes and three signaling protein genes (*wunen*, *Dreg-5* and reeler domain protein) to validate the microarray analysis and to check whether circadian rhythms or molting did not interfere with the photoperiodic response. The putative role of cuticular and signaling proteins in the regulation of reproductive polyphenism will be discussed.

### Proteomics of pupal brains in *Sarcophaga crassipalpis*: First database of diapause-associated proteins

A. Li^1^, A. Popova-Butler^2^, D. H. Dean^1, 2^, and D. L. Denlinger^1^

^1^Department of Entomology, The Ohio State University, Columbus, OH 43210, USA. Correspondence: li.586@osu.edu

^2^Department of Biochemistry, The Ohio State University, Columbus, OH 43210, USA

Most molecular work on insect diapause has focused on the expression of unique diapause transcripts, rather than the protein products. Here we present our first results from a proteomic comparison of diapausing and nondiapausing pupal brains. Proteins extracted from diapausing pupal brains in the flesh fly *Sarcophaga crassipalpis* were separated by two-dimensional gel electrophoresis and compared with those from nondiapausing pupal brains. Unique proteins and proteins expressed at different levels in diapausing and nondiapausing brains were identified by Nano-LC/MS/MS (capillary-liquid chromatography-nanospray tandem mass spectrometry). With this approach we detected 17 unique or upregulated (≥3x) spots, and 11 spots that were downregulated in diapause. Brain proteins present in higher amounts during diapause include HSP70, several small HSPs, Gag protein, cytosolic thioredoxin peroxidase, and keratin. Brain proteins that were less abundant in diapause include phosphoenolpyruvate synthase, fatty acid binding protein, EG0003.7, and endonuclease. Our 2-D proteome maps include many additional unknown proteins. While the mRNAs encoding certain of these proteins (e.g.HSPs) were previously known to be associated with diapause, many of the other proteins were not known to be linked to diapause, thus suggesting that the proteomic approach nicely supplements work done at the transcript level.

### Mechanism of insecticidal action of a basement membrane-degrading protease

Huarong Li^1^, Hailin Tang^1^, Robert L. Harrison^2^, Bryony C. Bonning^1^

^1^Department of Entomology and Program in Toxicology, Iowa State University, Ames, IA 50011, USA. Correspondence: bonning@iastate.edu

^2^USDA, ARS, Beltsville, MD 20705, USA

ScathL is a cathepsin-L like cysteine protease that digests components of the basement membrane during insect metamorphosis. On the basis that basement membranes constitute a barrier to dissemination of baculoviruses within the insect host, a recombinant baculovirus (AcMLF9.ScathL) that expresses ScathL was constructed. AcMLF9.ScathL kills larvae of the tobacco budworm, *Heliothis virescens*, significantly faster than the wild type virus and triggers melanization of larvae shortly before death. To elucidate the mechanism of insecticidal action of ScathL, we are testing five hypotheses; (1) ScathL results in death of the host insect independent of cysteine protease activity: By testing a baculovirus expressing a catalytic site mutant of ScathL, we have shown that cysteine protease activity is required for the insecticidal activity of ScathL. (2) ScathL causes damage to tissues other than the basement membrane: At high concentrations, ScathL results in damage to the gut. However, this damage may be a direct effect of ScathL proteolytic activity, or an indirect effect resulting from lysis of cells underlying disrupted basement membrane. Fragmentation of internal tissues occurs in melanized AcMLF9.ScathL-infected larvae. (3) ScathL damages the basement membrane barrier to virus dissemination allowing more rapid spread or altered tissue tropism of the virus: Damage to the basement membrane that overlies the gut, allows for more rapid movement of budded virus into the hemocoel. (4) ScathL activates the immune response in an unregulated manner by acting directly on prophenoloxidase (PPO): Although ScathL activity was significantly higher in melanized larvae, there were no differences in phenoloxidase activity between AcMLF9.ScathL and control treatments. ScathL does not activate PPO directly *in vitro*. (5) ScathL degrades components of the basement membrane that results in death independent of the immune system: We are using polydnavirus-derived immunosuppressive genes to separate the effects of melanization and the associated production of toxic free radicals, from the potentially lethal impact of basement membrane damage on physiological processes.

### Identification of juvenile hormone response elements (*JHREs*) in *Drosophila melanogaster* genome

Yiping Li and Subba R. Palli

Department of Entomology, University of Kentucky, Lexington, KY 40546, USA. Correspondence: yiping.li@uky.edu

Juvenile hormones (JH) regulate a wide variety of developmental and physiological processes in insects. As a first step in understanding the molecular mechanisms of JH action, we are identifying juvenile hormone response elements (*JHREs*) and proteins that bind to these JHREs. The JH-regulated genes in *Drosophila melanogaster* L57 cells were identified by microarray analysis. The promoter regions of the JH-regulated genes were analyzed and two JHREs, *dmDR4* (degenerate form of *cfJHRE* that was previously identified in the promoter region of JH esterase gene from *Choristoneura fumiferana*) and *dmM1* were identified. Reporter constructs containing the luciferase gene regulated by *dmDR4*- and *dmM1* were constructed. In *Drosophila melanogaster* cells, the *dmDR4*-regulated reporter gene was induced by JH III and the JH III induction was suppressed by 20-hydroxyecdysone (20E). The *dmM1*-regulated reporter gene was induced by JH III, but the JH III induction was not suppressed by 20-E. Using the bioinformatics methods, we searched *Drosophila melanogaster* genome for *dmDR4* and *dmM1* elements and found these elements in the genes belonging to functional groups such as apoptosis, receptor activity, ligand-dependent nuclear receptors, transcription factors and immunity. In addition, some of these *JHRE* containing genes also contain an imperfect palindrome ecdysone response element (*EcRE*, 5′-RGKTCANTGAMCY-3prime;). These initial studies on genome-wide search for JHRE and EcRE point to the complexity and multiple actions of JH and it cross-talk with 20E.

### Localization and function of a putative juvenile hormone esterase binding protein in *Drosophila melanogaster*

Z.Y. Liu, N. Pal, R. Jurenka, and B.C. Bonning

Department of Entomology and Program in Genetics, Iowa State University, Ames, IA 50011-3222, USA. Correspondence: zyliu@iastate.edu

A putative juvenile hormone esterase (JHE) binding protein, P29, was isolated from the tobacco hornworm *Manduca sexta* (J. Biol. Chem. 275(3): 1802-1806). A possible *Drosophila melanogaster* homolog of P29 encoded by CG3776 was identified by sequence alignment, and *in vitro* binding of recombinant *Drosophila* P29 to JHE was confirmed. Three immunoreactive proteins (25kD, 35kD and 50kD) were detected in *Drosophila* larvae, pupae and adults although the predicted size of the protein is 30kD. *Drosophila* P29 is predicted to localize to mitochondria (MitoProt; 93% probability) and has a 5kD N-terminal targeting sequence. Subcellular organelle fractionation and confocal microscopy of *Drosophila* S2 cells confirmed that the immunoreactive 25kD protein is present in mitochondria but not in the cytosol. The 25kD protein can dimerize under *in vitro* conditions. The function of P29 in mitochondria is unknown. By using 5′ RACE we are testing for alternative splicing of the *Drosophila* P29 gene. On the basis that JHE has not been detected in mitochondria, we hypothesize that JHE interacts with the 35 or 50kD proteins which are secreted into the hemolymph of adult flies. P29 may also interact with larval serum protein 1 (LSP1). Phenotypes resulting from hyperexpression of *Drosophila* P29 were as follows: Hyperexpression of P29 during the early larval stages is lethal, while hyperexpression during the third instar results in reduced size of adult flies. Hyperexpression of P29 in adult flies results in hyperactivity; Hyperexpression in females results in reduced fecundity and decreased production of courtship pheromone, cis,cis-7,11-hepta cosadiene. Hyperexpression of P29 in males results in male- male courtship behavior and in decreased production of the aggregation pheromone, cis-vaccenyl acetate. Experiments are underway with null mutant flies to determine whether the 35 and 50kD immunoreactive proteins are encoded by CG3776, and for elucidation of the function of P29.

### The plant bites back: A maize defense cysteine protease suppresses the accumulation of transcripts encoding a major protein of the caterpillar midgut

D. S. Luthe^1^, S. Ozkan^2^, S. Mohan^2^, P.W.K. Ma^3^, and W.P. Williams^4^

^1^Department of Crop and Soil Sciences, Pennsylvania State University, University Park, PA 16802. Correspondence: dsl14@psu.edu

^2^Department of Biochemistry and Molecular Biology, Mississippi State University, Mississippi State, MS 39762

^3^Department of Entomology, Mississippi State University, Mississippi State, MS 39762

^4^USDA-ARS Corn Host Plant Resistance Laboratory, Mississippi State, MS 39762

Previous studies demonstrated that a novel maize cysteine protease, Mir1-CP accumulates in the whorls of caterpillar-resistant maize inbreds in response to insect feeding. Scanning electron microscopy and *in vitro* measurements with purified recombinant Mir1-CP indicated that the enzyme perforated the peritrophic matrix (PM) of *Spodoptera frugiperda* larvae. In this study, we report that the PM protein, Insect Intestinal Mucin (IIM) is one of the main targets of Mir1-CP. Both *in vivo* feeding experiments and *in vitro* treatment of isolated PMs showed that IIM was degraded in the presence of Mir1-CP. In addition, quantitative real-time PCR analysis indicated that IIM transcripts decrease dramatically when larvae were fed on resistant plant material or were force-fed pure Mir1-CP. These results indicate that Mir1-CP damages the PM by attacking IIM, and, either directly or indirectly diminishes the caterpillar’s ability to replenish IIM and replace the PM.

### Evolution of hematophagy in arthropods

Ben J. Mans, Eric Calvo, John F. Andersen, Ivo M.B. Francischetti, Jesus G. Valenzuela and Jose’ M.C. Ribeiro

Laboratory for Malaria and Vector Research, National Institutes of health (NIH/NIAID), Rockville, MD, USA. Correspondence: bmans@mail.nih.gov

Hematophagous behavior evolved independently within arthropods at least six times in different families and orders. In the Insecta hematophagous behavior evolved in the Diptera, Hemiptera, Anoplura and Siphonaptera. It also evolved in ticks within the Arachnida. The fact of independent adaptation to a blood-feeding environment is strikingly observed in the divergent mechanisms that are used to regulate their host’s hemostatic and immune systems. Comparison of the salivary gland transcriptomes from hematophagous arthropods (hematolomes) allows us to gain more insight into the evolution of hematophagous behavior as related to the proteins secreted during feeding. A central feature that emerges when hematolomes are compared is the concept of restricted protein domain usage (RPDU). Essentially, this means that the evolution of novel protein functions that assist the organism in blood-feeding is limited to a subset of protein domains and genes expressed in their salivary glands at the time of their adaptation to a blood-feeding environment. The Diptera for example have a core set of protein domains that is also found in their non-hematophagous neighbors such as Drosophila, although the proteins found in blood-feeding Diptera differ in function from their more ancient counterparts. Protein domains from insect orders more distant to the Diptera have a completely different set of protein domains that is expressed in their glands. In the case of ticks, it would seem as if the two major families (hard and soft ticks) share the same protein domain structure, indicating that the ancestral tick already had a specific protein domain repertoire in its salivary glands. Even so, the functions so far defined for proteins from hard and soft ticks, differ enough in mechanism and protein family classes, that independent adaptation to a blood-feeding lifestyle between the major tick families is still supported. Expansion by gene duplication is a central feature of the core sets of protein families found in all blood-feeding arthropods and is probably the single most important phenomenon that allowed arthropods to evolve the functions necessary to modulate the host’s immune and hemostatic defense mechanisms.This research was supported [in part] by the Intramural Research Program of the NIH, NIAID.

### The molecular basis of host adaptation in cactophilic Drosophila: Molecular evolution of *Glutathione-s-transferase* (*Gst*) genes in *Drosophila mojavensis*

L. M. Matzkin ^1,2^, K. Sweetser ^1^ and T. A. Markow ^1,2^

^1^ Center for Insect Science, University of Arizona, Tucson, AZ, USA

^2^ Department of Ecology and Evolutionary Biology, University of Arizona, Tucson, AZ, USA. Correspondence: lmatzkin@email.arizona.edu

Patterns of transcriptional and sequence variation can be shaped in part by natural selection, especially during the process of adaptation to alternative natural environments. We have previously investigated the role of transcriptional variation in the adaptation of *Drosophila mojavensis* to its hosts and have produced a set of candidate loci that are differentially expressed in response to host shifts. *Drosophila mojavensis* is a cactophilic fly endemic to the north western deserts of North America. This species contains four genetically isolated cactus host races (Baja California, mainland Sonora, Mojave and Catalina Island) each individually specializing in the necrotic tissues of different cactus species (*Stenocereus gummosus*, *S. thurberi*, *Ferocactus cylindraceus* and *Opuntia sp.*, respectively). The necrosis of each cactus species provides each of the resident *D. mojavensis* populations with a distinct chemical environment to which they must adapt. Members of the Glutathione-*S*-transferase gene family have been known to play a role in detoxification in many taxa, including insects. A gene with high homology to the *D. melanogaster* Glutathione-*S*-transferase-D1 (*GstD1*) locus was differentially expressed in a Baja California *D. mojavensis* isofemale line as a response to utilizing an alternative host (*S. thurberi*). In both *D. melanogaster* and in *Anopheles gambiae*, *GstD1* has been implicated in the resistance of these species to the insecticide DDT. We have examined the pattern of sequence variation of the *GstD1* locus from all four *D. mojavensis* populations, *D. arizonae* (its sister species) and *D. navojoa* (outgroup). The data suggest that in the Baja California and Sonora population of *D. mojavensis GstD1* has gone through a period of adaptive amino acid evolution as reflected by the ratio of silent to replacement fixations and polymorphisms. Polarizing these data using *D. navojoa* indicates that the positive selection occurred in the lineage leading to these *D. mojavensis* populations. Further analyses indicate that of the seven amino acid fixations that occurred in the *D. mojavensis* lineage two of them occur in the active site pocket, potentially having a significant affect on substrate specificity and possibly in the adaptation to alternative cactus hosts.

### Regulation of cytochrome P450 genes in *Drosophila melanogaster* by methoprene and the methoprene receptor

Cynthia McDonnell^1^, May R. Berenbaum^1^, and Mary A. Schuler^2^

^1^Dept. of Entomology, University of Illinois Urbana-Champaign, Urbana, Illinois, USA. Correspondence: cmcdonne@uiuc.edu

^2^Dept. of Cell and Structural Biology, University of Illinois Urbana-Champaign, Urbana, Illinois, USA

Resistance to the juvenile hormone analog, methoprene, in *Drosophila melanogaster* has been identified as a target site mutation in a bHLH-PAS protein similar to the mammalian aryl hydrocarbon receptor (AhR), which regulates cytochrome P450 genes in vertebrates. To determine if cytochrome P450 genes are regulated by methoprene and/or the methoprene receptor (Met), transcriptional expression of cytochrome P450 subfamilies and genes was compared between developmental stages, methoprene-sensitive (Oregon-R, Canton-S) and tolerant (Rst(1)JH^1^) strains of *Drosophila melanogaster* and different doses of methoprene. We found that, in third instar Oregon-R larvae, methoprene in the diet was associated with increased expression of CYP6A8, CYP313A, CYP309A1 and the CYP12A subfamily, while CYP310A1, CYP4G15 and the CYP4D subfamily were constitutively expressed and did not respond to methoprene. Expression of the CYP6A, CYP6D, CYP9B, CYP28D and CYP306A1 subfamilies was low in third instar larvae and did not respond to methoprene treatment. In third instar Canton-S larvae, CYP6A8, CYP315A1, CYP4G15 and CYP4D showed low expression and only CYP6A8 showed a slight increase in response to methoprene. In third instar Rst(1)JH^1^ larvae, constitutive expression of CYP12D1, CYP315A1, and CYP4D is not affected by methoprene. And expression of CYP12A is repressed by methoprene. These preliminary analyses demonstrate that methoprene treatment can induce differential expression of P450 transcripts in *D. melanogaster* and that the specific responses depend on strain type and developmental stage. Identifying how cytochrome P450 genes respond to methoprene will help in our understanding of the molecular mechanisms of both insecticide resistance and metamorphosis in insects.

### Iron resources and infection in *Drosophila melanogaster*

E.A. McGraw

School of Integrative Biology, The University of Queensland, Brisbane, QLD 4072, Australia. Correspondence: e.mcgraw@uq.edu.au

The toxicity of free iron is managed in animals by a number of transport and storage molecules. Iron withholding strategies linked to the abundance of these proteins are also an integral part of the insect innate immune response to bacterial infection. To cope with this iron limiting host environment, bacteria have evolved a range of sophisticated means for acquiring iron from their hosts. In the *Drosophila melanogaster* model system we have explored the control of iron resources during infection with both pathogenic and symbiotic bacteria. Our primary goal is to determine the genetic basis of host and pathogen adaptations regarding iron control and their relative costs and benefits for both partners. For our pathogen association we reared *D. melanogaster* using a half sib breeding design to measure the relationship between resistance to infection, expression of innate immune system genes, and tradeoffs in life history traits. The effects were surprisingly sex specific with variation in resistance to infection for males negatively correlated with a composite life history variable. We are currently examining patterns of gene expression to determine whether these tradeoffs are likely due to energetic investments or pleiotropy. In our symbiont association we have used both empirical and genome-wide comparative approaches to characterise the role of iron in *Drosophila:Wolbachia* associations. In contrast to the adversarial nature of the *Drosophila:Pseudomonas* interaction, *Wolbachia* appears to provide a fitness benefit to its host when the insect is reared under a low iron diet. Our results suggest that the bacterium could be provisioning the host with heme or playing a role in iron homeostasis.

### Gene expression and functional studies on a *Drosophila melanogaster* model of Alzheimer’s disease

C. Mee^1^, J. Kidd^1^, L.A. Brown^1^, P. Lescure^1^, D. Baban^1^, R. Page^2^, D. Crowther^2^, D. Lomas^2^, and D.B. Sattelle^1^

^1^MRC Functional Genetics Unit, Department of Physiology, Anatomy and Genetics, Le Gros Clark Building, South Parks Road, Oxford, OX1 3QX. Correspondence: david.sattelle@anat.ox.ac.uk

^2^Cambridge Institute for Medical Research, Wellcome Trust/MRC Building, Addenbrooke’s Hospital, Hills Road, Cambridge, CB2 2XY, UK.

Microarray studies have been carried out on larval brains from transgenic flies (*Drosophila melanogaster*) expressing the human amyloid peptides Aβ1-42 and Aβ1-40 under control of the pan-neuronal driver elavC155. Differentially expressed genes from brains expressing Aβ1-42 and Aβ1-40 were compared. Selection criteria for genes of interest were as follows; the presence of the gene in at least 3 out of 4 arrays, p value 0.01 or less and at least a 2-fold change in gene expression. 251 genes were up-regulated including genes involved in memory and calcium signalling as well as several potassium channel genes, including *shaw*. Of the 345 down-regulated genes, two odorant binding protein genes were present. In a previous study we have shown that chronic exposure of larval cholinergic neurons to Aβ1-42 results in changes in the kinetics of A-type potassium channels*, resulting in a decrease in neuronal activity. Memory loss calcium signalling dysfunction and changes in olfaction have been linked to Alzheimer’s disease pathology. Using the Gal4-UAS system, we have restricted expression of human amyloid peptides to the cholinergic neurons of the fly. Cultured larval cholinergic neurons co-expressing GFP have been used for fura-2-based based calcium imaging and electrophysiology experiments. The increase in intracellular calcium recorded in response to potassium and nicotinic receptor agonists is lower in neurons from transgenic flies expressing Aβ1-42 when compared to driver-only controls. *J. Kidd, L.A. Brown, D.B. Sattelle (2006) J. Neurobiol. 5, 476–487.

### Comparative and Functional Genomics of *Anopheles* Odorant Receptors

Ray Miller, Zhijian (Jake) Tu

Department of Biochemistry, Virginia Tech, Blacksburg, VA 24060. Correspondence: ramiller@vt.edu

The mosquito species *Anopheles gambiae*, *Anopheles stephensi*, and *Anopheles quadriannulatus* show differing levels of preference for human hosts despite being closely or relatively closely related species. This preference is determined in large part by olfactory cues. Therefore G-protein coupled odorant receptors (ORs) may have a critical role in defining this preference. We have used bacteria artificial chromosome (BAC) library screening to isolate, and sequence several OR genes and gene clusters from *An. stephensi* and *An. quadriannulatus*. Using comparative analysis we have located not only changes in coding sequences including insertions in hydrophilic regions, but also many conserved non-coding sequences (CNS) that may have regulatory significance. Currently we are beginning to test the functional significance of these CNSs. Additionally we have used bioinformatics approaches including hidden Markov models to located motifs found in most OR peptides across several insect species. These motifs may be the binding sites that allow OR heterodimer and homodimer formation. They also can serve as a tool for locating OR genes in sequenced or partially sequenced insect genomes given that most OR show little to no conserved sequence identity making search by BLAST less than optimal.

### Using transgenic tools to test gene function in butterfly wing pattern development

Antónia Monteiro, Bin Chen, Diane Ramos, Firdous Kamal, Gary Glaser, and Steven Stoscklagger

Department of Biological Sciences, University at Buffalo, Buffalo, NY 14260. Correspondence: monteiro@buffalo.edu

Eyespots with concentric rings of colored scales are complex structures that appear in a variety of Lepidopteran families. Moths and butterflies share similar gene expression patterns in their eyespot centers, suggesting that a conserved gene network has been triggered multiple times into action. To date, however, none of these genes has been functionally implicated in eyespot formation. We are currently testing whether several candidate transcription factors and ligands, when ectopically expressed, lead to alterations in eyespot patterns. For this purpose we have developed the technique of germ line transformation for the Nymphalid butterfly *Bicyclus anynana*, and developed a new method for ectopically activating genes on the developing wing in a controlled temporal and spatial fashion. This method makes use of precise laser heat-shocks that activate transgenes via a heat-shock promotor.

### Oxygen-sensitive guanylyl cyclases expressed in sensory and central neurons mediate hypoxia avoidance behaviors and chemotaxis

David B. Morton and Anke Vermehren

Department of Integrative Biosciences, Oregon Health & Sciences University, Portland, OR, USA. Correspondence: mortonda@ohsu.edu

Soluble guanylyl cyclases (sGCs) catalyze the synthesis of the intracellular messenger cyclic GMP (cGMP) and can be divided into two sub-families: conventional and atypical sGCs. Conventional sGCs are potently activated by the gaseous messenger, nitric oxide (NO) whereas atypical sGCs are poorly regulated by NO. The *Drosophila* genome contains 3 genes that code for atypical sGCs: *Gyc-88E, Gyc-89Da* and *Gyc-89Db.* We have recently shown that the atypical sGCs in *Drosophila are* regulated by O_2_ rather than NO, showing potent stimulation in the absence of O_2_ and inhibition in the presence of O_2_. To determine the expression patterns of these genes we have generated promoter::GAL4 lines for two of the atypical sGCs and crossed these with fly lines containing red fluorescent protein driven by the UAS promoter. These experiments show expression in a population of sensory and central neurons. The sensory neurons include a small number of cells that innervate the dorsal and terminal organs - larval chemosensory structures that mediate olfactory and gustatory responses. To determine the function of the cGMP pathway in the cells that express the atypical sGCs, we used UAS lines that express a cGMP-specific phosphodiesterase (bPDES) and dsRNA complementary to a cGMP-dependent protein kinase *(dg2* RNAi). Behavioral deficits identified in the progeny of these crosses can be divided into three main groups: disruption of the cGMP pathway in cells that express *Gyc-89Da* have reduced chemotaxis to specific volatile chemicals such as ethyl acetate and cyclohexanone; disruption of the cGMP pathway in *Gyc-89Db* neurons have reduced chemotaxis to sugars and disruption of the cGMP pathway in either *Gyc-89Da* or *Gyc-89Db* neurons causes an inhibition of hypoxia avoidance behavior. These results suggest that the atypical sGCs act as neuronal O_2_ sensors and mediate chemotaxis and behavioral responses to hypoxia. The UAS::bPDE5 and UAS::dg2 RNAi flies we kindly provided by Dr. Shireen Davies, University of Glasgow, UK. This work was supported by NIH grant NS29740.

### An integrated study of several chitin metabolism genes in *Tribolium castaneum*

S. Muthukrishnan^1^, Y. Arakane^1^, Q. Zhu^1^, D. Hogenkamp^1^, C. A. Specht^2^, R. W. Beeman^3^, M. R. Kanost^1^ and K. J. Kramer^1, 3^

^1^Department of Biochemistry, Kansas State University, Manhattan, KS 66506, USA. Correspondence: smk@ksu.edu

^2^Department of Medicine, Boston University, Boston, MA 02118

^3^Grain Marketing and Production Research Center, ARS-USDA, Manhattan, KS 66502.

The recently completed genome sequence of the red flour beetle, *Tribolium castaneum*, was searched for the presence of orthologs of insect genes involved in chitin metabolism. Families of genes encoding chitin synthases, chitinases, N-acetylglucosaminidases, chitin deacetylases and chitin microfibril assembly proteins were identified. Complete or partial cDNA sequences for many of these genes have been determined and these sequences were used to characterize the exon-intron organizations of the corresponding genes. Gene expression studies were carried out in order to assist in choosing appropriate times for carrying out RNA interference (RNAi) experiments designed to assess individual gene function. Injections of dsRNAs for genes corresponding to isozymes of chitin metabolism and chitin microfibril assembly proteins at different developmental stages resulted in selective down-regulation of gene-specific transcripts. Analyses of the phenotypes and/or chitin content of whole bodies, eggs, cuticles and peritrophic membranes of insects following dsRNA injections indicated that there is functional specialization by individual proteins of chitin metabolism. For example, two separate chitin synthases are responsible for synthesis of chitin in the cuticle and the peritrophic membrane, respectively. Different chitinases appear to be required for insect molting at different developmental stages and for normal wing development. Supported in part by National Science Foundation grant IBN-0316963.

### Characterization of secreted protein C002 from salivary glands of the pea aphid, *Acyrthosiphon pisum*

Navdeep S. Mutti^1,2^, Kirk Pappan^2,3^, Khurshida Begum^1^, Loretta K. Pappan^2,4^, Liangjiang Wang^5^, Ming-Shun Chen^6^, Yoonseong Park^1^, John C. Reese^1^, Gerald R. Reeck^2^

^1^ Department of Entomology, Kansas State University, Manhattan, Kansas 66506.

^2^ Department of Biochemistry, Kansas State University, Manhattan, Kansas 66506. Correspondence: navdeep@ksu.edu

^3^ Current address: Department of Biochemistry, Washington University School of Medicine, St. Louis, MO 63110.

^4^ Current address: Department of Pathology, Washington University School of Medicine, St. Louis, MO 63110.

^5^ Department of Biology, Kansas State University, Manhattan, Kansas 66506.

^6^ USDA-ARS and Department of Entomology, Kansas State University, Manhattan, Kansas 66506.

Salivary secretions are a key component of aphid-plant interactions. Aphids’ salivary proteins interact with plant tissues, gaining access to phloem sap and eliciting responses which may benefit the insect. In an effort to isolate and identify key components in salivary secretions, we created a salivary gland cDNA library. Several thousand randomly selected cDNA clones were sequenced. We grouped these sequences into 1769 sets of essentially identical sequences, or clusters. About 30% of the clusters matched clearly to (non-aphid) proteins of known function. Of these, 81% had their top matches to an insect protein. Among our cDNAs, we have identified putative oxido-reductases and hydrolases that may be involved in the insect’s attack on plant tissue. C002 represents an abundant transcript among the genes expressed in the salivary glands. This cDNA encodes a protein that fails to match to proteins outside of aphids and is of unknown function. *In situ* hybridization and immunohistochemistry localized C002 in the same sub-set of cells within the principal salivary gland. C002 protein is detected in fava beans that were exposed to aphids, verifying that C002 protein is a secreted protein. Injection of siC002-RNA causes depletion of C002 transcript levels dramatically over a 3 day period after injection. With a lag of 1 – 2 days, the siC002-RNA injected insects died, on average 8 days before the death of control insects injected with siRNA for green fluorescent protein.

### Development of an RNAi-based community resource for cell culture-based genome-wide screening in the disease vector mosquito, *Aedes aegypti*

K.M. Myles^1^, B. Sobral^2^, Z. Tu^3^, Z.N. Adelman^1^

^1^Department of Entomology, Virginia Tech, Blacksburg, VA, USA. Correspondence: mylesk@vt.edu

^2^Virginia Bioinformatics Institute, Blacksburg,VA, USA

^3^Department of Biochemistry, Virginia Tech, Blacksburg, VA, USA

With the availability of whole-genome sequences for several critical disease vector mosquitoes, the question arises as to what tools are required to best utilize these resources. Currently, microarrays are the leading technology, which can provide a snapshot of gene expression patterns on a global scale. However, microarrays are descriptive in nature and ultimately must be supplemented with functional analysis. This is typically done on a much smaller scale, usually on just a fraction of identified genes. Also, microarray based-screens can only identify genes with strong changes in expression. Genes which are important for a particular pathway or function, but which do not change dramatically in expression levels will continually be missed. Genome-wide screening technology based on function, rather than mRNA levels, represents a powerful alternative to microarray-based screening. Currently, the Drosophila community collectively operates and is served by the Harvard RNAi Screening Center (flyRNAi.org). At this facility, double-stranded RNAs have been synthesized corresponding to every annotated Drosophila gene, giving researchers the abilities to perform an individual assay in a high-throughput 384-well plate format, where in each well RNAi has been induced against a different gene. In this fashion, large groups of genes that are part of the same functional pathway can be identified, including genes important in supporting pathogen replication, signal transduction cascades, survival, etc. While the mosquito community can, and should, make use of this technology in Drosophila, one of the main strengths of this HTS technology comes through the ability to perform assays in primary cells. As cells only need survive for one week to perform a particular screen, biological questions could be addressed on a genome-wide scale in important mosquito-specific tissues such as the midgut or salivary glands. In collaboration with the Harvard RNAi Screening Center, and if there is sufficient interest and excitement, we aim to establish a Mosquito RNAi Screening Center which would serve the entire vector community.

### Functional characterization of the promoter of the vitellogenin gene, *AsVg1*, of the malaria vector, *Anopheles stephensi*

Xavier Nirmala^1,^*,Osvaldo Marinotti^1^,Juan Miguel Sandoval^1^,Sophea Phin^1^,Surendra Gakhar^2^,Nijole Jasinskiene^1^ and Anthony A. James^1, 3^

^1^Department of Molecular Biology and Biochemistry, University of California, Irvine, CA 92697, USA. Correspondence: omarinot@uci.edu

^2^Department of Biosciences, Maharshi Dayanand University, Rohtak, India

^3^Department of Microbiology and Molecular Genetics, University of California, Irvine, CA 92697, USA

*Present address: USDA-ARS, 1700 SW 23^rd^ Drive, Gainesville, Florida - 32608, USA

Some genetic strategies for controlling transmission of mosquito-borne diseases call for the introgression of antipathogen effector genes into vector populations. Endogenous mosquito promoter and other *cis*-acting DNA sequences are needed to direct the expression of effector molecules. In order to test the efficacy of a vitellogenin-encoding gene promoter to drive tissue-, stage- and sex-specific expression of exogenous genes, one of the *Anopheles stephensi* vitellogenin genes, *Asvg1*, was cloned, and its full-length transcript, as well as 850 nucleotides adjacent to its 5′-end, were sequenced and characterized. The expression of the gene is restricted to the fat bodies of blood-fed females, and the amino acid sequence of the deduced protein is > 85% identical to those of other anopheline vitellogenins. These characteristics support the conclusion that *AsVg1* is a vitellogenin-encoding gene. Functional analyses of the *Asvg1* putative *cis*-regulatory sequence were performed by generating transgenic mosquitoes. The results showed that the 850 nucleotides immediately adjacent to the 5′-end of the gene and the 3′-end untranslated region are sufficient to direct sex-, stage- and tissue-specific expression of a reporter gene. These data indicate that the *AsVg1* promoter is a good candidate for controlling the expression of anti-pathogen effector molecules in this malaria vector mosquito.

### Use of missense proteasome subunits for conditional lethality in the tephritid fruit flies *Anastrepha suspensa* and *Ceratitis capitata*

X. Nirmala, G.J. Zimowska and A.M. Handler

USDA-ARS-CMAVE, 1700 23^rd^ Drive, Gainesville, FL 32608, USA. Correspondence: handler@nersp.nerdc.ufl.edu

Proteasomes play a critical role in eukaryote development by regulating protein degradation (see Covi et al., 1999). Ubiquinated proteins undergo proteolysis in a multi-subunit complex known as the 26S proteasome, which is comprised of a 20S core and 19S regulatory complexes. Mis-sense mutations in the 20S subunits lead to the production of dominant temperature-sensitive (DTS) “poison subunits” or antimorphs that disrupt proteasome function. DTS5 and DTS7 are two such mutations identified originally in *Drosophila melanogaster* that result in late larval or pupal lethality at 29°C. To study the potential of these genes to control the populations of tephritid fruit fly pests by conditional lethality, the *D. melanogaster* DTS5 mutation was genetically transformed into the medfly, *Ceratitis capitata*, and the caribfly, *Anastrepha suspensa*. When reared at 30°C transformed medflies homozygous for the transgene exhibited 90–95% late larval or pupal lethality, with lower lethality levels found in transformed caribflies (Handler, unpublished). To enhance the temperature sensitive lethal effect we propose the use of native mutated proteasome genes in these species. The proteasome β2 subunit corresponding to DTS7 was isolated from *A. suspensa* pupal cDNA library by gene amplification. Degenerate primers designed from the most conserved regions of insect DTS7 were used in combination with 5′ and 3′ adaptors. Subsequently DTS7 genomic DNA was isolated by gene amplification using gene specific primers. The *A. suspensa* DTS7 (AsDTS7) coding region contains 843 nts that potentially encodes a 281 amino acid protein. Residues 40 to 224 comprise the proteasome beta domain conserved among eukaryotes. At the amino acid level AsDTS7 shares 85.7% to *D. melanogaster* proteasome subunit. AsDTS7 transcript contains 1024 bp that is interrupted in the genome by 3 short introns ranging in size from 57–66 nts. Northern blot analysis indicates the presence of AsDTS7 transcript from embryonic through the adult stages with quantitative variations during development, with an apparent maternal contribution to embryos. *In vitro* mutagenesis will be used to introduce the missense mutation in AsDTS7 that corresponds to the DTS7 mutation in *D. melanogaster*.

### The role of RNA interference in arbovirus-vector interactions

Ken E. Olson

Arthropod-borne and Infectious Diseases Laboratory, Foothills Research Campus, 3185 Rampart Road, Mail Delivery 1692, Department of Microbiology, Immunology and Pathology, Colorado State University, Fort Collins, CO 80523. Correspondence: kenneth.olson@colostate.edu

Since the discovery of RNA silencing, we have hypothesized that RNAi plays a critical role in arbovirus-mosquito interactions and is a frontline defense that mosquitoes have to control RNA virus invasion. RNAi is triggered by dsRNA and destroys any RNA with significant stretches of sequence identity. RNAi is induced by viruses which form dsRNA intermediates as they replicate in permissive cells. The RNAi pathway in drosophila (and mosquitoes) has two branches: the siRNA branch and the micro-RNA (miRNA) branch. The siRNA branch recognizes long dsRNAs and mediates virus control and the miRNA branch recognizes shorter dsRNAs, does not require exact sequence matches in the target site, and is critical for insect development and gene regulation. Two recent papers have now shown that successful infection and killing of drosophila with the insect nodavirus, flock house virus, is dependent on the virus controlling the siRNA branch of the RNAi pathway (Galiana-Arnoux et al., 2006; Wang et al., 2006). In addition, drosophila with a knockout mutation for the gene (*Dcr2*) encoding Dicer-2, a key component gene of the siRNA branch, showed enhanced susceptibility and pathology to infection by flock house virus, cricket paralysis and drosophila C viruses (*Dicistroviridae*) and the arbovirus, Sindbis (Togaviridae). The importance of RNAi in controlling virus infections extends beyond drosophila models. Many of the genes (*dcr2, Ago2, R2D2*) associated with the siRNA branch of RNAi have now been found in *Anopheles gambiae* and *Aedes aegypti* genomes. Several research groups, including our own, have shown these mosquitoes can efficiently detect dsRNAs and silence any mRNA of appropriate sequence identity to the dsRNA. Although we currently cannot generate *dcr2* null mutations in mosquitoes, we can RNA silence critical components of the RNAi pathway to in effect disrupt RNAi. As examples, the RNAi pathway in *Anopheles gambiae* was silenced by injecting dsRNA derived from exon sequences of the *A. gambiae argonaute2* (*AgAgo2*) and *Dcr2* gene (*AgDcr2*). If the RNAi pathway influences viral invasion silencing *AgAgo2* or *AgDcr2* expression would make *A. gambiae* more permissive to O’nyong-nyong virus (ONNV) virus infection. Indeed by altering the RNAi pathway, mosquitoes became more susceptible to ONNV virus and virus spread throughout the mosquito faster than mosquitoes with a non-silenced RNAi pathway. These observations extend to *A. aegypti*, a vector of several arboviruses with medical significance. In transgenic *A. aegypti* we have induced RNAi in the midgut to dengue virus type 2 (DENV2; *Flaviviridae*) by transcribing a dengue derived, inverted-repeat RNA (dsRNA) from the midgut-specific *carboxypeptidase* promoter following ingestion of a viremic blood meal. These transgenic mosquitoes were highly resistant to midgut infection and virus dissemination and transmission of the parental virus. The presence of DENV-2-derived siRNAs in RNA extracts from midguts of the transgenics and the loss of the resistance phenotype when the RNAi pathway was interrupted by silencing *AaAgo2* proved that DENV-2 resistance phenotype was caused by the RNAi response. Therefore the anti-viral branch of the RNAi pathway is functional in vector species. The question remains as to how arboviruses have adapted to the RNAi pathway so that they can be successfully transmitted and maintained in nature. If we can understand how arboviruses are tipping the balance of power in their favor, we might be able to successfully intervene in virus transmission.

### Digestive proteases in tenebrionid insects

Brenda Oppert^1^, Elena N. Elpidina^2^, Konstantin S. Vinokurov^2^, Marce Lorenzen^1^, Ming-Shun Chen^1^, Sheila Prabhakar^3^, C. Michael Smith^3^, and Richard Beeman^1^

^1^USDA ARS Grain Marketing and Production Research Center, Manhattan, KS 66502 USA. Correspondence: bso@ksu.edu

^2^A. N. Belozersky Institute of Physico-Chemical Biology, Moscow State University, Russia

^3^Department of Entomology, Kansas State University, Manhattan, KS 66506 USA.

Protein digestion in coleopteran pests is a frequent target for biological insecticides, such as plant inhibitors. However, compensatory responses to protease inhibitors by coleopteran pests often compromise the efficacy of this approach, and thus a thorough understanding of coleopteran digestive proteases is needed. We have studied protein digestion in the yellow mealworm, *Tenebrio molitor*, at both the protein and gene levels. A comprehensive biochemical study suggested that, under normal dietary conditions, larvae use at least six cysteine and nine serine proteases to digest protein. In an EST study, cDNAs were obtained that encoded cysteine, serine, and metallo proteases. Two biochemically-characterized serine proteases correlated to cDNA sequences by N-terminal sequence and mass spectral analyses. Proteolytic activity of these enzymes is regulated in part by spatial compartmentalization and distinct pH environments in the gut. Both biochemical and EST data indicate that cysteine proteases prevail in the more acidic anterior midgut, while serine proteases are more abundant in the alkaline posterior midgut. These data provide the basis for a study of responses to protease inhibitors in *T. molitor* larvae. Although the number of total protease genes in *T. molitor* is unknown, inferences were made through the annotation of proteinase genes in a tenebrionid with a sequenced genome, *Tribolium castaneum*. Biochemcial studies in *T. castaneum* have indicated that larvae digest protein primarily through the action of cysteine proteases. The comparison of cloned *T. molitor* proteases suggests that, although digestive proteases are highly conserved in the two species, there is divergence in protease gene expression.

### Juvenile hormone analog methoprene blocks midgut metamorphosis by modulating ecdysteroid action

Parthasarathy R., Wu Yu, Hua Bai and Subba Reddy Palli

Department of Entomology, University of Kentucky, KY, USA. Correspondence: rpalli@uky.edu

In holometabolous insects such as the mosquito, *Aedes aegypti*, the midgut undergoes remodeling during metamorphosis. Insect metamorphosis is regulated by several hormones including juvenile hormone (JH) and 20-hydroxyecdysone (20E). The JH analog, methoprene, is widely used to control mosquitoes, but its mode of action is not known. The molecular mode of action of methoprene on midgut remodeling was investigated by studying nuclear stained whole mounts and cross-sections of midguts and by monitoring the mRNA levels of genes involved in 20E action in methoprene-treated and untreated *Ae. aegypti*. Most of the larvae treated with methoprene died during the pupal stage. In *Ae. aegypti* larvae, the programmed cell death (PCD) of larval midgut cells and the proliferation and differentiation of imaginal diploid cells were initiated at about 36 hr after ecdysis to the fourth instar larval stage (AEFL). The destruction of larval midgut epithelium and formation of pupal/adult midgut were completed by 12 hr after pupal ecdysis. In methoprene-treated larvae, the proliferation and differentiation of diploid cells was initiated at 36 hr AEFL and programmed cell death was initiated later after ecdysis into the pupal stage, but the terminal events that occur for its completion during pupal stage were blocked. As a result, pupae that developed from methoprene-treated larvae contained two midgut epithelial layers until they died during the pupal stage. Real-time PCR analyses showed that methoprene affected midgut remodeling by modulating the expression of ecdysone receptor B, ultraspiracle A, broad complex, E93, FTZ-F1, DRONC and DRICE, the genes that are shown to play key roles in 20E action and programmed cell death. We conclude that methoprene acts on *Ae. aegypti* by interfering with the expression of genes involved in 20E action resulting in a block in midgut remodeling and death during the pupal stage.

### A mosquito lysozyme serves as a positive regulator for development of *Plasmodium* on the midgut

Susan M Paskewitz, Bin Li, and Lei Shi

Department of Entomology, University of Wisconsin, Madison, WI, USA. Correspondence: paskewit@entomology.wisc.edu

Insect lysozymes are well known effector molecules in immune responses against bacteria. In *Anopheles gambiae*, there are 8 c-type lysozymes. Gene silencing demonstrated that one of these, Lys c-1, positively regulates the number of *Plasmodium berghei* oocysts that develop on the midgut. In the absence of Lys c-1, oocyst (but not ookinetes) numbers are reduced, as is prevalence of infection within a cohort of mosquitoes. Results from immunohistochemistry suggest that Lys c-1 binds to the oocyst stage, perhaps forming part of the oocyst capsule wall. Lys c-1 may represent a new target for development of transmission blocking or transgenic mosquito strategies.

### Cell cycle regulation during overwintering in the flesh fly *Sarcophaga crassipalpis*

Savvas Pavlides, Kenneth Weir and Steven P. Tammariello

Department of Biological Sciences, Binghamton University (SUNY), Binghamton, NY 13902. Correspondence: savvas74@hotmail.com

During pupal diapause in the flesh fly, *Sarcophaga crassipalpis,* the cells of the brain are arrested in the G1 phase of the cell cycle. Following artificial termination of diapause by the topical application of hexane, the cells re-enter the cell cycle within 12 hours. Alternatively, during post-diapause quiescence the cells remain arrested until temperatures increase sufficiently to trigger post-diapause cell proliferation, ultimately leading to adult development. In order to define the molecular control of this cell cycle arrest, we studied the expression patterns of three genes that encode for proteins that regulate the G1 to S phase boundary in eukaryotic cells. Semi-quantitative RT-PCR was used to examine the expression patterns of *cyclin e, E2F1* and *proliferating cell nuclear antigen* (*pcna*) using mRNA samples extracted from non-diapausing pupae, pre-diapausing pupae, early-, mid- and late-diapausing pupae, post-diapausing pupae and pharate adults. All three genes are down-regulated during diapause, and transcript levels remain low until diapause termination. Transcripts from all three genes significantly increase by 12 hours after diapause termination. Western blot analysis and immunohistochemistry reveal that PCNA protein levels are unaffected during diapause, while Cyclin E and E2F1 are differentially generated at the protein level during the diapause program in flesh flies. These data provide evidence suggesting that the transcriptional control of these three genes may be important in the negative regulation of the cell cycle during flesh fly overwintering.

### Caterpillar vs. plant: The roles of saliva and trichomes in herbivory

Michelle Peiffer and Gary Felton

Department of Entomology, Penn State University, University Park PA, USA. Correspondence: mlk101@psu.edu

Induction of glandular trichomes on tomato leaves has been shown to increase resistance to herbivory. This study examined the role of caterpillar saliva in trichome induction as well as the role trichomes play in the plant defense response. When *H. zea* larvae were allowed to feed on the youngest leaf of 4 node tomato plants, after 2 weeks, new leaves had 85.8 trichomes per square inch disc, compared to 59.3 on untreated plants. However, if the larval spinnerets were cauterized to stop the caterpillar from secreting saliva, there were 125.4 trichomes per disc. When plants were wounded by punching holes in the leaves, new leaves had higher trichome densities than unwounded controls. However, if saliva collected from H. zea was applied to the wound site, trichome density on new leaves was comparable to unwounded control plants. The protective role of trichomes has traditionally been attributed to polyphenol oxidase, but we have shown that many signaling genes are also expressed in glandular trichomes. Reverse transcriptase pcr performed on isolated trichomes detected prosystemin and 4 other genes from the jasmonic acid pathway. Disrupting the trichomes by rubbing the leaf results in increased wound inducible protease inhibitor II in the leaf. These data suggest that caterpillar saliva plays an important role in herbivory, by preventing trichome induction, which is one method plants use to protect themselves from herbivory.

### Development and characterization of European corn borer resistance to the Cry1F toxin from *Bacillus thuringiensis*

E.J.G. Pereira^1^, B. A. Lang^2^, M. Zhuang^2^, N. P. Storer^2^, and B. D. Siegfried^1^

^1^Department of Entomology, University of Nebraska, Lincoln, NE 68583. Correspondence: eliseu@unlserve.unl.edu

^2^Dow AgroSciences LLC, 9330 Zionsville Rd., Indianapolis, IN 46268

Evolution M. Zhuang of resistance by insect-pests is the greatest threat to the continued success of *Bacillus thuringiensis* toxins used in sprays or in transgenic crop plants such as maize expressing the Cry1F toxin for control of lepidopteran pests. Availability of laboratory-selected insect strains allows determination of biochemical mechanisms of resistance that can evolve as well as identification of genes involved. A strain of European corn borer, *Ostrinia nubilalis* (Hübner) (Lepidoptera: Crambidae), obtained from field collections throughout the U.S. Corn Belt in 1996 was selected in the laboratory for resistance to Cry1F by exposure to the toxin incorporated into artificial diet. The selected strain developed more than 3000-fold resistance to Cry1F, yet it was as susceptible to Cry1Ab and Cry9C as the unselected control strain. Only a low level of cross resistance (7-fold) to Cry1Ac was observed. Dose-response of reciprocal parental crosses indicated that the resistance is autosomal and recessive. Backcross of the F_1_ generation with the selected strain revealed that a single locus or a set of tightly linked loci is responsible for the resistance. Analyses using ligand-toxin immunoblotting and Surface Plasmon Resonance to measure Cry1F binding to brush border membrane vesicles of midgut epithelia from susceptible and resistant larvae showed no reduced binding associated with resistance. Additionally, expression of two putative Cry1-receptor proteins, cadherin and aminopeptidase, was similar in the control and selected strains. Moreover, no altered activity of luminal gut proteases and proteolytic processing of the toxin were observed in the resistant strain. Although the resistance mechanism remains uncertain, there is no direct evidence that altered binding and proteolytic processing of toxin are involved. The resistance mechanism in this Cry1F-selected strain of corn borer appears to be specific and maybe distinct from previously identified resistance mechanisms in Lepidoptera. Follow-up studies are ongoing to isolate the resistance gene(s) and develop molecular probes for monitoring evolution of resistance in the field.

### Trafficking of Fragile X protein and associated mRNAs in neural development

Marianna Pintér ^1^, Patty Estes ^1^ and Daniela C. Zarnescu ^1,2,3^

^1^Department of Molecular and Cell Biology, University of Arizona, Tucson AZ 85721. Correspondence: arianna@u.arizona.edu

^2^Committee on Neuroscience, University of Arizona, Tucson AZ 85721

^3^Interdisciplinary Program for Genetics, University of Arizona, Tucson AZ 85721

Fragile X syndrome is the most common form of inherited mental retardation and is caused by loss of function for *Fmr1 gene*. Phenotypes include an IQ below 70, facial dysmorphia, attention deficit and hyperactivity disorders, sleep disturbances, autism and macroorchidism, and patients have elongated neocortical dendritic spines. While mice and humans contain a three-member *Fmr1* gene family *Drosophila* has a single gene ortholog: *dFmr1. dFmr1* mutants are viable and exhibit defects in synapse morphology and function and in neuronal arborization and circadian rhythm. To identify novel genes that participate in *dFmr1* function we performed a genetic screen for dominant modifiers of retinal overexpression of dFmr1 in *Drosophila* (*sev:dFmr1*). We found that *lethal giant larvae* (*lgl*) is a major regulator of *dFmr1* function. *lgl* is essential for viability and is required for dorsal closure, neuroblast delamination, wing development and oogenesis. Lgl forms molecular scaffolds with itself, non-muscle myosin and the PAR complex and is phosphorylated by atypical-PKC-zeta (aPKC-zeta) in flies and mice. Lgl regulates docking and fusion of post-Golgi vesicles with the plasma membrane through SNARE complex association. In summary, Lgl is believed to contribute to cellular asymmetry via its association with: i) cell junctional complexes and ii) the cytoplasmic transport machinery. Our data shows that *lgl* functions upstream or in parallel to *dFmr1* at the neuromuscular junctions and in oogenesis. Lgl and dFmr1 proteins partially colocalize in granular structures and form a complex in vivo, which includes a small number of target mRNAs. FMRP shuttles between the nucleus and the cytoplasm and associates with RNA via its two KH domains and an RGG box. Our data suggests that Lgl is involved in the polarized delivery of dFmr1 and a subset of associated mRNAs by regulating their sorting, transport and/or anchoring. We are currently testing these hypotheses by taking a combined cell biological, biochemical and genetic approach. Our long term goal is to dissect the molecular machinery which controls the function(s) of Fragile X protein in synaptic development and plasticity. This work was supported by FRAXA and NIH for NRSA (NS046880) to DCZ.

### Antiviral response in *Heliothis virescens*

H.J.R. Popham

USDA ARS Biological Control of Insects Research Laboratory, Columbia, MO 65203. Correspondence: pophamh@missouri.edu

Lepidopteran larvae are known to resist baculovirus infection by the selective apoptosis of infected midgut epithelial cells, and by the sloughing off of infected cells from the midgut. Once the infection breaches the midgut epithelial barrier and propagates from infective foci to the hemocoel however, there are few known mechanisms to account for the resistance and clearance of infection observed in some virus/host combinations. Utilizing an *in vitro* assay, *Heliothis virescens* larval plasma was found to contain high levels of an antiviral activity against *Helicoverpa zea* single nucleopolyhedrovirus (HzSNPV) budded virus. The innate factor responsible for the virucidal effect was identified as phenoloxidase. To elucidate the contributions of phenoloxidase to the innate immune response against baculovirus infection, specific inhibitors were employed. *In vitro* the general inhibitors of melanization (N-acetyl cysteine, ascorbate and glutathione), and specific inhibitors of phenoloxidase (phenylthiourea, and Kojic acid), completely blocked virucidal activity up to the level seen in controls. Addition of the enzyme catalase to plasma did not affect virucidal activity; however addition of superoxide dismutase exhibited a modest inhibitory effect. Inhibitors of nitric oxide synthase activity did not affect virucidal activity. Beyond innate virucidal activity, proteins induced by viral infection were also studied. Using two dimensional difference gel electrophoresis (DIGE, Amersham), protein expression was compared between plasma from uninfected and infected larvae. Preliminary results of this study will be presented.

### Evolution of warning coloration in *Papilio* larvae

K. L. Prudic^1^ and J. C. Oliver^2^

^1^Ecology and Evolutionary Biology, University of Arizona. Correspondence: klprudic@email.arizona.edu

^2^Interdisciplinary Program in Insect Science, University of Arizona

Warning, or aposematic, coloration is a visual signal minimizing contact between predator and unprofitable prey. The conditions favoring the evolution of aposematic coloration remain largely unidentified. Recent work suggests that diet specialization may play a role in facilitating the evolution and persistence of warning coloration. Using a phylogenetic approach, we investigated the evolution of larval warning coloration in the genus *Papilio* (Lepidoptera: Papilionidae). Our results indicate there are at least four independent origins of aposematic larval coloration within *Papilio*. Parametric bootstrapping results reject the hypotheses of one, two, and three origins of aposematic larvae. Controlling for phylogenetic relatedness among *Papilio* taxa, we found no evidence supporting the hypothesis that diet specialization facilitated the origin of aposematic larvae. However, there was a significant relationship between host plant growth form and the evolution of aposematic larvae. Specifically, *Papilio* lineages feeding on herbaceous plants were more likely to evolve aposematic larvae than were lineages feeding only on shrubs and trees. These results demonstrate that factors other than diet specialization, such as the visual environment of predator-prey interactions, may play a large role in the initial evolution and persistence of aposematic coloration. Future studies should consider environmental context in determining the forces responsible for the evolution of *Papilio* warning coloration.

### Resistance levels and mechanism of resistance in *Plutella xylostella* to certain commonly used insecticides

S.V.S.Raju^1^, U. K. Bar^1^, Shailendra Kumar^1^ and Uma Shankar^2^

^1^Department of Entomology, Institute of Agricultural Sciences, Banaras Hindu University, Varanasi, 221005, India. Correspondence: svsraju_bhu@yahoo.com

^2^Division of Entomology, Faculty of Agriculture, SKUAST, Udhaywalla, Jammu, 180002, India

Resistance to carbosulfan, cartap hydrochloride, fenvalerate, monocrotophos and quinalphos in field population of diamondback moth (DBM*), Plutella xylostella* L., was assessed by discriminatory dose (LC_99_) technique. The survival percentage showed that the DBM larval population was resistant to all the test insecticides but the degree of resistance varied. The extent of resistance was more to quinalphos (86.4 ± 2.1%) and less to carbosulfan (9.61 ± 1.8%) and cartap hydrochloride (18.5 ± 2.4%). Further, mechanism of resistance was studied with the help of synergists at a concentration of 50ppm each and adopting larval dip assay. Suppression of fenvalerate resistance by PBO (piperonyl butoxide), PP (Propargyloxyphthalmide) and profenophos was 20.6 ± 4.1%, 22.3 ± 4.6% and 29.4 ± 5.2%, respectively. Supression of quinalphos resistance by TPP (triphenyl phosphate) and DEF (S,S,S-tributyl phosphorotrithioate) was marginal with survival percentage of DBM larvae was 75.8 ± 4.9% and 63.1 ± 5.7%, respectively. The oxidative metabolisers like PBO and PP of microsomal oxidases were found to synergise fenvalerate effectively showing MFO mediation may be one of the important mechanisms involved in fenvalerate resistance. It is concluded that understanding mechanism of resistance is an indispensable pre-requisite in designing a resistant pest management strategy.

### A seminal protease processes reproductive molecules in *Drosophila melanogaster*

K. Ravi Ram, L.K. Sirot, and M.F. Wolfner*

Department of Molecular Biology and Genetics, Cornell University, Ithaca NY 14853, USA. Correspondence: mfw5@cornell.edu

Males provide females with proteins that affect females’ reproductive output. Sometimes these proteins are made in an immature form in males and may either be activated or inactivated in the female by processing proteins that accompany them. Proteases and protease inhibitors are found in the seminal fluid of many animals, and are thought to play important roles in fertilization and fertility. In *Drosophila melanogaster*, about 25% of the 55 seminal proteins thus far identified from the male’s accessory gland are predicted proteolysis regulators: 6 predicted proteases and 7 known or predicted protease inhibitors. Two potential targets of these proteolysis regulators are the ovulation hormone ovulin (Acp26Aa) and the sperm-storage protein Acp36DE, which are both cleaved in mated female flies. Two of ovulin’s cleavage products are bioactive, and ovulin’s cleavage is known to require male as well as female contributions. To identify male-derived proteolysis-regulators that cleave ovulin and/or Acp36DE, we used RNA interference (RNAi) to generate knock-down males that lacked activity of individual seminal proteases or protease inhibitors. Biochemical analyses of the mates of these knock-down males identified a seminal protease whose activity is necessary to process both ovulin and Acp36DE within mated females. In addition, we showed that processing of seminal proteins occurs in a step-wise manner in *Drosophila*: it begins in the male, when male secretions are mixed, and further processing then occurs in the mated female.

### Multiple origins of metabolic insecticide resistance in *Anopheles* and *Aedes* mosquitoes

Hilary Ranson, Pie Mueller, Clare Strode and Nongkran Lumjuan

Vector Research Group, Liverpool School of Tropical Medicine, Liverpool, L3 5QA, UK. Correspondence: hranson@liverpool.ac.uk

Insecticide resistance is a major threat to sustainable mosquito control. An understanding of the mechanisms conferring this trait facilitates the management of resistance and may eventually lead to novel strategies to restore the efficacy of the insecticide. Increased production of the enzymes catalysing the detoxification of insecticides is one of the most significant causes of insecticide resistance. Three enzyme families have been implicated in insecticide metabolism, the glutathione transferases, carboxylesterase and cytochrome P450s. We have produced two small scale microarrays containing unique probes for each member of these three supergene families, one for the malaria vector *Anopheles gambiae* and a second for the major vector of dengue, *Aedes aegypti*. We are using these arrays to compare the expression of each of the 200 or so detoxification genes in insecticide susceptible and resistant populations of mosquitoes to identify those genes whose expression correlates with the resistance phenotype. These candidate insecticide resistance genes are then expressed *in vitro* and the ability of the recombinant proteins to metabolise insecticides determined. Using this approach we have shown that metabolic resistance to pyrethroids has arisen independently in East and West African populations of *Anopheles gambiae* and analogies can be drawn with the multiple origins of target site resistance to pyrethroid insecticides that have been reported in this species. Preliminary data also suggests independent origins of metabolic resistance to DDT in *Aedes aegypti* from two different continents. The implications of these findings for resistance management strategies will be discussed.

### Upregulation of stress associated genes in the overwintering stages of the antarctic midge, *Belgica Antarctica*

J. P. Rinehart^1^, G. Lopez-Martinez^2^, S.A.L. Hayward^3^, L. H. Sandro^4^, R.E. Lee^4^, and D.L. Denlinger^2^

^1^USDA-ARS Red River Station, Fargo, ND, USA. Correspondence: rineharj@fargo.ars.usda.gov

^2^Dept of Entomology, Ohio State University, Columbus, OH, USA

^3^School of Biological Sciences, Liverpool University, Liverpool, UK

^4^Dept of Zoology, Miami University, Oxford, OH, USA

We have investigated the molecular basis of stress resistance in the midge *Belgica antarctica*, the largest known free-living animal to adapt to a terrestrial existence on the Antarctic continent. Prevalent in specific locations throughout the Antarctic peninsula, this insect has a two-year life cycle and can overwinter in any of its four larval instars. During the austral summer, adults emerge and are present for a brief 1–2 week period. Our research team has investigated the expression of heat shock proteins (hsps) with respect to overwintering and has used suppressive subtractive hybridization to identify additional stress associated genes that might be involved. A comparison of larvae and adults has revealed that several hsps (a small hsp, hsp70, and hsp90) are all strongly upregulated in the larvae at all times, but in the adults these hsps are upregulated only in response to extreme temperatures. The larvae do not further upregulate the expression of these genes in response to thermal or desiccative stress. Suppressive subtractive hybridization and northern blot hybridization has revealed a similar expression pattern for several other stress associated genes. Superoxide dismutase, metallothionein and a gene with similarity to vaculor ATPase all exhibit upregulation in larvae. We have also identified other transcripts including actin and a zinc finger protein of unknown function which exhibit a similar pattern of expression and may be implicated in stress adaptation. Further studies on the expression of these genes are underway to elucidate the molecular mechanisms that have allowed this insect to adapt to the harsh conditions of the Antarctic continent.

### Extraordinary thermotolerance and Hsp70 Induction temperatures in the honey bee, *Apis mellifera*

S. P. Roberts and M. M. Elekonich

Department of Biological Sciences, University of Nevada Las Vegas, Las Vegas, NV 89154-4004. Correspondence: sroberts@ccmail.nevada.edu

Honey bees spend the first 2–3 weeks of their adult lives working inside the constant environment of the hive which they maintain at 33–35 °C by a variety of behavioral and physiological thermoregulatory mechanisms. At about 3 weeks of age workers frequently leave the hive as foragers to gather pollen and nectar for the colony. This period of their lives is marked by periodic episodes of extreme body temperature (> 40 °C) resulting from both environmental and metabolic heat loads. In this study we measured mortality and expression of Hsp70 family proteins in heads and flight muscles of surviving bees following heat treatments varying from 33 to 50 °C and from 0.5 to 4 hours. Levels of a subset of encoding genes, *hsp70* and *hsc70–4*, were also measured following 1-hour heat treatments across the same range of temperatures. There was little or no mortality in bees held at 33 and 42 °C, while the time prior to 50% mortality at 46, 48 and 50 °C was 3.25, 1.75 and 1.25 hours, respectively. The threshold induction temperature for Hsp70 expression and elevated *hsp70*/*hsc70–4* activity in brains was 46 °C, although heat-induction of Hsp70 and elevated *hsp70*/*hsc70–4* in flight muscles were undetectable. Furthermore, the maximum induction of brain Hsp70 expression and *hsp70*/*hsc70–4* activity occurred only after longer exposures, yet was very modest at levels only 2–3 times baseline values (*vs*. approximately 1000-fold for ectothermic insects such as *Drosophila*). These data illustrate that honey bee tissues, especially flight muscles, are extremely thermotolerant and suggest that the thermal kinetics of Hsp70 induction and transcription of their encoding genes have co-evolved with endothermy.

### Development of a novel all natural tick and insect repellent, BioUD, as a DEET replacement and for use on cotton fabric

R. Michael Roe^1^, Kevin V. Donohue^1^, Allen Jones^2^, Matthew Vanderherchen^1^, Charles S. Apperson^1^, Matthew Isherwood^3^, and Russell Linderman^3^

^1^Department of Entomology, Campus Box 7647, North Carolina State University, Raleigh, NC 27695, USA. Correspondence: michael_roe@ncsu.edu

^2^HOMS, LLC, P.O. Box 724, Clayton, NC 27520, USA

^3^Department of Chemistry, Campus Box 8204, North Carolina State University, Raleigh, NC 27695-8204, USA

Novel aromatic and aliphatic organic acids, esters and ketones were synthesized and assayed as repellents for ticks. *E*-7-(cyclohexyl)hept-4-enoic acid (CHEA), *E*-7-phenylhept-4-enoic acid (PHEA), ethyl *E*-7-(cyclohexyl)hept-4-enoate (CHEN) and ethyl *E*-7-phenylhept-4-enoate (PHEN) had repellent activity against the soft tick, *Ornithodoros parkeri* (Acari: Argasidae) in a two-choice bioassay. PHEN, an aromatic organic ester, was the most active. 2-undecanone, a natural product found in the trichomes of wild tomatoes, was found to mimic our lead chemistry and was active as a repellent at 50 μg/cm2. Since this compound is an already known natural botanical with a proven safety record including being approved as a food additive, formulation studies were conducted to optimize its volatility and maximize its repellent activity against ticks and insects when applied to human skin. Using proprietary emulsion technology from HOMS LLC, we were able to develop BioUD. BioUD30 and BioUD8 was a repellent to the American dog tick, *Dermacentor variabilis*, 2.5 h after applications on the skin of human subjects. BioUD8 at 6 h after application was better than OFF Botanicals with 10% PMD and equivalent to OFF with 15% DEET against mosquitoes under practical field conditions. Application of 20 μl BioUD30 (30% undecanone; 5.88mg) to ~9.8 cm^2^ of cotton fabric repelled on average >90% of *D. variabilis* ticks in a two-choice bioassay, 5 d after application, suggesting that it temporarily binds to cotton fabric. The product will be marketed by HOMS LLC as a DEET replacement. BioUD will be all natural and non-flammable unlike many DEET products, with the potential of being organically certified. The US EPA registration was submitted in March 2006 with a six months evaluation schedule.

### Expression of two related ML domain-containing proteins in gut of hard tick *Ixodes ricinus*

N. Rudenko^1^, B. Betschart ^2^, M. Golovchenko ^1^, S. Jacot^2^, J. Horaková^1^, MJ Edwards^3^, L. Grubhoffer^1^

^1^Institute of Parasitology Academy of Sciences of the Czech Republic, 37005, Ceske Budejovice, the Czech Republic. Correspondence: natasha@paru.cas.cz

^2^Institut de Zoologie, Universite de Neuchatel, CH-2007 Neuchatel, Switzerland; ^3^Department of Biology, Muhlenberg College, Allentown, PA,18104, USA.

The ML proteins family (MD-2-related lipid recognition) contains multiple members that are involved in innate immunity and lipid recognition. 155 ML proteins were identified and divided into four groups, based on the degree of sequence similarity. All ML proteins possess a putative N-terminal signal peptide (secreted or luminal proteins) and two pairs of conserved cystein residues. Two separate genes encoding ML domain containing proteins were identified in hard tick *Ixodes ricinus*. Both genes were induced in the midgut and showed 30% of identity and 46% of similarity on the protein level. The gene for allergen like protein (12.3 kDa) (AJ547805) was induced after blood feeding and the full sequence (717 bp) was isolated from the mRNA of the engorged female midgut after 5 days of feeding. The gene containing MD-2-related lipid recognition domain was strongly induced after infected blood meal feeding (ML domain containing protein, (AY323234) and its partial sequence (465 bp) was isolated from whole body subtracted cDNA library of the blood fed infected female. The signal peptide was located on the N-terminal of both proteins. Six conservative cystein residues were present in the positions 29,45,50,97,104 and 120 of the alignment. Comparison of the allergen like protein (AJ547805) and tick ML domain containing protein (AY323234) with the sequences of the related proteins from the family revealed that allergen like protein belongs rather to group II of the ML protein family that is composed of Npc2, seven mite major allergen proteins, eight *D.melanogaster* proteins (Dm ML_1–8) and five *C.elegans* proteins (Ce ML1–5). The tick ML domain containing protein was assigned to group I that contains human MD-1 and MD-2 proteins and their orthologs. The function of the gut-expressed ML proteins in tick is unknown, but it is obvious that they might be involved in host response to pathogen components and mediate defensive reactions.

### Identification and molecular characterization of novel defensin gene: the first annotation of two isoforms and the presence of introns in genomic sequence of hard tick *Ixodes ricinus*

N. Rudenko, M. Golovchenko, L. Grubhoffer

Faculty of Biological Sciences, University of South Bohemia and Department of Molecular Ecology of Parasite, Biological Center, Institute of Parasitology AS CR, Ceské Budejovice, 37005, Czech Republic. Correspondence: natasha@paru.cas.cz

A defensin gene, encoding the 8231 Da prepropeptide, 74 residues in total, including signal peptide of 22 residues and a propeptide of 15 amino acids, followed by a mature peptide of 37 residues, was isolated from the cDNA subtracted library of hard tick *Ixodes ricinus* (AY335442). Alignment of the mature region showed similarities to defensins from other species of hard ticks, ranging from 77% for *I. scapularis* to 56% for *A. hebraeum.* Similarity to 4 described defensins from soft ticks *O. moubata* (omdef-A to omdef-D) *was* 61–63% in a mature peptide. The translated sequences of different recombinants from the same cDNA library indicated the presence of two isoforms of the *I. ricinus* defensin with the approximate frequency of appearance as 4:1. The predominant form of the peptide (AAP94724) contains glutamine at position 23 (Q_23_), glutamic acid at position 25 (E_25_) and phenylalanine at position 45 (F_45_), substituted by glutamic acid (E_23_) and aspartic acid (D_25_) in the propeptide region, and arginine (R_45_) in the mature peptide in the second isoform (ABC88432). Whether these substitutions affect the properties of peptide is currently unknown. *I. ricinus* defensin gene was strongly induced only within the midgut after infection with *Borrelia burgdorferi.* Defensin cDNA was found to be 225 bp, on the basis of which the primers for genomic PCR were designed. Analysis of 926 bp of genomic sequence showed that *I. ricinus* defensin gene involves three exons, which are separated by two introns. The phase I introns splits a G_15_ codon in a signal peptide region, and R_45,_the last codon of propeptide region so, that the first nucleotide resides upstream of the intron, whereas the following dinucleotide is downstream of the intron boundary. The introns have a consensus GT/AG splice junction and a putative branch point 5′-TAAC-3′ within the ideal distance upstream of the 3′ splice site.

### Molecular taxonomic keys: Are they the solution for species identification in forensic entomology?

S. Upeka Samarakoon^1^, Steven R. Skoda^2^, Frederick P. Baxendale^1^, John. E. Foster^1^

^1^Department of Entomology, University of Nebraska-Lincoln NE, USA. Correspondence: pekas@yahoo.com

^2^USDA-ARS, Screwworm Research Unit, Panama

A functional diagnostic technique must have the ability to unambiguously identify and differentiate insect species. Insect species developing in cadavers are often used to estimate the time since death or postmortem interval (PMI). Accurate identification of the species involved is essential, but extremely difficult especially in the earlier instars because of their small size, similarity in appearance, and simplicity in external morphology. Standardization of insect molecular identification is an important process for the growth of the field as well as increasing its applicability in the field, especially for the legal process. Therefore, determination keys based on molecular genetic data complement and can generally improve the accuracy of species identification. We examined the utility of the mitochondrial Cytochrome Oxidase I (COI) and COII regions for developing a molecular taxonomic key to differentiate nine species of blow flies commonly found in Southeastern Nebraska. Primary screwworm, house fly, stable fly and fall armyworm were used as outliers in the study. Ten restriction enzymes were investigated for fragment length polymorphisms among species. The key developed from these data provides a simple three step process to compare restriction patterns and differentiate the species in question.

### The *Wolbachia* surface protein gene wspB is disrupted by a transposable element in *Culex pipiens quinquefasciatus* but not in North American *Culex pipiens pipiens* populations

Y. O. Sanogo^1,2^, S. L. Dobson^2^, S. R. Bordenstein^3^, and R. J. Novak^1^

^1^Illinois Natural History Survey, 1816 S. Oak Street, Champaign, IL-61820. Correspondence: yibosee@yahoo.com

^2^Department of Entomology, University of Kentucky; Lexington, KY 40546, USA

^3^Global Infectious Disease Program, Josephine Bay Paul Center for Comparative Molecular Biology and Evolution, The Marine Biological Laboratory, Woods Hole, Massachusetts, 02543

*Culex pipiens quinquefasciatus* Say and *Culex pipiens pipiens* Linnaeus (Diptera, Culicidae) are sibling species incriminated as important vectors of emerging and re-emerging infectious diseases worldwide. The two forms differ little morphologically and are differentiated mainly based upon ecological, behavioral, physiological and genetic traits. In their zone of sympatry, populations of *Cx. p. quinquefasciatus* and *Cx. p. pipiens* undergo extensive introgression and hybrid forms have been reported in nature. Both *Cx. p. quinquefasciatus* and *Cx. p. pipiens* are infected with the endosymbiont *Wolbachia pipientis.* To date, little is known about *Wolbachia* strain diversity in *Culex.* Here, we report the presence of a transposable element belonging to the IS256 family (IS256wPip) associated with *Wolbachia* infecting both *Cx. p. quinquefasciatus* and *Cx. p. pipiens* populations. Using comparative nucleotide analyses and reverse-transcriptase PCR, we show that IS256wPip inserted into and inactivated the *Wolbachia* outer membrane protein wspB, a paralog of the general wsp (wspA) gene in *Cx. p. quinquefasciatus.* This disruption is the first case of a recent gene inactivation associated with a transposable element insertion in *Wolbachia*. The inactivated *wsp*B was not observed in several geographically isolated strains of *Cx. p. pipiens* mosquitoes. The insertion of IS256wPip into wspB appears diagnostic of *Cx. p. quinquefasciatus* and may comprise a genetic candidate for discriminating *Wolbachia* symbionts and *Culex* subspecies.

### Biochemical and molecular mechanisms of ammonia detoxification in *Aedes aegypti* females

P. Y. Scaraffia, J. Isoe, A. Murillo and M. A. Wells

Department of Biochemistry & Molecular Biophysics and Center for Insect Science. University of Arizona, Tucson, AZ, USA. Correspondence: scaraffi@email.arizona.edu

In order to understand how mosquitoes are able to metabolize ammonia*, Aedes aegypti* female mosquitoes were fed solutions with different concentrations of NH_4_Cl or a blood meal. Amino acid analyses were carried out over time. In all cases, hemolymph glutamine and proline concentrations increased markedly, indicating that the ammonia can be removed from the body through the synthesis of these two amino acids. Aspartate, asparagine, glutamate and alanine were present in low concentrations, and the changes observed after ammonia or blood meal were less pronounced than those observed in glutamine and proline. In addition, after feeding on 80 mM NH_4_Cl, mosquitoes excreted ammonia, uric acid and urea. However, the excretion of ammonia was notably higher than that of uric acid and urea, and among the three products excreted, urea was the lowest. When methionine sulfoximine, a glutamine synthetase inhibitor, was added to the ammonia solution or blood meal, the concentration of glutamine in hemolymph decreased significantly, whereas the concentration of proline increased dramatically. In the presence of azaserine, a glutamate synthase inhibitor, the glutamine concentration increased whereas the proline concentration decreased significantly. This confirms the presence of glutamate synthase in mosquitoes, and suggests that the enzyme contributes to the production of glutamate for the synthesis of proline. Several key enzymes related to ammonia metabolism showed activity in homogenates of mosquito fat body and midgut. The mosquito genes encoding glutamate dehydrogenase, glutamate synthase, glutamine synthetase, pyrroline-5-carboxylate synthetase, and pyrroline-5-carboxylate reductase were cloned and sequenced. The mRNA expression patterns of these genes were examined by real-time reverse transcriptase-polymerase chain reaction in fat body and midgut before and after a blood meal (3, 6, 12, 18, 24, 36, 48, 72, 96 hours post blood meal). The results show that female mosquitoes have evolved efficient mechanisms to detoxify large load of ammonia.

### Kinetic of incorporation of ^15^N from labeled ammonia into amino acids in *Aedes aegypti* females

P. Y. Scaraffia^1^, Q. Zhang^2^, V. H. Wysocki^2^, J. Isoe^1^ and M. A. Wells^1^

^1^Department of Biochemistry & Molecular Biophysics and Center for Insect Science. Correspondence: scaraffi@email.arizona.edu

^2^Department of Chemistry, University of Arizona, Tucson, AZ, USA.

We have recently demonstrated that *Aedes aegypti* females are able to detoxify ammonia mainly through the synthesis of glutamine and proline along with the ammonia, uric acid and urea excretion. Now, we have established a protocol to study the kinetics of incorporation of ^15^N from labeled ammonia into glutamine (Gln), glutamic acid (Glu), alanine (Ala) and proline (Pro) in *Ae. aegypti*. Mosquitoes were fed 3% sucrose solutions containing either 80 mM ^15^NH_4_Cl or 80 mM glutamine labeled with ^15^N in either the amide nitrogen or in both amide and amine nitrogens. In some experiments, specific inhibitors of glutamine synthetase or glutamate synthase were added to the feeding solutions. At different times post feeding which varied between 0 and 96 hours, whole mosquitoes were immersed in liquid nitrogen. Whole bodies of 10 insects were homogenized in water. The suspension was centrifuged and the supernatant collected. The samples plus deuterium labeled internal standards were derivatized as dimethylformamidine isobutyl esters or isobutyl esters. The quantification of ^15^N-labeled and unlabeled amino acids was performed at a series of different neutral losses by carrying out multiple-reaction monitoring scans in a triple-quadrupole mass spectrometer (Zhang et al., 2005. J. Am. Soc. Mass Spectrom. 16:1192–1203). The results showed that the rate of incorporation of ^15^N from labeled ammonia into amino acids was rapid and that the label first appeared in the amide side chain of Gln and then in the amino group of Gln, Glu, Ala and Pro. The addition of inhibitors of key enzymes in the ammonia metabolism pathway confirmed that mosquitoes efficiently metabolize ammonia through a metabolic route that mainly involves glutamine synthetase (GS) and glutamate synthase (GltS). Moreover, a complete deduced amino acid sequence for GltS of *Ae. aegypti* was determined. The molecular signatures involved in electron donors and the previous biochemical studies (Scaraffia et al., 2005. Insect Biochem. Molec. Biol. 35:491–503) confirm that *Ae. aegypti* GltS is a NADH-dependent enzyme.

### A proteomic study of honey bee head tissue during an anti-bacterial immune response

B. Scharlaken^1^, D. C. de Graaf^1^, S. Memmi^2^, B. Devreese^2^, J. Van Beeumen^2^, F. J. Jacobs^1^

^1^Laboratory of Zoophysiology, Department of Biochemistry, Physiology and Microbiology, University of Ghent, Belgium. Correspondence: ieke.scharlaken@uGent.be

^2^Laboratory for Protein Biochemistry and Protein Engineering, Department of Biochemistry, Physiology and Microbiology, University of Ghent, Belgium.

Insects are provided with an extraordinary ability to resist infection. Their defense system relies on innate immune mechanisms. Until recently, studies on the honey bee immune system were focussed on the expression of the antimicrobial peptides. Also many proteomic studies on insect immunity were based on immune tissue (g.e. fat body) or hemolymph. Here we report a differential proteomic study that deals with head tissue, a tissue that is not immediately linked to the immune system. We developed a proteomic approach using 2D gel electrophoresis and looked for molecules that were up- or down-regulated after bacterial challenge. Approximately 60 spots were up- or down-regulated in the three time points (8h, 24h and 48h) investigated. For identification of these spots we used different mass spectrometry-based techniques. The list of identified protein spots includes an olfactory protein, structural proteins, proteins involved in signal transduction, 2 major royal jelly proteins and metabolic enzymes involved in carbohydrate metabolism, energy metabolism, protein metabolism and lipid metabolism

### Evolutionary genomics of malaria vectors

M. V. Sharakhova^1^, A. Xia^1^, I. V. Brusentsova^2^, and I. V. Sharakhov^1^

^1^Department of Entomology, Virginia Tech, Blacksburg, VA, USA. Correspondence: igor@vt.edu

^2^Institute of Cytology and Genetics, Novosibirsk, Russia.

The chromosomal model of speciation by suppression of recombination suggests that genome rearrangements promote differentiation by acting as a genetic filter between populations. Genomic regions of low recombination, such as the areas around inversion breakpoints and pericentric heterochromatin, may contain genes important for adaptations, speciation, and evolution of vectorial capacity. The availability of polytene chromosomes in malaria mosquitoes provides the opportunity to identify the evolutionary changes in the genome structure. We studied the correspondence of chromosomal elements between three malaria vectors, *Anopheles gambiae*, *An. funestus*, and *An. stephensi*, the members of different series of the subgenus *Cellia*. The *An. stephensi* cytogenetic and physical genome maps were developed and compared with the existing genome maps of *An. funestus* and *An. gambiae*. We have found preservation of synteny but substantial shuffling of gene order along corresponding chromosome arms due to paracentric inversions. Three-way analysis has allowed us to assign the rearrangement events to one of the three lineages. Using a computer algorithm we have calculated the number of rearrangements fixed between the species and identified genomic segments repeatedly occurring inside of the inversions. The analysis of the polytene chromosomes revealed extensive variations in morphology of heterochromatin among *An. stephensi*, *An. funestus*, and *An. gambiae. An. funestus* has only compact heterochromatin in the proximal centromeric region of autosomes, while the *An. gambiae* centromeric regions consist of mostly diffuse heterochromatin. The types of centric heterochromatin vary among chromosomal arms in *An. stephensi.* An antibody against the *Drosophila* heterochromatin protein 1 was used to localize the regions of intercalary and pericentric heterochromatin on the mosquito chromosomes. As a result, genomic segments that have undergone euchromatin—heterochromatin transition have been identified. Thus, comparison of chromosome structure between distant mosquito species is useful for identifying “hot spots” or “islands” of genome evolution.

### Nutritional immunology of larval *Heliothis virescens*

K. S. Shelby

USDA-ARS-Biological Control of Insects Research Laboratory, Columbia, MO, 65203 USA. Correspondence: shelbyk@missouri.edu

Dietary levels of specific essential nutrients, vitamins or micronutrients have been shown to influence a key fitness trait, immunocompetence. Selenium (Se) supplementation boosts larval lepidopteran resistance to *per os* baculovirus infection. Therefore, a study of the uptake and assimilation of vitamins C, E, Se, and other micronutrients by *H. virescens* larvae from diet was undertaken. Larvae fed diets containing 5–25 ppm Se exhibited 1) elevated plasma and tissue concentrations of Se, as measured by neutron activation analysis, 2) increased plasma virucidal activity against baculoviruses, as measured by endpoint dilution assay, and 3) higher resistance to *per os* baculovirus infection. This demonstrates that dietary Se levels are directly correlated with plasma Se levels, which are in turn correlated with baculovirus resistance. These results indicate that selenoproteins may have a role in antiviral immune response, and that identification and isolation of selenoproteins will provide insight into viral resistance mechanisms. Additional nutrients and phytochemicals have been evaluated for immunomodulatory activity. In contrast to the results with Se, Cr, Zn and ascorbate supplementation provided no significant benefit to baculovirus challenged larvae. Evaluation of the immunomodulatory effects of dietary tocopherols is now underway. Tocopherols have been shown in vertebrates to spare the Se requirement. HPLC analysis of plasma tocopherols indicates that substantial conversion of dietary plant-derived α-/δ-tocopherols into γ-tocopherol occurs in *H. virescens*.

### Larval nutrition and juvenile hormone regulate molecular components of the amino acid-target of rapamycin signaling pathway in the anautogenous mosquito, *Aedes aegypti*

S. -H. Shiao, I. A. Hansen, and A. S. Raikhel

Center for Disease-Vector Research and Department of Entomology, University of California, Riverside, CA. Correspondence: shinhong@ucr.edu

The amino acid-target of rapamycin (AA-TOR) signaling pathway plays a key role in blood meal activation of vitellogenesis and egg maturation, which further define the anautogenic nature of the mosquito *Aedes aegypti*. Here we show that the expression of essential molecular components within the AA/TOR pathway depend on attaining adequate nutritional reserves during larval development and this was further determined to be under the control of the juvenile hormone III (JHIII). By manipulating the amount of larval food, we generated two size phenotypes: “standard”, well-nourished mosquitoes, which produce eggs after the first blood meal, and “small”, malnourished mosquitoes, which require a second blood meal in order to produce eggs. Within the small mosquito, mRNA and protein expression profiles of the yolk protein vitellogenin (Vg) within the fat body were significantly delayed compare to that observed in standard mosquitoes. By topical application of JHIII shortly after eclosion, small mosquitoes were capable to produce eggs with a single blood meal along with a positive shift in Vg mRNA and protein profiles that resemble that displayed in standard mosquitoes. We further show that the quantity of nutrients attained during larval development directly affects expression profiles of the AA/TOR pathway components. The mRNA and protein expression of the insect cationic amino acid transporter 2 (iCAT2), which is at the top of the AA/TOR pathway, is delayed in small mosquitoes. This phenotype is rescued by JHIII application. Furthermore, phosphorylation of S6 kinase, a major downstream target of the AA-TOR pathway, is stimulated after a single blood meal in standard mosquitoes. This effect was only observed in small mosquitoes with JHIII application. Our results revealed that the AA-TOR signaling pathway regulates vitellogenesis directly through mosquito larval nutrition and is mediated through JHIII. Thus, our findings provide molecular evidence on how nutritional conditions during larval development mediate the anautogenic nature of adult female mosquitoes.

### Mosquito homologues of *Drosophila* dorso-ventral patterning protease Easter and its inhibitor Serpin-27A are involved in the signaling of the Toll immune pathway in the mosquito, *Aedes aegypti*

Sang Woon Shin, Guowu Bian and Alexander S. Raikhel

Department of Entomology and the Institute for Integrative Genome Biology, University of California, Riverside, California 92521, USA. Correspondence: wshin@ucr.edu

Serine protease-Serpin cassettes regulate a variety of invertebrate defense responses including hemolymph coagulation, melanization of pathogens surfaces, and signaling to immune pathways. In *Drosophila,* a clip domain serine protease, Easter, is involved in the establishment of dorso-ventral axis of the embryo by activating cleavage of a signaling ligand, Spätzle. Another closely-related clip domain protease, SPE, is reportedly required for the activation of the Toll immune pathway. A serine protease inhibitor Serpin-27A regulates Easter during dorso-ventral patterning, but not SPE during the Toll immune signaling. We have shown that the fungal-specific immune response in the mosquito, *Aedes aegypti,* involves the Toll immune pathway transduced through REL1, a homologue of *Drosophila* Dorsal. Here, we report that a Toll receptor and a cytokine ligand, AeToll5 and *Aedes* Spätzle 1C respectively, mediate the Toll anti-fungal immune signaling in this mosquito. *Aedes* homologues of *Drosophila* Easter and SPE were identified from genomic database. RNAi knock-down of an *Aedes* homologue of *Drosophila* Easter, but not of *Drosophila* SPE, resulted in decreased induction of mosquito immune genes following fungal challenge. In addition, the mosquito immune genes, which are under the control of the Toll immune pathway, were constitutively over-expressed due to RNAi knock-down of *Aedes* Serpin-27A. This strongly suggests that Easter-Serpin-27A cassette is involved in the anti-fungal Toll immune signaling in *Ae. aegypti*.

### Functionality of *Jc*DNV-derived somatic transformation vectors in insects and the role of viral enhancer sequences

P. D. Shirk^1^, R. B. Furlong^1^, J. L. Gillett^1,2^ and H. Bossin^1,3^

^1^USDA ARS CMAVE, Gainesville, Florida USA. Correspondence: pshirk@gainesville.usda.ufl.edu

^2^Present Address: Entomology/Nematology Department, University of Florida, Gainesville, FL

^3^Present Address: Entomology Unit, FAO/IAEA Agriculture and Biotechnology Laboratory, Seibersdorf Austria

Stable somatic transformation of insects following microinjection of syncytial embryos (Royer et al, 2001, J Virol. 77: 11060) or by transfection of cells lines (Bossin et al, 2003, Insect Mol. Biol. 10: 275) can be achieved by integration of entire plasmids containing the *Junonia coenia* lepidopteran densovirus (*Jc*DNV) genome. We assessed effects of sequence modifications including the presence of expression cassettes on the efficiency of *Jc*DNV somatic transformation activities in Lepidoptera and Diptera. Cloning of 3xP3EGFP outside the *Jc*DNV sequence did not affect the somatic transformation rate. Removal of coding sequences for some *Jc*DNV nonstructural proteins or the 3′ inverted terminal repeat (ITR) had no effect on the transformation rate. Removal of 177 bp from the 5′ ITR did not decrease somatic transformation rates. However, removal of a 680 bp region within the 3′ terminus of the nonstructural protein coding sequence eliminated most transcriptional activity directed by the P9 promoter. Addition of the 680 bp DNV-enhancer to JcDNV vectors lacking this sequence restored transcriptional activity. Together with previously published results, these modifications demonstrate that the somatic transformation activity is dependent upon sequences of the 3′ ITR and influenced by sequences internal to the densovirus genome. Research supported in part by USDA ARS, Exelixis & CSREES NRI to PDS

### Molecular genomics of CNS metamorphosis in *Drosophila melanogaster*

Kaitlin L. Shupe*^1^, Julia A. Z. Nimlos*^1^, Susan Miller^2^ and Linda L. Restifo^1^

^1^Arizona Research Laboratories Division of Neurobiology. Correspondence: kshupe@email.arizona.edu

^2^ARL Biotechnology Computing Facility, University of Arizona, Tucson AZ, USA. (*Authors contributed equally.)

Like other holometabolous insects, *Drosophila melanogaster* undergoes a dramatic reorganization of its central nervous system (CNS) during metamorphosis. The subesophageal ganglion separates from the thoracic ganglion, the brain fuses in the midline, and the optic lobes expand and rotate. These features of CNS metamorphosis require *Broad Complex (BRC),* a 20E-inducible primary response gene in the ecdysone cascade. It encodes a family of DNA-binding transcription factors, each containing one of four alternative zinc-finger pairs (BRC-Z1 through BRC-Z4) and having distinctive spatial and cellular domains of expression in the CNS. Genetically, BRC encompasses three subfunctions, each represented by a lethal complementation group: *reduced bristles on the palpus (rbp), broad (br),* and *lethal(1)2Bc (2Bc),* mediated by BRC-Z1, *-22,* and -Z3, respectively. We used a genome-wide approach to identify candidate BRC target genes involved in CNS metamorphosis. Using Affymetrix microarrays, we first performed a time-series analysis of wild-type CNS gene-expression profiles during −34 hours spanning the late-larval-to-early-pupal transition (−10 hr, −3 hr, 0 hr, +12 hr, and +24 hr, relative to puparium formation). Cluster analysis revealed several characteristic expression patterns. For instance, there are groups of genes induced at 0 hr, others peaking at +12 hr, and still others gradually decreasing in expression over the interval. To find BRC-regulated genes, we compared CNS gene-expression profiles of *BRC* mutants with those of a sibling control at the onset of metamorphosis. Abnormally low or high expression levels in BRC mutant CNS suggest genes which are induced or repressed, respectively, by BRC transcription factors in wild-type animals. In combination with the results of the wild-type time-series analysis, we hope to infer molecular and cellular mechanisms of BRC action during CNS metamorphosis. This project was funded by NIH grant HD38363, and JN was partially supported by HHMI #71195-521304.

### Molecular tools to study olfactory processing in the antennal lobe of holometabolous insects

Ana F. Silbering^1,2^, Daniela Pelz^1^, Tina Roeske^1^, C. Giovanni Galizia^1,2,3^

^1^ Freie Universität Berlin, D-14195 Berlin, Germany

^2^ Universität Konstanz, D-78457 Konstanz, Germany. Correspondence: giovanni.galizia@uni-konstanz.de

^3^ University of California, Riverside, CA 92501, USA

Fruit flies, as many other insects, rely strongly on their olfactory system to survive. In Drosophila, odorants are detected by olfactory receptor neurons (ORNs) housed in the sensilla on the third antennal segment and on the maxillary palps. Each receptor neuron expresses one (or few) odorant receptor genes (OR) out of a pool of ~60 G protein coupled receptors. All ORNs expressing the same receptor converge, in general, to one glomerulus in the antennal lobe (AL). AL glomeruli are also innervated by at least two populations of local interneurons (LNs), and by projection neurons (PNs). While the role of the LNs in the processing of odor information is still under debate, it is known that PNs carry olfactory information to higher brain centers, such as the mushroom bodies and the lateral protocerebrum. To investigate the detection properties of the ORNs (and thus characterize the olfactory information available for the fly) and to understand how odor information is processed in the fly brain, we have used the Gal4/UAS system to express the calcium detector GcAMP in different neuron populations along the olfactory pathway. We measured odor-evoked calcium responses in ORNs that express the olfactory receptor Or22a aiming at a comprehensive characterization of its molecular receptive range (MRR). We screened the responses to 104 odors both at the level of the sensory transduction on the antenna and of the neuronal transmission in the AL. At 10^−2^ [vol/vol] dilution, 39 odors elicited at least a half-maximal response. For these odorants we established dose-response relationships over their entire dynamic range. *Ethyl hexanoate* and *methyl hexanoate* were the best stimuli, eliciting consistent responses at dilutions as low as 10^−9^. We found no differences between the antennal and the AL MRR. Our results show that Or22a has a broad yet selective MRR, and can be functionally described both as specialist and generalist regarding its ecological role in odor detection. Next, we investigated odor-coding at a population level. We analyzed the representation of three odors across a wide concentration range within four different neuron populations innervating the AL. ORNs were labeled by means of a Gal4 line driven by the promoting region of Or83b (an ubiquitous OR required for the correct localization of ORs), two distinct LN populations were labeled using two enhancer trap lines provided by Dr. Kei Ito (NP1227 and NP2426) and PNs were labeled using an enhancer trap line generated by Dr. Gertrud Heimbeck (GH146). Our data show that, in general, higher concentrations induced increases in response amplitude and also in the number of responding glomeruli. In most cases, the sensitivity of PNs was comparable to that of ORNs, while that of the LN was shifted to higher concentrations. The dynamic range of ORNs and PNs was also broader than that of LNs. When comparing the two different LN subpopulations, differences in the spatial distribution of the responses as well as differences in their temporal dynamic were found. The combination of molecular techniques, fly genetics and genetically encoded probes for neuron activity affords the possibility of dissecting olfactory sensory processing sequentially along the cellular populations involved in it.

### Neuroanatomical organization within the octopaminergic system of the honey bee brain

Irina Sinakevitch^1,2^, Julie Mustard^2^, Brian H. Smith^2^ and Nick Strausfeld^1^

^1^ARL Division of Neurobiology, University of Arizona, Tucson, AZ 85721. Correspondence: isin@neurobio.arizona.edu

^2^School of Life Sciences, Arizona State University, Tempe, AZ 85287-4501

Octopamine plays important neuromodulatory roles in the honeybee brain. We have used a serum raised against octopamine to reveal octopamine-immunoreactive perikarya and extensive arborizations present within brain neuropils (Sinakevitch et al., 2005). Numerous and prominent clusters of lateral cell bodies in the brain as well as many midline perikarya provide octopamine-like immunoreactive processes to circumscribed regions of the subesophageal ganglion, antennal lobe glomeruli, optic neuropils, and neuropils of the protocerebrum. There are dense octopaminergic innervations in the protocerebral bridge and ellipsoid body of the central complex. The antennal lobes receive extensive octopamine-immunoreactive input, while in contrast the mushroom bodies show octopamine-immunoreactivity specifically and exclusively in their gamma lobes, which from studies of *Drosophila* have been implicated in the formation of short term memory. Octopamine acts via corresponding receptors, which include the recently clones octopamine receptor AmOAM1 from the honey bee brain (Farooqui et al., 2004). Immunohistochemistry using AmOAM1 antiserum labeled specific of cell body clusters in the brain as well as labeling of profiles within neuropils of the central complex, the mushroom body calyces, pedunculus and lobes, the antennal lobes, subesophageal ganglion, and optic lobes. Distributions of AmOAM1 do not necessarily correspond to the locations of octopaminergic processes. These findings, and the importance of octopamine and AmOAM1 distribution in the honey bee brain, will be discussed.

### Transposon-based strategies for the genetic control of *Aedes aegypti*: genetic sexing, genetic drive, and new applications with an endogenous transposable element

Ryan C. Smith^1^, Peter Arensburger^2^, Robert H. Hice^2^, David A. O’Brochta^3^, and Peter W. Atkinson^1,2^

^1^Program in Cell, Molecular and Developmental Biology, University of California, Riverside, CA 92521. Correspondence: ryan.smith@email.ucr.edu

^2^Department of Entomology, University of California, Riverside, CA 92521

^3^Center for Biosystems Research, University of Maryland Biotechnology Institute, College Park, MD 20742

Several class II transposable elements have previously demonstrated their ability to transform a wide range of insect species, including the principal mosquito vectors for malaria, dengue, and filariasis. As more and more becomes known about the vector-pathogen interactions for each of these species, the prospect for novel methods of genetic control become an increasing reality. Through the use of effector molecules to interfere with the normal cycle of disease transmission, a transgenic mosquito could combat the heavy burden of vector-borne disease upon its release into the natural population. In the event of a wide-scale release, it is necessary to release a male-only population for both social and biological reasons. To ease in the mass rearing of a male-only population, we have developed a transgenic line of *Aedes aegypti* that express the fluorescent DsRed protein under the control of the testis-specific *Aedes aegypti* β2 tubulin (*Aa*β2t) promoter. Through the use of this genetic marker, males can easily and efficiently be separated based upon the presence of DsRed expression at an early stage in development. Furthermore, once released, a gene driving strategy must be employed to ensure that the desired genetic construct can inundate a wild type population. For this reason, experiments are underway to determine whether transposases under the control of the *Aa*β2t promoter can confine appropriate transposase expression to the male germline and remobilize a *Hermes*, *piggyBac*, or *Mariner* transposon. Experiments are also underway to determine the practicality of *Buster*, a newly discovered *hAT* element from *Aedes aegypti. In vivo* transposition experiments have demonstrated the ability to transpose somatically in both *Drosophila melanogaster* and *Aedes aegypti*, and experiments are underway to determine its functionality as a transformation and gene drive vector in *Aedes aegypt*i.

### Regulation of signaling pathways in the prothoracic glands of *Manduca sexta* by nutritional factors

W. A. Smith

Department of Biology, Northeastern University, Boston, MA 02115, USA. Correspondence: w.smith@neu.edu

Recent studies in flies indicate that growth of the ecdysteroid-secreting tissues, the prothoracic glands, is a key factor in regulating body size. Previous work in our lab has shown that in *Manduca sexta*, the prothoracicotropic hormone, PTTH, stimulates tyrosine phosphorylation of prothoracic gland proteins, suggesting a growth-factor-like action. PTTH-stimulated ecdysteroid secretion appears to depend upon Src-family tyrosine kinases. More recently we have begun to investigate the stimulation of other growth regulating kinases in the action of PTTH. Using antibodies directed against conserved phosphodomains of signaling proteins, we find that PTTH does not stimulate insulin-pathway kinases such as protein kinase B/Akt. Further, the steroidogenic actions of PTTH and partially purified PTTH from pupal brain are not inhibited by PI3-kinase inhibitors that block Akt phosphorylation. The phosphorylation of the MAP kinase, ERK, and translation-regulating protein, 4EBP, are increased by PTTH and brain extract. Interestingly, removal of amino acids from the culture medium strongly reduces phosphorylation of 4EBP without affecting ERK. In conjunction, basal secretion of ecdysteroids is reduced. Similarly, starvation of larvae reduces basal steroidogenic output assessed in vitro. The glands in both cases (amino acids removed in vitro or starved in vivo) remain responsive to PTTH. When challenged with brain extract, phosphorylation of an insulin-like receptor is enhanced, in starved animals more so than feeding ones. The results suggest that starvation has a direct effect on prothoracic gland function. Further, in starved animals, insulin-responsiveness appears to be enhanced, i.e., the glands are poised to respond to the resumption of hormonal cues upon restoration of nutrients.

### IrAE – an asparginyl endopeptidase from the gut of the hard tick *Ixodes ricinus*

D. Sojka^1^, J. Dvorák^2^, M. Sajid^2^, Z. Franta^1^, O. Hajducek^1^, C. R. Caffrey^2^ and P. Kopácek^1^

^1^Institute of Parasitology, Biological Centre Academy of Sciences of the Czech Republic and Faculty of Biological Sciences University of South Bohemia, Ceské Budejovice. Correspondence: dsojka@seznam.cz

^2^ Sandler Center for Basic Research in Parasitic Diseases, University of California, San Francisco

Despite its importance, our understanding of hemoglobin digestion in ticks is still very limited and lags far behind current knowledge of this process in other hematophagous parasites. Screening of gut-specific cDNA library from the hard tick *Ixodes ricinus* resulted in isolation of a gene coding for an asparaginyl endopeptidase (legumain) designated as IrAE which is a novel member of cysteine peptidase family C13 of the CD clan. IrAE is an ortholog of asparaginyl endopeptidase from *Schistosoma mansoni*, which plays a pivotal role in the hemoglobin digestion by this parasite by trans-activation of other high-performance cysteine and aspartic peptidases (Caffrey et al., Trends Parasitol. 20: 241-8, 2004). Indirect immuno-fluorescence microscopy as well as immuno-gold electron microscopy clearly demonstrated that IrAE is present in the digestive vacuoles and also markedly enriched on the inner surface of the gut epithelium cells (within the putative perithrophic matrix). Thus, IrAE seems to be the first peptidase reported to the date to be secreted out of the tick digestive cells. A self-processing, active IrAE was expressed in *Pichia pastoris.* We use P1–P4 combinatorial fluorogenic substrate library and determined that the recombinant IrAE is strictly specific for the asparagine at the P1 position. Other characterization of IrAE enzymatic properties was performed with the use of aza-epoxide inhibitors and fluorescent activity-based probes. The pH optimum of activated IrAE for hydrolysis of small fluorogenic substrates was at pH 6.0. In contrast to it, IrAE was the most potent to trans-activate schistosomal cathepsin B1 and to cleave hemoglobin at pH 4.5. This finding provokes a tempting speculation about a “dual” role for IrAE in the neutral milieu of the tick gut contents and in the acidic digestive vacuoles. This work was supported by Grant Agency of the Czech Republic No. 206/06/0865 and research projects Nos. Z60220518, Z40550506 and MSMT 6007665801.

### Catalyzing change: Coupling foraging behavior to the environment via cGMP-dependent protein kinesis

M. B. Sokolowski, K. Kaun, C. A. L. Riedl, A T. Belay, A. K-C So and S. J. Douglas

Department of Biology, University of Toronto, 3359 Mississauga Rd. Mississauga, Ont. L5L 1C6, Canada. Correspondence: msokolow@utm.utoronto.ca

How natural selection has fashioned changes in gene function and expression to confer adaptive behavioral responses to varying environments is a central question in behavioral genetics. An excellent context to study these processes is foraging behavior, as variation can occur between genotypes in a population as well as over time within single individuals. The *foraging* (*for*) gene, encoding a cGMP-dependent protein kinase (PKG), mediates both allelic variation and plasticity in foraging behavior. In *Drosophila melanogaster*, allelic variation in *for* results in distinct larval locomotory activities that are only evident in the presence of food. In particular, larvae with a rover allele travel further on a single patch of food and move more between patches than larvae with a sitter allele. These differences may be related evolutionarily to food availability across environments. With regards to individual plasticity, PKG expression varies in a foraging-dependent manner in a number of invertebrate species, including the honeybee *Apis mellifera*, ants, *C. elegans* and *D. melanogaster*. In honeybees, plasticity is related to life history, with changes in PKG levels contributing to a transition between alternative foraging strategies. In flies, PKG expression is modulated by the nutritional state of the animal, as food-deprived rover animals show a reduction in total PKG activity, as well as decreased locomotion on food. Interestingly, well-fed sitters and *for* hypomorphic mutants also show enhanced food intake relative to rovers. Investigations into the basis of plasticity in food intake have shown that sitters are more sensitive to food-deprivation, possibly due to reduced sugar uptake, suggesting that rover/sitter locomotion differences may be related to changes in energy homeostasis. Combined with other data indicating that PKG plays an important role in learning and memory processes in invertebrates and mammals, these findings together implicate PKG as a central player bridging the environment with adaptive behavioral responses.

### Multiple paternity in a natural population of a wild tobacco fly, *Bactrocera cacuminata*, assessed by microsatellite DNA markers

Simon Song*, Dick Drew, Jane Hughes

Centre for Riverine Landscapes, Australian School of Environmental Studies, Griffith University, Australia. Correspondence: Jane.Hughes@griffith.edu.au

Mating frequency has important implications for patterns of sexual selection and sexual conflict and hence for issues such as speciation and the maintenance of genetic diversity. Knowledge of natural mating patterns can also lead to more effective control of pest Tephritid species, in which suppression programmes, such as the sterile insect technique (SIT) which would work best when wild females were monogamous, are employed. Multiple matings of females will compromise success of SIT. We investigated the level of polyandry in a Brisbane population of the field tropical fruit fly, *Bactrocera cacuminata* using seven polymorphic microsatellite loci. We genotyped the offspring of 22 wild-caught gravid females to determine the number of males siring the brood with the program Gerud2.0. Our data showed 22.7% of females produced offspring sired by two males. Paternal contributions of double sired broods were skewed with the most successful male sired between 76.9% and 87.5% of the offspring. These results have implications for SIT, because the level of remating we have identified compounds the risk that wild females will mate with one or more resident fertile males.

### TIMELESS: A link between circadian and photoperiodic clocks in the fly, *Chymomyza costata*? - The story goes on

J. Stehlik^1^, R. Zavodska^2^, K. Shimada^3^, I.Sauman^1^, V. Kostal^1^

^1^ Institute of Entomology, Biological Centre AS CR, Ceske Budejovice, Czech Republic. Correspondence: valve@centrum.cz

^2^ Faculty of Education, University of South Bohemia, Ceske Budejovice, Czech Republic

^3^ Institute of Low Temperature Science, Hokkaido University, Sapporo, Japan

A central question in our study is whether the structural homologue of the clock gene *timeless* may serve as a functional part of photoperiodic time-measuring system in the fly, *Chymomyza costata* (Diptera: Drosophilidae). A mutant strain of *C. costata* is available, in which both circadian rhythmicity of adult eclosion behaviour and photoperiodic induction of larval diapause were lost after mutation of a single autosomal gene locus *npd.* Our previous research revealed that *npd* could code for TIM protein. Here, we report about the cloning of 5′ untranslated region of *timeless* gene in *C. costata*, which revealed that *npd*-mutants carry a large deletion in the promoter sequence. Quantitation of *timeless* mRNA transcripts (real-time qPCR) in larval CNS confirmed the difference between the two strains. Clear diurnal rhythmicity was found in the wild-type CNS and the diurnal patterns differed between short-day (max at Zt16) and long-day (max at Zt24) photoperiodic regimes. Endogenous rhythmicity (under constant darkness) was detectable but relatively weak. Two neurons producing TIM protein were localized in each brain hemisphere of the wild-type larvae using specific anti-TIM antibody and the level of TIM immunoreaction showed a clear diurnal pattern. In contrary, very low transcriptional rates of *timeless* were observed in the *npd*-mutant’s CNS and no diurnal pattern was found. Similarly, no TIM protein could be detected in *npd*-mutant’s CNS. Our results indicate that *C. costata*’s *timeless* gene might be not only the structural homologue of the *Drosophila*’s *timeless* gene, but also the functional element of *C. costata*’s circadian clock. In addition, mutation in the promotor region of *timeless* gene could cause the loss of both adult circadian rhythmicity and larval photoperiodism and thus, TIM protein might represent a molecular link between circadian and photoperiodic clock systems in this fly.

### Pathogen avoidance of the insect immune response

M. R. Strand

Department of Entomology, University of Georgia, Athens GA 30602, USA. Correspondence: mrstrand@bugs.ent.uga.edu

Insects rely upon a well-coordinated innate immune system for protection against invading pathogens and parasites. Larger, multicellular parasites are usually killed by encapsulation which involves attachment of multiple layers of hemocytes to the foreign target. Smaller pathogens, in contrast, are killed by a combination of hemocyte-mediated phagocytosis and humoral defenses. Despite the fundamental importance of these responses, our understanding of their regulation in relation to the counter strategies pathogens use to evade host defense responses is limited. Viruses in the family Polydnaviridae are symbiotically associated with parasitoid wasps and are among the most virulent immunosuppressive pathogens of insects. Polydnaviruses are divided into two genera, bracoviruses (BVs) and ichnoviruses (IVs), on the basis of their association with wasps in the families Braconidae and Ichneumonidae. Genome analysis reveals important similarities in the organization BV and IV genomes but these viruses share almost no sequence homology with one another suggesting their association with parasitoids arose independently. Functional analysis has also identified several key genes involved in immunosuppression. Notably, most virulence factors encoded by polydnaviruses like *Microplitis demolitor* bracovirus (MdBV) target signaling pathways that regulate important immune effector responses rather than effector molecules themselves. Overall, these results provide important insight on the evolution of polydnaviruses and also identify key virulence determinants underlying immunosuppression.

### Action of an insulin-like hormone receptor in *Manduca sexta*

S. Subramanian, A. Walsh, and W.A. Smith

Department of Biology, Northeastern University, Boston, MA 02115, USA. Correspondence: subramanian.s@neu.edu

Insulin-like hormones such as bombyxins play critical roles in the regulation of insect growth. To further understand the role of insulin-like hormones in lepidopteran development, short-term cultures of *Manduca sexta* prothoracic glands, wing discs, and fat body, were used for initial characterization of bombyxin-stimulated phosphoproteins. In these tissues, bombyxin and bombyxin-containing brain extract stimulate a rapid increase in the phosphorylation of an 85 kD protein containing a conserved insulin receptor domain, as determined with antibodies directed against conserved phosphopeptides. In addition, bombyxin and brain extract stimulate phosphorylation of protein kinase B/Akt. The PI3-kinase inhibitor LY294002 blocks hormone-stimulated phosphorylation of downstream signaling elements such as Akt, but dramatically increases hormone-stimulated phosphorylation of the insulin receptor, suggestive of receptor up-regulation in the absence of negative feedback by signals directly or indirectly derived from active PI3-kinase. Long-term disc cultures have been used for initial knockdown of bombyxin receptor with concomitant blockade of hormone-stimulated growth. The results indicate that bombyxins stimulate lepidopteran wing growth through a typical insulin-signaling cascade, and provide tools for examining the activation of such signals in growing larvae. Funded in part by NIH grant DK53992 to WAS.

### DNA screening reveals resistance remains rare in pink bollworm after a decade of exposure to Bt cotton

B. E. Tabashnik^1^, J. A. Fabrick^2^, S. Henderson^3^, R. Biggs^1^, C. Yafuso^1^, M. Nyboer^1^, N. Manhardt^1^, L. Coughlin^1^, Y. Carrière^1^, T. J. Dennehy^1^ and S. Morin^4^

^1^Department of Entomology, University of Arizona, Tucson, AZ 85721, USA. Correspondence: brucet@ag.arizona.edu

^2^USDA-ARS, U.S. Arid Land Agricultural Research Center, 21881 N. Cardon Lane, Maricopa, AZ 85239, USA

^3^Interdisciplinary Arts and Sciences, University of Washington, Tacoma, Box 358436, 1900 Commerce St., Tacoma WA 98402

^4^Faculty of Agriculture, Hebrew University of Jerusalem, Rehovot 76100, Israel

Transgenic crops producing *Bacillus thuringiensis* (Bt) toxins reduce reliance on insecticides, but evolution of resistance by pests could cut short their usefulness. Pink bollworm (*Pectinophora gossypiella*) is a major lepidopteran pest that has experienced selection for resistance to Cry1Ac, the toxin in Bt cotton, in Arizona since 1996. In laboratory-selected strains, resistance to Cry1Ac and survival on Bt cotton are linked with three recessive mutations in the gene encoding a cadherin protein that binds Cry1Ac. Each of the three resistant alleles has a deletion upstream of the putative Cry1Ac-binding region of cadherin protein. We developed a PCR-based method for detecting each of the three resistant alleles. Screening of DNA from >5,500 field-collected insects from 58 cotton fields detected no resistant alleles. Bioassays and field efficacy tests confirm that resistance to Cry1Ac remains rare in pink bollworm after a decade of widespread exposure to Bt cotton. A synthesis of experimental and modeling results suggests that key factors delaying pink bollworm resistance to Bt cotton are refuges of cotton without Bt toxin, recessive inheritance of resistance, incomplete resistance, and fitness costs associated with resistance.

### *In vivo* and *in vitro* characterization of midgut bacteria isolated from *Aedes aegypti*

O. Terenius^1,2^, J.M. Lindh^1^, K. Eriksson-Gonzales^1^, and I. Faye^1^

^1^Department of Genetics, Microbiology and Toxicology, Stockholm University, 106 91 Stockholm, Sweden; ^2^Present address: Department of Molecular Biology & Biochemistry, 3205 McGaugh Hall, University of California, Irvine, CA 92697-3900, USA.

The burden of vector borne diseases is still enormous despite many efforts to reduce their impact. One way to limit the capacity of the vectors to transmit disease is to make them paratransgenic. A paratransgenic insect harbours midgut bacteria that are genetically modified to produce anti-pathogen effector molecules and as a result block further transmission. A prerequisite for a paratransgenic approach is a basic understanding of the bacterial population dynamics in the midgut of the vector. Therefore we investigated the midgut flora of the vector *Aedes aegypti* (Diptera: Culicidae), before and after blood feeding. It was shown that the total number of bacteria increases early after a blood meal and that the diversity of bacterial species in an individual mosquito generally is low. We also isolated both Gram-negative and Gram-positive bacteria from the same laboratory reared colony and characterized a subset of the isolates with respect to their antibiotic resistance, biochemical properties and whether they could inhibit the growth of other isolates. One of the species, *Pantoea stewartii* (*Enterobacter agglomerans*), had also been isolated from field caught *Anopheles gambiae* mosquitoes. It was possible to re-introduce the two isolates into the mosquitoes and by transforming them with plasmids expressing GFP, we could compare their sustainability in the *Ae. aegypti* colony.

### Non-random distribution of heterochromatic simple repeats; Evidence for insertion events in euchromatic region of *D*. melanogaster X-chromosome

Airlia C.S. Thompson, Susan J. Miller, Chris Bauer and Giovanni Bosco

Department of Molecular and Cellular Biology, University of Arizona, Tucson AZ, USA. Correspondence: airliat@email.arizona.edu

Extensive blocks of satellite DNA are characteristic of heterochromatic sequences in *D*. melanogaster and various other eukaryotes. Simple repeats have been shown to possess essential biological functions in this chromatin environment. The representation of these sequences in the euchromatic genome of *D*. melanogaster, however, has not been previously investigated. We hypothesize that chromosomal rearrangements throughout the evolution of *Drosophila* resulted in the insertion of blocks of heterochromatic simple repeat DNA into euchromatic regions. We used a bioinformatics approach to map the occurrence and distribution of 15 known *Drosophila* heterochromatic simple repeats as single and tandem copy regions (TCRs) in the euchromatic region of the X chromosome of *D*. melanogaster. Four specific findings from this sensitive analysis support our hypothesis, including: (i) that the heterochromatic simple repeats of interest have a non-random occurrence (ii) and distribution along the X-chromosome, (iii) that these repeat motifs co-occur with likely degenerate sequences at a higher than expected rate and, (iv) are negatively correlated with gene density. An extrapolation of this study to other *Drosophila* species will provide insight into the contribution of these sequences to genome size and structure and genetic variation within and between species. In addition, this investigation lays the groundwork for elucidation of the potential euchromatic functional roles of these simple repeats in gene regulation, recombination and other biological processes.

### Molecular genetic analysis of the gustatory receptor gene family in *Drosophila*

Natasha Thorne, Tetsuya Miyamoto, Steve Bray, Sailaja Allamneni and Hubert Amrein

Department of Molecular Genetics and Microbiology, Duke University Medical Center, 252 CARL Bldg./Research Drive, Durham NC 27710. Correspondence: hoa1@duke.edu

Most behaviors in the fruitfly *Drosophila melanogaster* are at least in part mediated by chemosensory signals. Hence, taste and olfaction present crucial sensory modalities for virtually all social behaviors including courtship, mating and aggressive behaviors, finding and identifying of food sources and recognizing and avoiding of toxic and noxious chemicals. The gustatory receptor (*Gr*) gene family encodes 68 distinct putative G-protein coupled receptor proteins that are thought to be responsible for mediating all contact chemosensory signals present in the environment, including taste cues from food sources, noxious and toxic compounds encountered in the (natural) habitat, and pheromones from conspecifics and closely related species. A large fraction of *Gr* genes are thought to encode receptors for compounds avoided by the fly (and bitter-tasting to humans), based on their complex expression profile in taste cells that are required for effective avoidance behavior, i.e. are required for the detection of various noxious tasting compounds. Despite these extensive expression analyses, specific functions of only two receptors are known: *Gr5* encodes a receptor for the sugar trehalose, and *Gr68a* is essential for efficient male courtship and is likely to encode a receptor for female pheromones. To elucidate the specific functions, ligand specificities and behavioral roles of a large number of *Gr* genes, we have initiated a large *Gr* gene knock program. Such an analysis has become feasible due to i) new gene targeting technologies introduced to *Drosophila* molecular genetics and ii) the extensive clustering of *Gr* genes in the genome. Priority for gene targeting has been given to *Gr* genes that show high evolutionary conservation and/or show intriguing expression profiles. To this end, we have generated six fly strains with single or multiple *Gr* gene deletions. Functional Analysis of some of these strains will be presented.

### Expression profiles of a large family (RR) of cuticular protein genes *in Anopheles gambiae*

T. Togawa, W.A. Dunn, J.H. Willis

Department of Cellular Biology, University of Georgia, Athens, GA, USA. Correspondence: togawa@cb.uga.edu

The physical features of insect cuticle vary among metamorphic stages and anatomical regions. These differences are accompanied by differences in the nature of the cuticular proteins, their degree of sclerotization, and the chitin content of the cuticle. Hundreds of cuticular proteins have been identified from several orders of insects, and the majority of them were classified as one large family with a conserved domain, the R&R Consensus which functions as a chitin-binding domain. Important information about the diverse roles that these proteins play in cuticle formation may come from analyzing their expression patterns in a single species. Over 130 RR proteins have been manually annotated in the *Anopheles* genome. Their expression (levels of mRNA) are being measured using real-time RT-PCR with cDNA generated from whole animals collected at regular intervals from all metamorphic stages, with special attention being paid to assuring that each primer pair amplified only a single gene. Diverse patterns of gene expression were seen. We found some genes that showed stage-specific expression and others that were expressed in all stages. Some genes were expressed at only one time point, suggesting contribution to a particular layer of the cuticle. Patterns of expression of genes clustered on a chromosome were often, but not always, identical, although levels of expression could vary greatly. We also found that expression differed by over four orders of magnitude among different genes at a single time point, or among the same gene at different time points.

### Identification, expression and properties of two small families of cuticular proteins in *Anopheles gambiae*

T. Togawa^1^, N. He^1^, W.A. Dunn^1^, V. Belozerov^2^,R. McNall^3^, J.H. Willis^1^

^1^Department of Cellular Biology, University of Georgia, Athens, GA, USA. Correspondence: togawa@cb.uga.edu, hejia@cb.uga.edu

^2^Department of Neurosurgery, Emory University School of Medicine, Atlanta, GA, USA, ^3^Centers for Disease Control, Atlanta, GA, USA.

The majority of arthropod cuticular proteins are characterized by possessing a conserved sequence, the R&R Consensus which functions as a chitin-binding domain. From MS/MS analyses of cast pupal cuticles or larval head capsules, we identified two families of proteins that lack the R&R Consensus. One (CPF) had a truncated version of the 51 aa domain first identified by S. O. Andersen in 1997. Four members of this family were found in the *Anopheles* genome. Real-time RT-PCR revealed that CPFs are expressed only at the end of larval (CPF1, CPF2) or pupal (CPF3, CPF4) stages, indicating that the proteins are components of pupal and adult epi- or exo-cuticles. Recombinant proteins of CPF1 and CPF3 aggregated, but did not bind to chitin columns. The other family (CPTC) has two conserved cysteine residues and no homology to other known proteins, i.e. no matches in the arthropod nr database. *A. gambiae* has four CPTC genes. The four CPTC proteins were abundant in the MS/MS data; indeed we obtained peptides that covered 60% of the sequence of CPTC2. Peptides from CPTC1 and CPTC4 were recovered from material that bound to chitin beads. No CPF proteins were detected in this material. The specific role of these two families of proteins in cuticle formation remains to be elucidated.

### Role of the JAK/STAT pathway in *Tribolium* oogenesis

J. Trauner, A. Cerny, M. Klingler and J. Büning

Developmental Biology Unit, Institute for Biology, University of Erlangen-Nuremberg, Staudtstrasse 5, 91058 Erlangen, Germany. Correspondence: jtrauner@biologie.uni-erlangen.de

*Tribolium castaneum* exhibits ovaries of the telotrophic meroistic type which differs fundamentally from the polytrophic meroistic ovary present in *Drosophila*. In the telotrophic meroistic ovary, nurse cells do not accompany the maturing follicles but remain located in the apical portion of the ovariole, the tropharium. The growing oocytes stay connected to the tropharium by nutritive cords. We are interested in the mechanisms of stem cell regulation, clustergenesis and embryonic axis formation in this ovary type. We have initiated loss-of-function studies of *Tribolium* oogenesis using RNA interference against *Tædomeless*, the transmembrane receptor of the JAK/STAT pathway. Depending on the developmental stage of injection, *domeless* dsRNA is able to induce phenotypes indicative of three separate functions of the JAK/STAT pathway in *Tribolium* oogenesis and early embryogenesis: germ cell proliferation, follicle formation and embryonic patterning. The phenotypes we obtained are specific to *domeless* as RNAi for the Bmp-orthologues *glass bottom boat* and *decapentaplegic* lead to completely different phenotypes. These results demonstrate the applicability of systemic RNAi for analyzing oogenesis in *Tribolium* and they identify the JAK/STAT pathway as a central player in this developmental system.

### A direct role of JH in the control of imaginal disc formation and growth in *Manduca*

J.W. Truman^1^, K. Hiruma^2^, J.P. Allee^3^, S.G.B. MacWhinnie^3^, D. Champlin^3^, and L.M. Riddiford^1^

^1^Department of Biology, University of Washington, Seattle, WA 98195-1800. Correspondence: lmr@u.washington.edu

^2^Faculty of Agriculture and Life Sciences, Hirosaki University, Hirosaki 036-8561, JAPAN

^3^Department of Biological Sciences, University of Southern Maine, Portland, ME 04103.

In Lepidoptera the eye and the leg imaginal discs form only in the final larval instar from imaginal primordia that make larval cuticle during the earlier instars but remain diploid. Formation of these discs in the tobacco hornworm, *Manduca sexta*, begins about 18 hr after ecdysis with the appearance of Broad in these cells and the detachment of the primordium, followed by the onset of proliferation by 24 hr. Starvation from the time of ecdysis prevents this formation, which can be restored by feeding on sucrose plus casein; sucrose only permits the up-regulation of Broad, but not proliferation. By contrast, these discs form and grow slowly in starved allatectomized larvae lacking juvenile hormone (JH), and this formation can be prevented by JH. Ligation experiments show that this disc morphogenesis induced by the removal of JH is independent of ecdysteroid action. Starvation experiments and JH treatment both *in vivo* and *in vitro* showed that JH acted directly on the primordia to suppress morphogenesis and that a second unidentified factor dependent on nutrients is necessary for the morphogenesis to occur. This factor that we call “metamorphosis initiating factor” appears only in the final instar and can override the JH suppression of disc formation. Thus, disc growth in the final instar is comprised of both morphogenetic growth under the suppressive control of JH and nutrient-dependent growth. One major role of JH then during larval life is to allow isomorphic growth of these imaginal primordia as the larva grows. This suppression of morphogenesis is also seen in embryos of more basal insects where premature exposure to JH suppresses embryonic patterning and induces precocious terminal differentiation. Thus, the ancient role of JH is to allow switching between growth and morphogenesis. Supported by grants from NSF to JWT and LMR, USDA to LMR, Japan Society for the Promotion of Science to KH, and Bioscience Research Institute of Southern Maine to DTC.

### Cloning of *Anopheles gambiae* antennal odorant receptors and functional expression in silkmoth cells

D. Tsikou^1^, V. Douris^1^, V. Labropoulou^1^, L. Swevers^1^, Z. Georgoussi^2^ and K. Iatrou^1^

^1^Insect Molecular Genetics and Biotechnology Group Institute of Biology, National Centre for Scientific Research “Demokritos”, 153 10 Aghia Paraskevi Attikis (Athens), Greece. iatrou@bio.demokritos.gr

^2^Laboratory of Cell Signaling and Molecular Pharmacology, Institute of Biology, National Centre for Scientific Research “Demokritos”, 153 10 Aghia Paraskevi Attikis (Athens), Greece.

The open reading frames of three odorant receptors (ORs) were cloned from a cDNA library generated from the antennae of female *Anopheles gambiae*. The corresponding ORs were expressed in a silkmoth cell line, either as authentic or fusion polypeptides containing N-or C-terminal tags and assessed in terms of their subcellular localization properties. Downstream signaling events were also examined following activation of the receptors with putative OR ligands in lepidopteran cells that were either transfected with one or more of the cloned ORs or also co-transfected with the promiscuous human G□16 protein, which mediates downstream signaling by activating the phospholipase C (PLC) pathway. The functionality of the expressed ORs was also assessed by preloading the cells with the Ca^2+^-binding indicator Fluo3, which causes the cells to fluoresce upon ligand-dependent activation of the PLC and subsequent release of Ca^2+^ from its intracellular stores. Our collective results suggest that mosquito ORs are able to couple efficiently with endogenous or heterologous G proteins in lepidopteran cells.

### Retrotransposon-induced cocoon color mutation in *Bombyx mori*

K. Tsuchida ^1^, T. Sakudoh ^2^, T. Nakajima ^1^, H. Fujimoto ^1^, H. Maekawa ^1^ and H. Kataoka ^2^

^1^ Division of Radiological Protection, National Institute of Infectious Diseases, Shinjuku, Tokyo 162-8640, Japan. Correspondence: kozo@nih.go.jp

^2^ Department of Integrated Biosciences, Graduate School of Frontier Sciences, University of Tokyo, Kashiwa, Chiba 277-8562, Japan

The yellow hemolymph and yellow cocoon are dependent on transport of carotenoids through the midgut epithelium. The genes have been identified by genetic linkage mapping based on phenotypic analysis. The *Y* gene (Yellow hemolymph), which controls uptake of carotenoids from the midgut epithelium and larvae of mutants with the +*^Y^* phenotype cannot absorb dietary carotenoids. Carotenoid binding protein (CBP) has been isolated and purified from *Y*-gene dominant silkworm. CBP contains a known lipid binding domain, the steroidgenic acute regulatory protein (StAR)-related lipid transfer (START) domain. The protein is expressed along the brush border of columnar cells in the epithelium of the midgut that is consistent with its function in aiding absorption of carotenoids. In this report, the genomic sequences of *CBP* between *Y* and +*^Y^* mutants were compared. The genomic structure of a *CBP* from two strains *Y* and +*^Y^* consisted of 7 exons separated by 6 introns spanning over 10 kb. The second exon of *Y* consisted 308 bp nucleotides, but only 139 bp of exon 2 was found from +*^Y^* genome. Moreover, +*^Y^* 2^nd^ intron was larger than *Y*, which resulted from insertion of a 2841bp retrotransposon. mRNA expression both in *Y* and +*^Y^* strains were detected by Northern hybridization, but the length of +*^Y^* mRNA is shorter than that of *Y*. RT-PCR analysis and sequencing showed that +*^Y^* CBP cDNA was amplified without exon 2. The retrotransposon insertion in exon 2 of CBP gene causes the mutation from yellow cocoon to white cocoon.

### Insect vector-parasite interactions: the innate immune response of *Rhodnius prolixus* and its implications for *Trypanosoma cruzi* life cycle

R.J. Ursic-Bedoya, C. Lowenberger

Department of Biological Sciences, Simon Fraser University, Burnaby BC V5A1S6, Canada. Correspondence: rursicbe@sfu.ca

Molecular interactions between insects and parasites play a major role in determining vector competence. *Trypanosoma cruzi*, which causes thousands of cases of Chagas disease in Latin America, is transmitted by triatominae insects. Unlike most protozoans, *T. cruzi* does not invade the insects’ salivary glands but remains in the intestinal tract and is transmitted through fecal contamination. We investigated the transcriptional response of the fat body and midgut of *Rhodnius prolixus* after immune stimulation. We injected bacteria or *T. cruzi* into the hemocoel and extracted RNA from intestines or fat body to generate three subtracted libraries. Sequencing and functional annotation revealed expressed sequence tags (ESTs) generated in response to various stimuli in all tissues, and included pathogen recognition molecules, regulatory molecules, and effector molecules. The role of insect immune responses in vectorial capacity will be discussed.

Funded by NSERC, CIHR, MSFHR

### Transcription profiles of two SCP-2-like genes in *Aedes aegypti*

I.Vyazunova, V.Wessley and Q.Lan

University of Wisconsin-Madison, Department of Entomology, Madison, WI, 53706. Correspondence: vyazunov@entomology.wisc.edu

Two SCP-2-like genes were identified in yellow fever mosquito *Ae. aegypti*. These genes demonstrated two different transcription profiles. SCP-2-like-1 gene is specific for larval stages, and SCP-2-like-2 gene is expressed during both larval and adult stages, but is not expressed in pupal stage. These genes are clearly gut-specific. The SCP-2-like-2 gene is induced by a bloodmeal.

### Partial sequence of the GABA receptor gene from the western corn rootworm, *Diabrotica virgifera virgifera*

Haichuan Wang and Blair D. Siegfried

Department of Entomology, University of Nebraska, Lincoln, NE 68583 USA. Correspondence: hwang4@unlserve.unl.edu

As a receptor for the major inhibitory neurotransmitter in insects, the GABA (gamma-aminobutyric acid) receptor is an important target for a number of insecticides including the cyclodienes. One conservative mutation of the GABA receptor gene has been associated with resistance to cyclodienes in insects. Cyclodiene insecticides were commonly used for soil treatments to control larvae of the western corn rootworm, *Diabrotica virgifera virgifera* (LeConte) (Coleoptera: Chrysomelidae), during 1940’s to 1960’s, but rapidly lead to widespread resistance. The resistance also has been shown to persist in rootworm populations for many years after the use of these compounds was discontinued. Since a GABA-receptor subunit-edcoding a dieldrin resistance mutation (Rdl) was isolated from a dieldrin resistant strain of *Drosophila melanogaster*, Rdl-like receptor genes have been found in several other insect orders. In most cases studied, resistance appears to be due to insensitivity of GABA receptor caused by a point mutation, which results in an amino acid substitution of an alanine either to serine or glycine within 2^nd^ transmembrane (M2) domain. Therefore, cyclodienes resistance historically represents an extremely important model for understanding the evolution of target site-mediated resistance to insecticides. Here, we report a partial GABA receptor sequence from *D. virgifera virgifera* that was identified using degenerate PCR and rapid amplification of cDNA ends (RACE). This partial GABA receptor sequence aligned with a GABA receptor subunit from a cyclodiene-resistant strain of *Tribolium castaneum* with 83% (329/393) identity in nucleotide sequence. Interestingly, we did not observe a common point mutation within M2 on this partial sequence. Our findings will add to the understanding of functional diversity of GABA receptor genes and mutations associated with resistance among populations of *D. virgifera virgifera*.

### *PiggyBac*-like elements PLE in the tobacco budworm, *Heliothis virescens*

Jianjun Wang^1, 3^, Xiaoxia Ren^2^, Thomas A. Miller^2^, and Yoonseong Park^1^

^1^Kansas State Univ., Entomology, 123 Waters Hall, Manhattan, KS. Correspondence: ypark@ksu.edu

^2^University of California Riverside, Dept. of Entomology, Riverside

^3^Department of Plant Protection, Yangzhou University, Yangzhou 225009, China.

Active transposable elements are self-replicating and mobile genetic elements in the genome, thus functioning as a mutagen that drives evolutionary processes by causing deleterious or adaptive mutations. The *piggyBac* element, one of the most widely used transposable elements in transgenesis of insects, was originally discovered in a cell line of cabbage looper moth, *Trichoplusia ni*. We examined the presence of similar sequences in the tobacco budworm, *Heliothis virescens*, and identified two different groups of piggyBac-like elements (PLE) and named them *HvPLE1* and *HvPLE2*. An intact copy of *HvPLE1* revealed the characteristics of PLE: inverted terminal repeats, inverted subterminal repeats, and an open reading frame encoding transposase, whereas other *HvPLE1* copies and all the *HvPLE2* copies carried disruptive mutations in the region encoding transposase. We also identified 0 to 2 bands per genome hybridized to a probe of *Trichoplusia ni piggyBac* in the genomic Southern blotting, which are different from *HvPLE1* or *HvPLE2*. Analysis of the sequences of multiple copies of *HvPLE1* and *HvPLE2* suggests that the PLEs are closely related to the *T. ni piggyBac*, of relatively young age, and independently entered the *H. virescens* genome. We also find large diversities among individuals in the insertion site of the *HvPLEs*, implying that they may still be active in transposition.

### *Methoprene-tolerant* (*Met*) gene orthologs from three mosquito species

S. Wang, A. Baumann, T.G. Wilson

Department of Entomology, The Ohio State University, Columbus OH, 43210, USA. Correspondence: wang.928@osu.edu

The *Methoprene-tolerant* (*Met*) gene from *Drosophila melanogaster* has been shown to function in juvenile hormone (JH) action. *Met* orthologs were isolated from three mosquito species, *Culex pipiens*, *Aedes aegypti* and *Anopheles gambiae* using RT-PCR and RACE-PCR techniques and were compared with *Met* and *germ-cell expressed* (*gce*), a gene having 70% homology with *Met*, in *D. melanogaster*. Sequence comparison showed that the mosquito genes are similar to one another and more similar to *gce* than to *Met*. This is also shown by both the intron numbers (7–9) and positions in these *Met* orthologs as similar to *gce* (7 introns) instead of *Met* (one intron). Phylogenetic analysis confirmed the relatedness to *gce*. PCR attempts to identify a second *Met* ortholog in each mosquito species was unsuccessful, consistent with genomic sequencing showing only a single *Met* ortholog in *Ae. aegypti* and *An. gambiae*. The results suggest that a gene duplication occurred in the evolution of higher, but not lower, Diptera, resulting in *Met* and *gce*.

### Two ancient lineages of the chemosensory protein family originate in the arthropods: gene expression patterns support a role in development

K. Wanner^1^, S. Forêt^2^ and R. Maleszka^2^

^1^Entomology Department, University of Illinois at Urbana-Champaign, Urbana, IL, USA. Correspondence: kwanner@insightbb.com

^2^Visual Sciences and Centre for the Molecular Genetics of Development, Research School of Biological Sciences, The Australian National University, Canberra, Australia.

Since their discovery in 1994, well over 100 chemosensory proteins (CSPs) have been identified from insect cDNA and genome sequences. Structural similarity to the odorant binding protein (OBP) family and histochemical localization to the sensillum lymph of some species led to the suggestion that like OBPs, CSPs function in olfaction and gustation by transporting hydrophobic ligands in the sensillum lymph. However, CSPs tend to be broadly expressed in tissues that lack sensilla and their function remains uncertain. We have identified two ancient phylogenetic lineages that are represented in the genome sequences of two arthropod species, a crustacean *Daphnia pulex* and a tick, *Ixodes scapularis*; therefore CSPs are likely distributed throughout the phylum Arthropoda. One lineage is characterized by several highly conserved amino acid motifs that are absent from the second lineage. Approximately 20 CSPs are encoded in the *Tribolium castaneum* and *Bombyx mori* genome sequences, while only 4 to 7 CSP genes occur in the *Apis mellifera, Drosophila melanogaster* and *Anopheles gambiae* genomes, suggesting expansions in some but not all insect orders. Furthermore, the expansions are specific to the lineage with the conserved amino acid motifs. CSP expression patterns in the honeybee *A. mellifera* and the moth *Choristoneura fumiferana* suggest a function in development, including molting. Preliminary results indicate that some CSPs may function in cuticle synthesis, consistent with their evolutionary origins in the arthropods.

### Evolution of endosymbiont lifestyles and genomes: Insights from an ant-bacterial mutualism

Jennifer. J. Wernegreen^1^, Seth N. Kauppinen^1^, Patrick H. Degnan^2^, Diana E. Wheeler^3^

^1^Josephine Bay Paul Center for Comparative Molecular Biology and Evolution, Marine Biological Lab, Woods Hole, MA. Correspondence: jwernegreen@mbl.edu

^2^Department of Ecology and Evolutionary Biology, University of Arizona, Tucson, AZ ^3^Department of Entomology, University of Arizona, Tucson, AZ

Many insect species rely on symbiotic bacteria for their survival and fecundity. These microbial associates include obligate, intracellular mutualists that provide nutritional functions for a wide variety of insect groups. In recent years, genome sequences have revealed the metabolic functions retained by these specialized bacteria and the underlying mechanisms that drive and constrain their coevolution with hosts. We are exploring genome dynamics and molecular evolution of *Blochmannia*, a bacterial mutualist that has coevolved with members of the ant tribe Camponotini for ~50 Myr or longer. Despite losing ~85% of genes encoded by free-living bacterial relatives, the small (706–792 kb) genomes of *Blochmannia* associated with *Camponotus* spp. retain a wide array of metabolic functions that may benefit their ant hosts. These functions include biosynthesis of many amino acids, cofactors and fatty acids, as well as sulfate reduction and nitrogen recycling. These symbiont functions may be particularly important during periods when the ant host experiences high metabolic demand but no food intake, such as metamorphosis and claustral founding. Comparing the genomes of two *Blochmannia* strains revealed differential gene deletion and disruption along symbiont lineages, yet complete stasis in the order and strand orientation of shared genes. Genomic stability in *Blochmannia* and other insect mutualists may constrain the ability of these bacteria to acquire new functions and to purge deleterious mutations. In addition, molecular analyses reveal strong effects of GC to AT mutational biases on both nucleotide and amino acid changes of nearly all insect endosymbionts. As a consequence of this mutational bias, certain *Blochmannia* genes include long (9–11 bp) homopolymeric A or T tracts, many of which contain frameshifts that would classify these loci as pseudogenes. However, we found that a substantial fraction of mRNA transcripts of these *Blochmannia* genes undergo transcriptional slippage that restores the intact reading frame. In sum, genome sequence data have shed light on the metabolic functions that mediate bacterial-insect interactions, as well as the consequences of an intracellular lifestyle on rates and patterns of bacterial evolution.

### Pathway-based approach to caste determination in honey bees: Transcriptional changes in genes involved with insulin signaling

D.E. Wheeler^1^, N. Buck^1^, and J.D. Evans^2^

^1^Department of Entomology and Center for Insect Science, University of Arizona, Tucson, AZ 85721-0036, USA. Correspondence: dewsants@ag.arizona.edu

^2^USDA ARS, Bee Res Lab, BARC E Bldg 476, Beltsville, MD 20705 USA.

A honey bee colony contains two female castes represented by one highly fecund queen and many minimally reproductive workers. Workers determine the caste fate of individuals by controlling larval diet. The process of caste determination is fundamental to the establishment of the morphologically distinct castes in highly eusocial insects that enhance the performance of queens and workers in their respective roles. Mechanisms underlying the process of caste determination can be used to test hypotheses related to social conflicts, levels of selection and evolution of polyphenisms. We have begun to test the hypotheses that insulin plays a role in caste determination in honey bees and that insulin signaling is involved in regulation of differential JH titers. We focused on the early period in larval development when developmental pathways start to diverge. Coding sequences made available through the Honey Bee Genome Sequencing Consortium allowed us to use a pathway-based approach. We used reciprocal transfers of larvae between queen- and worker-driving environments to discover the proximate changes in expression of components of the insulin signaling pathway in response to changes in diet quality. We found major changes with time and caste in 2 insulin-like peptides and the insulin receptor. One insulin-like peptide was expressed at very high levels in queen but not worker larvae. The other was expressed at higher levels in workers. The gene for an insulin receptor was expressed at higher levels in queen larvae than in worker larvae during the second larval instar, which precedes the known differences in JH levels. These results demonstrate that the insulin pathway is a compelling candidate for pursing the functional relationship between diet and the subsequent hormonal signals involved in caste determination and differentiation.

### Effect of age and oxidative stress on Hsp70 expression in the honey bee, *Apis mellifera*

J.B. Williams, S.P. Roberts, and M.M. Elekonich

Department of Biological Sciences, University of Nevada Las Vegas, 4505 Maryland Parkway, Las Vegas, Nevada 89154, USA. Correspondence: jason.williams@unlv.edu

As honey bees age they switch from in-colony tasks, such as nursing, to foraging for nectar and pollen outside the colony. Nurses rarely fly, have a relatively low metabolic rate, and experience a homogeneous colony environment. By contrast, foragers have the highest measured mass specific metabolic rates and produce high thoracic temperatures during their frequent foraging trips (thoraces average 36ºC compared to 29ºC in heads). Consequently, foragers have a six-fold higher concentration of the stress protein Hsp70 in their thoraces than their heads, as well as two-fold and six-fold higher Hsp70 levels than nurse thoraces and nurse heads. Interestingly, temperature does not induce Hsp70 expression in forager thoraces at typical flight temperatures or even after exposure to 50ºC for 1h, a temperature bees are unlikely to experience in nature. In this ongoing study, we used the metabolic differences between nurse and forager honey bees to test the hypothesis that oxidative stress, rather than temperature stress, induces Hsp70 expression in forager thoraces. We measured carbonyl content (a measure of protein oxidative damage), total antioxidant activity, and expression of Hsp70 and several antioxidant enzymes, superoxide dismutase, catalase and glutathione-s-transferase, in thoraces and heads of 9 to11 day-old foragers and nurses collected as foraging activity begins (~8–10 am), at mid-day (~12–1 pm), or at end of the foraging day (~3–4 pm). To determine the effect of a single foraging flight on tissue oxidative damage and Hsp70 expression, we examined the above stress measures on thoraces and heads of foragers that were collected just prior to leaving, or just after returning from a foraging flight at each collection period. To assess the effect of age on accrued oxidative damage and Hsp70 expression we repeated the above experiments on foragers and nurses aged 30 to 32 days.

### New insights into the molecular basis of target site resistance to insecticides

M. S. Williamson

Biological Chemistry Division, Rothamsted Research, Harpenden, AL5 2JQ, United Kingdom. Correspondence: Correspondence: martin.williamson@bbsrc.ac.uk

Molecular studies of insecticide resistance have advanced rapidly over the past decade through the cloning and analysis of cDNA and genomic sequences for the genes involved in target site and metabolic resistance mechanisms. This talk will review recent work involving three of the most important target sites in the insect nervous system; acetylcholinesterase (the target for organophosphates and carbamates), the voltage-sensitive sodium channel (DDT and pyrethroids) and the nicotinic acetylcholine receptor (neonicotinoids). Sequence analysis of these genes in susceptible and resistant strains has revealed a number of amino acid substitutions that cause insecticide insensitivity. Some of these are highly conserved across insect species (eg the L1014F ‘kdr’ sodium channel mutation), whilst others appear highly specific to certain species/insecticide combinations (eg the S331F AChE mutation conferring pirimicarb resistance in aphids). *In vitro* expression studies of these genes has allowed us to assess and confirm the functionality of the mutations that have been identified, whilst the development of sensitive PCR-based assays for detecting the mutations in crude sample homogenates enables rapid monitoring of resistance mechanisms in pest populations. Taken together, these studies have not only advanced our understanding of the molecular basis of resistance at these targets, but are also providing novel information as to the precise mode of action and insect/vertebrate selectivity of these important classes of insecticides. The diagnostic assays also offer significant practical benefits in that it is now relatively simple to genotype in a few hours, individual insects as small as aphids for multiple resistance mechanisms that could only be diagnosed by a complex series of bioassays lasting several days previously.

### Nitric oxide is necessary for maintaining *Manduca sexta* antennal lobe neuron activity and odor responsiveness

C. Wilson, T. Christensen, and A Nighorn

ARL Division of Neurobiology, University of Arizona Tucson, AZ. Correspondence: nighorn@neurobio.arizona.edu

Nitric oxide (NO) can mediate communication within the nervous system without regard to specific circuitry or synaptic contacts. The unique glomerular architecture of the primary olfactory neuropil along with the high expression of nitric oxide synthase (NOS) in this tissue, has lead to the hypothesis that NO plays an important role in the processing of olfactory information. We are using the moth, *Manduca sexta* as a model to understand the function of NO in the olfactory system. We show that enzymes involved in NO signaling, including NOS and soluble guanylyl cyclase (sGC), are expressed in subsets of neurons within the M. sexta olfactory system and, moreover, that NO is produced in olfactory glomeruli in response to odor stimulation. The function of NO in the olfactory system was examined in individual olfactory neurons with intracellular recording techniques while manipulating levels of NO signaling with pharmacological agents. Blocking NOS with either L-NAME or 7-NI resulted in changes in the behavior of both local interneurons (LNs) and projection neurons (PNs). Both PNs and LNs showed changes in baseline activity, including both increases and decreases in spike firing rate in LNs and the presence of bursts in many PNs. The odor-evoked activity in both neuron types was either missing or altered. The effects were mimicked in several neurons when sGC signaling was blocked using ODQ. However, some of the neurons that were affected by NO blockade did not contain detectable levels of sGC as measured by immunohistochemistry of the recorded and dye-filled neurons. These results indicate that NO has a variety of effects on olfactory neurons and that these effects are mediated by both sGC-dependent and sGC-independent mechanisms. This work is funded by NIH–NIDCD DC04292

### Changes in respiratory quotient and gene expression in *Megachile rotundata* prepupae during the transition from diapause to postdiapause at a constant 4^o^C

G. D. Yocum^1^, W. P. Kemp^1,2^, J. Bosch^3^, and J. N. Knoblett^2^

^1^USDA-ARS, Biosciences Research Laboratory, 1605 Albrecht Boulevard, Fargo, ND 58105 USA. Correspondence: yocumg@fargo.ars.usda.gov

^2^USDA-ARS Bee Biology & Systematics Laboratory, 5310 Old Main Hill, Utah State University, Logan, UT 84322-5310 USA

^3^Unitat d’Ecologia-CREAF, Universitat Autònoma de Barcelona, 08193 Bellaterra, Spain

Predicting pest emergence dates and forecasting crop damage levels require knowledge of the diapause termination date. This varies regionally and yearly based on environmental conditions. Similarly, synchronizing the emergence of managed pollinating solitary bees with the peak bloom of target crops is central for developing these pollinators in North America. To better understand diapause termination in *M. rotundata* in order to predict termination date and post diapause development under field conditions, and to be able to manipulate the termination date to match the bees’ emergence with the peak bloom of target crops, we initiated a series of investigations. In the first study, we examined gene expression and respiration patterns in field maintained bees. This study demonstrated that there is no sudden transition between diapause and post diapause development. Because of the complexity of multiple variables in field experiments, we next asked the question, “Can a simple constant low temperature treatment be used to accurately model gene expression and respiration patterns of field maintained insects?” The results of current investigation indicate that the level of gene expression for selected genes in diapausing and post-diapause bees is highly influenced by their thermal history. Based on our observations of the prolonged elevated levels of HSP70 expression and differences in expression patterns of HSP90, HSC70 and actin as compared to field collected bees we conclude the following: 1) A constant low-temperature regime is not an accurate model for gene expression and respiration patterns of field-collected insects; 2) Examining diapause development at several levels (e.g., organismal and molecular) and under different environmental regimes will allow us to tease apart the different components of diapause development.

### Genes and proteins of the silk filaments spun under water by the caddisfly larvae

N. Yonemura^1^ and F. Sehnal^2^

^1^Institute of Entomology, Academy of Sciences, Ceské Budejovice, Czech Republic

^2^National Institute of Agrobiological Sciences, Tsukuba, Ibaraki, Japan. Correspondence: sehnal@entu.cas.cz

Larvae of the sister orders Lepidoptera and Trichoptera produce silk from a pair of labial glands. The posterior gland section secretes in Lepidoptera (with the exception of Saturniidae) heavy chain fibroin (H-fibroin), light chain fibroin (L-fibroin), and two versions of the P25 glycoprotein. These components are stored in the gland lumen in form of a gel and rapidly converted into a solid silk filament during spinning. The pair of filaments is sealed into a single fibre with sericins produced in the middle gland section and polymerising during spinning with a delay. The process of silk protein polymerisation is not well understood but shearing and dehydration of the gel column seem to play crucial roles. Little has been known about the composition of the caddisfly silk that is spun and persists in water. We show that *Hydropsyche angustipennis* and *Limnephilus decipiens* representing two out of three caddisfly suborders express in their silk glands homologues of the H-fibroin (> 500 kDa) and L-fibroin (25 kDa) but not of P25. The conserved positions of crucial amino acid residues in the L-fibroin and at the ends of the H-fibroin indicate that these two proteins associate in the silk filament through a disulphide bridge as in Lepidoptera. The large (about 95% of the molecule) repetitive central region of the H-fibroin is hydrophobic in Lepidoptera and amphiphilic in Trichoptera. The regular distribution of hydrophilic motifs and the high content of charged amino acids probably facilitate the secretion and storage of the caddisfly L-fibroin/H-fibroin dimer in the absence of P25. Several types of short amino acid motifs are arranged in orderly fashion in four kinds of regularly reiterated repeats in the H-fibroin of *H. angustipennis* and in three kinds of repeats in *L. decipiens*. The motifs GPXGX, SXSXSXSX and GGX resemble those present in the lepidopteran and spider silks but the Ala-rich motifs, which are common in Lepidoptera and spiders, are wanting. On the other hand, the H-fibroin of *H. angustipennis* contains unique motifs such as APVVY and QPIYY and the H-fibroin of *L. decipiens* is characterised by highly charged motifs exemplified by EGGRRR. A symmetrical region of 31 amino acid residues with central Pro is conserved in both caddisfly species.

### MbIDGF, a novel member of the imaginal disc growth factor family in *Mamestra brassicae*, stimulates cell proliferation in two lepidopteran cell lines without insulin

Jun Zhang, Sachio Iwai, Taketo Tsugehara, Hana Sehadová and Makio Takeda

Division of Biofunctional Science, Kobe University, Kobe 657-8501, Japan. Correspondence: zhjunmba@hotmail.com

Imaginal disc growth factor (IDGF) is a soluble polypeptide growth factor that was first identified from the conditioned medium of *Drosophilia* imaginal disc C1.8+ cells. Working with insulin, IDGF stimulated the growth of cultured imaginal disk cells, which suggested that IDGF might function as a cofactor of *Drosophila* insulin or insulin-like peptide. Here we report a new member of the IDGF family, named MbIDGF, from the cabbage armyworm, *Mamestra brassicae*. Using a cloned cDNA of MbIDGF, recombinant MbIDGF protein was expressed in baculovirus-infected Sf9 cells and purified using a Hitrap Chelating affinity column and Hitrap desalting column. Without insulin, the recombinant MbIDGF protein stimulated cell growth of SES-MaBr-4 and NIAS-MaBr-93 cell lines that were derived from the fat bodies and hemocytes of *M. brassicae*, in a dose-dependent manner. The saturation of growth stimulation by MbIDGF was attained for the two types of cells at 80 ng/ml (0.8 nM) and 300 ng/ml (6 nM), respectively. The results suggest that MbIDGF may stimulate the growth of lepidopteran cells by a new mechanism without associating with the insulin pathway. Northern blot analyses showed that MbIDGF is expressed at all tested stages from embryo to adult. Tissue specific expression patterns of MbIDGF showed that MbIDGF is strongly expressed in the fat body, head, midgut and epidermis. Immunohistochemistry was carried out using the antibody to *P. rapae* IDGF (PrIDGF) and anti-PrIDGF antibody labeled the cytoplasm of all cells in the fat body, and goblet and column cells in the midgut. The cephalic ganglion contains IDGF-immunoreactivity (IDGF-ir) exclusively in one large neuron in each dorsolateral protocerebral hemisphere. IDGF-ir fiber ramifications were found in the corpora cardiaca that suggests MbIDGF may be released as the neurosecretory agent, besides secreted from the fat body.

### A new function for diapause hormone as a regulator of *Heliothis* diapause

Q. Zhang^1,^ W.-H. Xu^1,2^, R. J. Nachman^3^, and D.L. Denlinger^1^

^1^Department of Entomology, Ohio State University, Columbus, OH, USA. Correspondence: zhang.571@osu.edu

^2^Department of Molecular and Cell Biology, University of Science and Technology, Hefei, China

^3^Veterinary Entomology Research Laboratory, USDA/ARS, College Station, TX, USA;

Diapause hormone (DH) is best known for its role in the induction of embryonic diapause in the silkmoth, *Bombyx mori,* but the gene encoding this neuropeptide is present in other Lepidoptera as well, thus suggesting that DH also plays a role in other species. Attempts to induce pupal diapause in members of the *Heliothis/Helicoverpa* complex with DH have failed, but surprisingly, DH is highly effective in breaking pupal diapause in these moths. This result is consistent with the downregulation of the mRNA encoding DH during pupal diapause and its upregulation at diapause termination. Both DH and PTTH appear to be capable of terminating diapause and both can stimulate ecdysteroid synthesis in prothoracic glands. The exact nature of the interaction between DH and PTTH remains unresolved. We are currently evaluating the structural components of DH that are essential for activity, and we intend to use this information to develop biostable analogs and antagonists. MblDGF may be released as the neurosecretory agent, besides secreted from the fat body.

### Isolation and characterization of novel insecticidal lectins from a desert legume tree, *Palo fierro*

Guoli Zhou^1^, Alma Rosa Lopez-Laredo^2^, Ma. Refugio Robles^2^, Luz Vazquez Moreno^1,2^, Joy Winzerling^1^

^1^Department of Nutritional Science, University of Arizona, Tucson, AZ. Correspondence: guoli@email.arizona.edu

^2^Centro de Investigacion en Alimentacion y Desarrollo, Hermosillo, Sonora, Mexico.

*Palo fierro* (PF) is a wild legume tree that is a protected species indigenous only to the Sonoran desert. Preliminary toxicological experiments showed that PF seeds and seed flour are toxic to *Zabrotes subfasciatus*, a pest beetle of common beans. PF seeds inhibited larval development and adult reproduction of *Z. subfasciatus*, but flour from the seeds was not toxic to mammals. Three lectins, PF1, PF2 and PF3, with molecular weights of 45kDa, 33kDa and 66kDa, respectively, were extracted from PF seeds using carbohydrate (CHO)-affinity and size-exclusion chromatography. Feeding the purified PF lectins to *Z. subfasciatus* demonstrated that the toxicity of PF2 and PF3 is similar to that of the native PF seeds. Glycosylation analysis of PF2 using fluorophore assisted carbohydrate electrophoresis (FACE) indicated that the CHO of PF2 is N-linked and high mannose. Mass spectroscopy analysis of the CHO recognized by PF2 showed this lectin recognizes triantennary complex carbohydrates. A partial amino acid sequence of PF2 showed high similarity with the common soybean lectins, PHA-L (83%) and PHA-E (91%). A full length cDNA that encodes a PF lectin (PF-lectin1) with a 38bp 5′UTR, a 846bp open reading frame and a 140bp 3′UTR, as well as a 272bp of another PF lectin cDNA fragment (PF-lectin2), were obtained using degenerate PCR and RACE techniques. The deduced amino acid sequence of PF-lectin1 shares 86%, 39%, and 40% identity with *Robina acacia* lectin, PHA-E and PHA-L, respectively, while PF-lectin2 shows identities of 43% for *Robina acacia* lectin, 64% for PHA-E and 66% for PHA-L. Further characterization of PF lectin genes and their expression, as well as their molecular toxicological mechanisms to the pest will be studied. This project is funded by the Agricultural Experimentation at the University of Arizona and the Centro de Investigacion en Alimentacion y Desarrollo, Hermosillo, Sonora, Mexico.

### Transcription factor Broad mediates the hormone regulated morphologic change during *Drosophila* pupariation

Xiaofeng Zhou, Xiaoqun Zeng and Lynn M. Riddiford

Department of Biology, University of Washington, Seattle, WA 98195. Correspondence: xfzhou@u.washington.edu

Ecdysone triggers insect metamorphosis, but little is known about how this hormonal signal regulates the process of insect morph change. During pupariation, a *Drosophila* final (third) instar larva shortens its body length by contracting its muscles, and then narrows its epidermal cells to form a puparium. The *broad* gene, encoding a transcription factor with a BTB domain and zinc fingers, is expressed in response to a small rise of ecdysone titer in the absence of juvenile hormone during the late third instar. *broad* null alleles can survive to the wandering stage and initiate pupariation by contracting their muscles, but the epidermal cells fail to constrict. Here, we show evidence that *broad* is required for the constriction of epidermal cells, a process leading to smoothening of the puparium. We used *en*-Gal4 to drive *broad* RNAi to knock down Broad in the epidermal cells in the posterior part of each segment. During pupariation, the cells in the anterior part of each segment underwent apical constriction while the Broad-negative cells failed to constrict. An acute rise of F-actin level shortly before and during pupariation is necessary for apical constriction in the epidermal cells. However, in the Broad-negative cells, the levels of F-actin remained low, suggesting that Broad protein is required for increasing the levels of F-actin. Our data suggest that Broad may mediate the hormone-directed morph change by enhancing the expression of actin and by regulating the actin cytoskeleton through the Rho family of GTPases. Supported by NIH R01-GM60122.

### Distinct roles of Broad isoforms in regulation of the 20-hydroxyecdysone effector gene, *Vitellogenin*, in the mosquito *Aedes aegypti*

Jinsong Zhu, Li Chen, Alexander S. Raikhel

Department of Entomology and Institute for Integrative Genome Biology, University of California, Riverside. Correspondence: jinsong.zhu@ucr.edu

The *broad* (*br*) gene, which encodes a family of C2H2 type zinc-finger DNA-binding proteins, has been shown to act as a crucial member of the 20-hydroxyecdysone (20E) regulatory hierarchy in *Drosophila melanogaster* and *Manduca sexta*. Expression of the isoforms Z1, Z2 and Z4 are stimulated after blood feeding in the fat body of the mosquito *Aedes aegypti* in correlation to ecdysteroid peaks. Multiple binding sites for the BR isoforms are present in the 5′- regulatory region of the major yolk protein precursor gene, *Vitellogenin* (*Vg*). Injection of double-stranded RNA corresponding to isoform Z2 leads to a significant decrease in *Vg* expression at 24 h post blood meal (PBM). Conversely, knockdown of either isoform Z1 or Z4 results in enhanced *Vg* expression at 24 h PBM along with extended expression of *Vg* at 36 PBM, when the *Vg* transcription is normally halted. BR isoforms by themselves have no effects on the *Vg* promoter in cell transfection assays; however, isoforms Z1 and Z4 each repress the ecdysone receptor-mediated 20E activation of the *Vg* promoter, while isoform Z2 enhances activation of the *Vg* promoter by the ecdysone receptor in the presence of 20E. *In vitro* studies suggest that the effects of BR require their direct binding to the *Vg* promoter. Taken together, our results show that the BR isoforms are essential for proper activation and termination of *Vg* gene expression in response to 20E. In particular, isoform Z2 is required for the 20E-mediated activation of the Vg gene, while isoforms Z1 and Z4 serve as repressors.

### Qualitative and quantitative binding of Cry1Ac to midgut BBMV proteins from Cry1Ac-susceptible and resistant *Helicoverpa armigera* strains

Meibao Zhuang, Kristin Fencil, and Tarlochan Dhadialla

Dow AgroScience LLC, 9330 Zionsville Rd, Indianapolis, IN, USA. Correspondence: jinsong.zhu@ucr.edu

The binding of *Bacillus thuringiensis* Cry1Ac insecticidal proteins to brush border membrane vesicle (BBMV) proteins prepared from midguts of Cry1Ac-susceptible (S) and Cry1Ac-laboratory selected resistant (R; ISOC8) *Helicoverpa armigera* strains was compared to understand the basis of resistance. The solubilized BBMV proteins prepared from midguts of S and R larvae were fractionated by anion-exchange chromatography and proteins in each fraction were used for subsequent experiments. Toxin overlay assay revealed that Cry1Ac binds to several proteins in different fractions from both susceptible and resistant strains. When toxin overlay blots of Cry1Ac interactions with BBMV proteins from S & R strains were compared, the only observed difference was slightly reduced binding of the toxin to a 130-kDa protein in the R strain. No other significant qualitative or quantitative differences in Cry1Ac binding patterns were observed between BBMV from the S and R strain. Furthermore, surface plasmon resonance (SPR) analyses of real time binding of Cry1Ac to fractionated BBMV proteins revealed no difference in Cry1Ac binding to proteins in selected R and S fractions, in both total binding and binding affinity. Our results suggest that the minor differences observed in Cry1Ac binding to BBMV proteins may not fully account for Cry1Ac-resistance in the *H. armigera* resistant strain, ISOC8.

### Transcriptional regulation in cowpea bruchid guts during adaptation to a plant defense protease inhibitor

K. Zhu-Salzman^1^, J. Moon^1^, R.A. Salzman^1^, J-E. Ahn^1^, H. Koiwa^2^

^1^Department of Entomology, Texas A&M University, College Station, TX. Correspondence: ksalzman@tamu.edu

^2^Department of Horticultural Sciences, Texas A&M University, College Station, TX

Cowpea bruchid, when fed on a diet containing the soybean cysteine protease inhibitor soyacystatin N (scN), activates an array of counter-defense genes to adapt to the negative effects of the inhibitor and regain its normal rate of feeding and development. A collection of 1,920 cDNAs was obtained by differential subtraction with cDNAs prepared from guts of the 4^th^ instar larvae of scN-adapted (reared on scN-containing diet) and scN-unadapted (reared on regular scN-free diet) cowpea bruchids. Subsequent expression profiling using DNA microarray and northern blot analyses identified 94 transcript species from this collection that are responsive to dietary scN. scN-adapted insects induced genes encoding protein and carbohydrate digestive enzymes, probably to help meet its carbon and nitrogen requirements. Up-regulation of antimicrobial and detoxification protein genes may represent a generalized defense response. Genes down-regulated by scN reflected physiological adjustments of the cowpea bruchids to scN challenge. A large portion of the responsive genes, presumably involved in carrying out the counter-defense response, were of unknown function. The full-length cDNA of an scN-inducible cathepsin B-like cysteine protease was obtained. Its transcriptional response to scN during larval development contrasts with the pattern of the cathepsin L family, the major digestive enzymes. These results suggest cathepsin B-like cysteine proteases may play a crucial role in cowpea bruchid adaptation to dietary scN.

### Lipid uptake by insect oocytes

R. Ziegler, J.Isoe and M.A.Wells

Department of Biochemistry and Molecular Biophysics, Center for Insect Science, The University of Arizona, Tucson, AZ 85721. Correspondence: rolf@email.arizona.edu

Insect eggs contain large amounts of lipids, 30 to 40% of their dry weight. These lipids are important for the energy supply of the developing embryo and for the synthesis of membranes. How insect oocytes acquire these lipids is unclear. Oocytes can synthesize triacylglycerol (TAG) and phospholipids (PL) from fatty acids (FA), but the amount of FA they synthesize is very limited. Lipids must be imported into oocytes from the diet or from lipid stores in the fat body. Lipids are transported by lipoproteins, in insects by lipophorin and vitellogenin. These liporoteins are taken up by oocytes via receptor-mediated endocytosis, but estimates show that this uptake accounts only for about 10% of the lipids found in eggs. The other 90% of the lipids is thought to get into the oocyte without a protein moiety. The main lipoprotein, lipophorin, transports mostly diacylglycerol (DAG), but also FA. The weight of a *Manduca sexta* oocyte is less than 1 mg. From just before adult eclosion to 24 hrs later, they take up about 80 μhg of lipid, or about 10% of their total weight. We are not sure whether oocytes can take up intact DAG. We know they take up FA and lipophorin carries FA beside DAG. There is a lipase associated with the oocyte membrane which breakes down DAG. FA can diffuse through membranes, however, if large amounts of FA are taken up in a short time there are usually transport proteins involved. The uptake of FA by oocytes *in vitro* shows saturation kinetics, indicating that we have not just diffusion. One of the mammalian FA transporters is found in many other organisms and as it has conserved funtional domains. A clone was isolated from cDNA derived from *M. sexta* oocytes using degenerate primers. The original clone was about 500 bp. Through 5′ and 3′ RACE the entire gene was isolated. The open reading frame encodes 661 amino acids and is about 50% identical with the corresponding protein from *Drosophila*. The role of this protein in insect oocytes will be investigated.

### The expression patterns of G1 to S phase regulatory genes during larval diapause in the mosquito *Ochlerotatus triseriatus*

Sheri Zola, Savvas Pavlides, Kenneth Weir, and Steven P. Tammariello

Department of Biological Sciences, Binghamton University (SUNY), Binghamton, NY 13902. Correspondence: tammarie@binghamton.edu

The Eastern tree-hole mosquito, *Ochlerotatus triseriatus*, is abundant in the eastern US and acts as a major bridge vector of the La Crosse encephalitis arbovirus and the West Nile virus. Understanding the development of this insect, including overwintering strategies, may help to decipher the transmission of these diseases through this arthropod vector. This species has the ability to diapause both as pharate 1^st^ instar larvae (egg diapause) and as 4th instar larvae, however very little is known about the molecular regulation involved in either diapause program. Given that other insects undergo cell cycle arrest while in diapause, cell cycle status was investigated in diapausing triseriatus eggs and larvae using flow cytometry. Results from this study suggest that cell proliferation is halted at the G0/G1 phase during the larval diapause, but not during the egg diapause. Further, cells from diapausing larvae re-enter the cell cycle 4–5 days after the termination of diapause. To elucidate the molecular mechanism that controls this cell cycle arrest, we examined transcript levels of genes that are known to be important for the G1 to S phase transition in eukaryotic cells. Two genes, the transcription factor E2F1 and proliferating cell nuclear antigen (PCNA) are significantly down-regulated during the larval diapause, but not during the egg diapause, in *O triseriatus*. Here we show that a cell cycle arrest is associated with the larval diapause in the Eastern tree-hole mosquito, and we present data suggesting that the control of E2F1 expression may be linked to diapause program status in this important vector species.

